# Size-matched hydrogen bonded hydroxylammonium frameworks for regulation of energetic materials

**DOI:** 10.1038/s41467-022-34686-8

**Published:** 2022-11-14

**Authors:** Qi Lai, Le Pei, Teng Fei, Ping Yin, Siping Pang, Jean’ne M. Shreeve

**Affiliations:** 1 School of Materials Science & Engineering, Beijing Institute of Technology, Beijing, China; 2 Department of Chemistry, University of Idaho, Moscow, ID USA; 3 Beijing Institute of Technology Chongqing Innovation Center, Chongqing, China

**Keywords:** Materials for energy and catalysis, Synthetic chemistry methodology

## Abstract

Size matching molecular design utilizing host-guest chemistry is a general, promising strategy for seeking new functional materials. With the growing trend of multidisciplinary investigations, taming the metastable high-energy guest moiety in well-matched frameworks is a new pathway leading to innovative energetic materials. Presented is a selective encapsulation in hydrogen-bonded hydroxylammonium frameworks (HHF) by screening different sized nitrogen-rich azoles. The size-match between a sensitive high-energy guest and an HHF not only gives rise to higher energetic performance by dense packing, but also reinforces the layer-by-layer structure which can stabilize the resulting materials towards external mechanic stimuli. Preliminary assessment based on calculated detonation properties and mechanical sensitivity indicates that HHF competed well with the energetic performance and molecular stability (detonation velocity = 9286 m s^−1^, impact sensitivity = 50 J). This work highlights the size-matched phenomenon of HHF and may serve as an alternative strategy for exploring next generation advanced energetic materials.

## Abstract (web summary)

Size matching molecular design utilizing host-guest chemistry is a general and promising strategy for seeking new functional materials. Here the authors screen different sized nitrogen rich azoles for selective encapsulation into a hydroxylammonium framework to achieve a dense packing and higher energetic performance.

## Introduction

The 120-year-old Nobel prize has witnessed a long-term blossoming of human society in science and technology^1,2^. In contrast, energetic materials, the research field to which Alfred Nobel dedicated his entire life, have been recently promoted by the fast development of multidisciplinary science. While most synthetic approaches to traditional energetic molecules via limited covalent bond formation encounter an intrinsic contradiction between energetic performance and safety issues, the newly emerging strategies are inspired by innovative advances from versatile chemical communities, e.g., co-crystallization from medicinal chemistry, stereo-/regio-selective synthesis from organic chemistry, explosive spin crossover (ExSCO) complexes from coordination chemistry and metal-organic frameworks (MOFs) from reticular chemistry^3–10^. Furthermore, the achievements with metallic hydrogen and polynitrogen encourage scientists strongly to seek diversified approaches to push the limits of energy^11,12^.

Among these cross-disciplinary achievements, taming of metastable energetic molecules in specific porous frameworks becomes one of the most forward and promising investigations to desensitize energetic materials while concomitantly retaining excellent energetic properties. There are two strategies to encapsulate guest energetic moieties into host frameworks: (1) non-energetic hosts for the adsorption of energetic guests (e.g., MOF-5, tetranitromethane (TNM) and ZIF-8-TNM) realizes molecular scale mixing of fuel and oxidizer; and (2) the assembly of energetic hosts and energetic guests enabling the encapsulation of oxidizer in an N-rich framework (e.g., EMOF (Cu) and HOF-NF)^13–16^. Nevertheless, with respect to both strategies, the unfavorable components of heavy metal and halogen decrease the integrated energetic performance^17^. Furthermore, high carbon content of organic ligands leads to low energy density and negative oxygen balance, indicating incomplete energy release during the decomposition process. As a result, stabilization of energetic ingredients by encapsulation of host frameworks often occurs with the loss of energetic properties, the highly efficient encapsulation may help to solve the above contradiction.

**Fig. 1:**
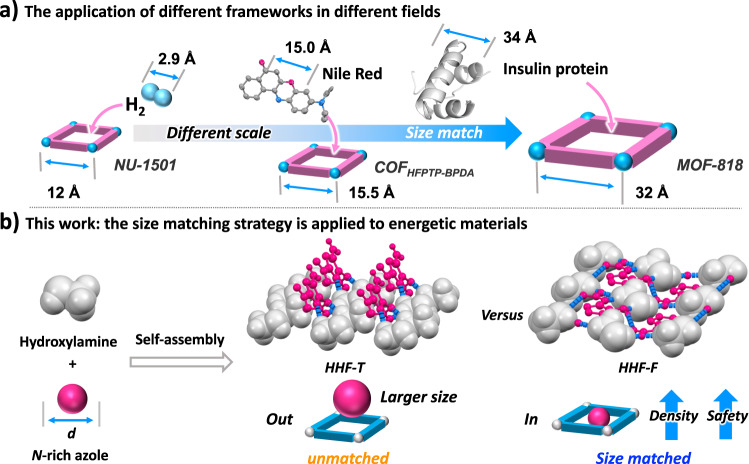
The application of size matching strategy. **a** The application of different frameworks in different fields (NU-1501^20^; COFHFPTP-BPDA^21^; MOF-818^22^); **b** Size-matched hydrogen bonded hydroxylamine framework in energetic materials (HHF-T, hydroxylammonium 3-amino-5-nitro-2-nitramino-1,2,4-triazolate; HHF-F, hydroxylammonium 3-amino-4-nitramino-furazan).

Scientists can use reticular chemistry for the fabrication of a stable framework material like ‘taking Legos’^18–21^. Moieties ranging from hydrogen molecules to proteins can be stored and selectively adsorbed using different-sized frameworks (Fig. 1a)^22–24^. As a rule, the size of each component in the framework material has a significant impact on adsorption, catalysis, recognition, separation, and other properties^25–30^. Inspired by the size-matched assembly of host-guest type materials, we propose that the match of a nitrogen (N)-rich host framework with a high-energy guest molecule is beneficial in balancing the energy density and sensitivity by tuning the assembly type. Now, a promising design strategy, i.e., the size matching of an N-rich azole guest and a hydroxylammonium host is suggested to form a hydrogen bonded hydroxylammonium framework (HHF).

Hydroxylammonium as a building block often possesses shorter hydrogen bond lengths, which has advantages in high-density energetic materials construction^31^. The representative milestone energetics, dihydroxylammonium 1,1′-diolate-5,5′-bistetrazole (TKX-50), dihydroxylammonium 5,5′-dinitro-1H,2′H-[3,3′-bi(1,2,4-triazole)]-1,2′-bis(olate) (MAD-X1), and hydroxylammonium 5-(4-amino-furazan-3-yl)-1-hydroxytetrazolate have considerable application potential with ideal balance of performance and sensitivity^32,33^. From the intrinsic viewpoint at the molecular crystal level, hydroxylammonium as an H-donor moiety participates in a hydrogen bonded framework with electron-deficient groups, resulting in more mutual penetration to achieve an improved sensitivity and a better packing coefficient. Meanwhile, the N-rich guests are embedded into the pore formed by the hydroxylammonium cations, so as to avoid close contact between sensitive groups. More importantly, the layered crystal structure will endow these energetic materials such as 1,3,5-triamino-2,4,6-trinitrobenzene (TATB) with a good packing coefficient and low mechanical sensitivity to external mechanical stimulation through π-π interaction^34,35^. The intermolecular assembly guided by the size-matched hydrogen bonded Hx framework strategy provides a stabilization scheme for energetic molecules.

## Results and discussion

### Construction of a hydrogen bonded hydroxylammonium framework (HHF)

**Fig. 2:**
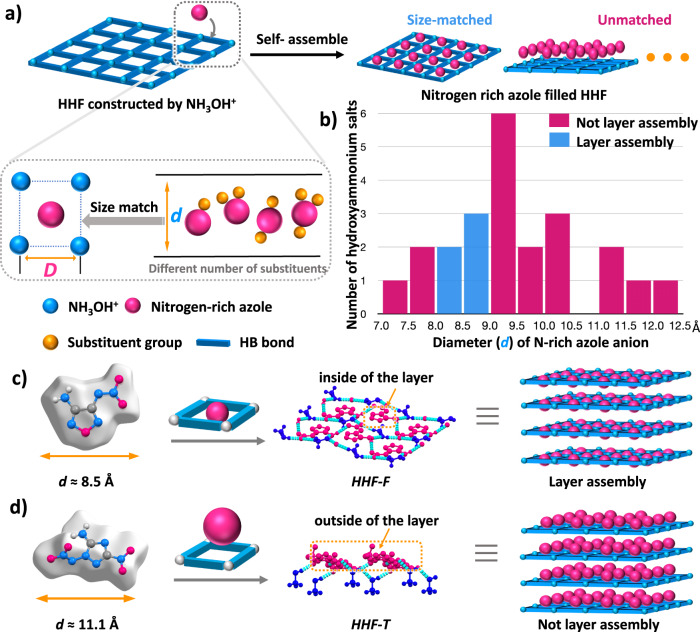
Construction of hydrogen bonded hydroxylammonium framework. **a** Illustration of possible size-matched between hydrogen bonded hydroxylammonium framework and nitroamine N-rich azoles. **b** The diameter for N-rich azole anion of energetic hydroxylammonium salts (23 hits from Cambridge Structural Database (CSD)). **c** Layer assembly mode of HHF-F and its crystal packing diagram. **d** Layer assembly mode of 3-amino-5-nitro-2-nitramino-1,2,4-triazolate (HHF-T) and its crystal packing diagram.

Through hydrogen bond self-assembly, energetic guest molecules are expected to be encapsulated into planar layered structures and the sensitive moiety (nitroamine nitrogen-rich azole) is surrounded by a hydroxylammonium framework. To achieve this goal, it is necessary to design and screen the size of the guest anions (Fig. 2a). By analyzing the reported crystal data of more than 20 monocyclic hydroxylamine-based derivatives (a-w, Supplementary Fig. 37) including dihydroxylammonium 1,5-di(nitramino) tetrazolate (**l**), hydroxylammonium 2-amino-5-nitramino-1,3,4-oxadiazolate (**r**) and other N-rich azole hydroxylammonium salts, it was found that most of the N-rich azoles do not display a layer-by-layer assembly, but possess a relatively complex and chaotic crystal stacking mode (Supplementary Fig. 39). By comparing the sizes of N-rich azoles in the crystals, we believe that the above phenomenon might be caused by the size mismatch between N-rich azoles and hydroxylammonium. When the N-rich azole size is in the range of 8 to 9 Å, such parallel face-to-face arrangements were also discovered in those crystal structures, as shown in Supplementary Fig. 38. To verify the above conclusions, in this work, we designed and synthesized two nitroamine-based derivatives of N-rich azoles with different sizes. HHF-F (hydroxylammonium 3-amino-4-nitramino-furazan) can be obtained in high yield (71%) by a one-step reaction with the commercially available diaminofurazan as the raw material. The crystal data show that HHF-F with smaller size possesses a planar structure with a highly ordered structure formed in a layer-by-layer assembly (Fig. 2c). However, the larger hydroxylammonium 3-amino-5-nitro-2-nitramino-1,2,4-triazolate (HHF-T) shows a sandwich-like crystal packing model, implying that nitrogen-rich azole is out of the hydroxylammonium framework (Fig. 2d.)

Further analysis of the crystal shows that HHF-F displays a parallel face-to-face arrangement, with neighboring nitroamino and amino groups as hydrogen bonding donor and acceptor units. Nitroamino-substituted furazan rings are well surrounded by the hydroxylammonium framework in the layer, and substituents in the ring form hydrogen bonding frameworks with the hydroxylammonium unit through ion-assisted hydrogen bonding interaction (Fig. 2c). Due to the ordered packing described above, the density and packing coefficient of HHF-F are 1.835 g cm^−3^(170 K) and 77.7%, respectively, which are superior to those of the molecular compound (3-amino-4-nitramino-furazan, 1.737 g cm^−3^, 71.5%).

**Fig. 3:**
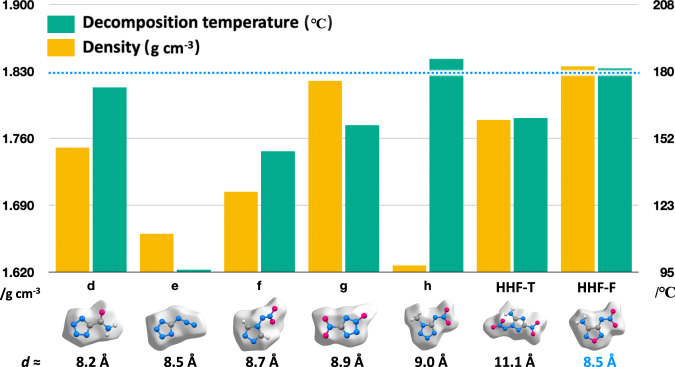
Density and decomposition temperature comparison of HHF-F with other hydroxylammonium salts. Decomposition temperature (green bar) and crystal density (yellow bar) comparison of HHF-F with HHF-T and reported monocyclic N-rich azole hydroxylammonium salts (d-h) which have diameter sizes ranging in 8-9 Å based on Hirshfeld surfaces (see details of all the reported monocyclic N-rich azole hydroxylammonium salts in Supplementary Table. 23).

The stacking distance of HHF-F (3.338 Å) is remarkably shorter than that of HHF-T (3.495 Å). Consequently, the packing coefficient of HHF-T was lower (74.4%), resulting in lower density (1.758 g/cm^−3^). Compared with the reported monocyclic N-rich azole hydroxylammonium salts (d-h) with layered stacking, HHF-F showed significant improvement in density and thermal stability (Fig. 3). The examples which do not contain -NO_2_ and -N_3_ groups in the azole ring usually possess a lower density despite higher decomposition temperatures (d, h) (Fig. 3). Thus, the size-matched strategy possesses good potential application for improving density of energetic materials.

### Energetic properties

**Fig. 4:**
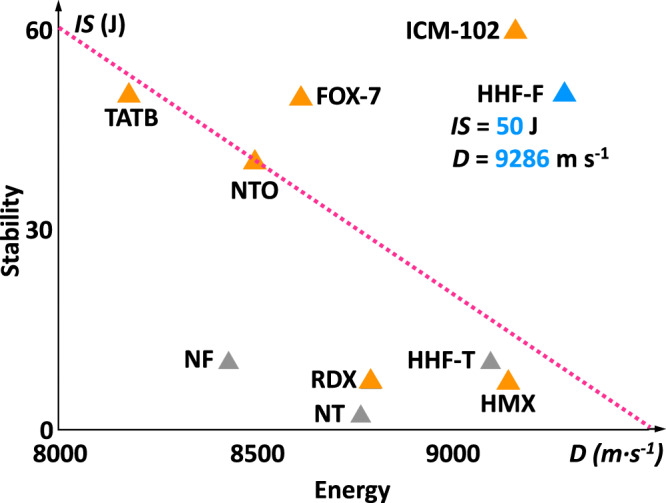
The properties comparison of HHF-F with other HEDMs. Stability (impact sensitivity) and energy (detonation velocity (calculated)) of compounds NT, HHF-T, NF, HHF-F compared with TATB, RDX, NTO, FOX-7, ICM-102, and HMX.

The detonation performance of HHF-F and other compounds in this work were calculated using EXPLO5 (v 6.05)^36^. According to the calculated heat of formation (182.85 kJ mol^−1^) and single crystal density of 1.819 g cm^−3^ (298 K), the detonation velocity (D) and detonation pressure (P) were calculated to be 9286 m s^−1^ and 35.85 GPa, respectively (Supplementary Table 20). Because of the large difference in density (1.819 g cm^−3^ vs 1.737 g cm^−3^), the detonation velocity of HHF-F is 850 m s^−1^ higher than that of neutral compound (3-amino-4-nitramino-furazan, NF), 8433 m s^−1^). Although compared with its molecular form NT (1.758 g cm^−3^, 8769 m s^−1^), the compound HHF-T (1.779 g cm^−3^, 9099 m s^−1^) showed a small increase in density and detonation performance. But HHF-F with a more ordered stacking model also displays higher stability and shows a better detonation performance compared with HHF-T. The constant-volume combustion heat (Δ_c_U°) measurement of HHF-F was carried out by oxygen bomb calorimetry, and the test value of Δ_c_U° is 9.23 MJ kg^−1^. The experimental enthalpy of formation (∆_f_H°) of HHF-F was calculated from Δ_c_U° to be −4.59 kJ mol^−1^. The detonation velocity and pressure value of HHF-F are higher than those of cyclotrimethylenetrinitramine (RDX, Fig. 4), which suggests their potential application as high-energy-density materials (HEDMs).

### Thermostabilities and mechanical sensitivities

The sensitivity of the compound HHF-F was obtained by standard BAM tests. Using DSC the thermostability of HHF-F was determined to give the thermal decomposition temperature (T_d_) at 181 °C. Comparison of T_d_ values of monocyclic nitrogen-rich hydroxylammonium salts reported in the CCDC database (Supplementary Table 23) shows that a T_d_ greater than 170 °C is relatively rare. Analysis of two examples with higher thermal decomposition temperatures demonstrates that the thermal stability of compounds is improved by introducing alkyl groups (h, q, see detail in Supplementary Fig. 37). Unfortunately, the alkylation strategy usually results in a significant decrease in density^37,38^. It is difficult to improve the thermal stability for monocyclic compounds without large conjugated structures^39–43^. However, HHF-F achieves a crystal density greater than 1.8 g cm^−3^ when the T_d_ is greater than 180 °C, which reflects the effectiveness of size-matched HHF strategy in improving the stability of compounds (Fig. 3).

**Fig. 5:**
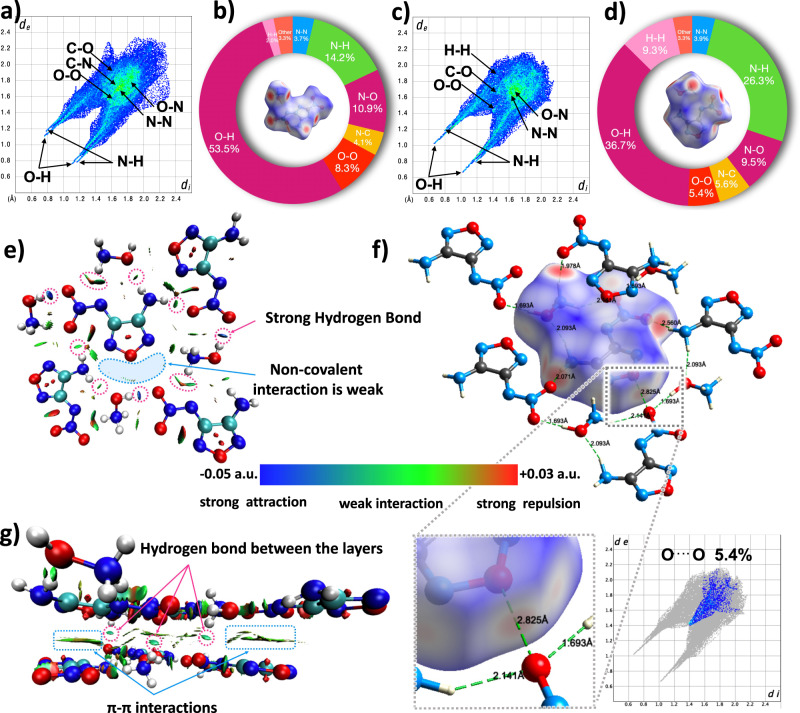
The weak interactions in HHF-F. The 2D fingerprint plots in crystal stacking for HHF-T **a** and HHF-F **c**; Pie graphs for HHF-T **b** and HHF-F **d** show the percentage contributions of the individual atomic contacts to the Hirshfeld surface, Hirshfeld surfaces (inside). **e** Noncovalent interaction (NCI) analysis of the layers in HHF-F. **f** Two-dimensional fingerprints and Hirshfeld surfaces for HHF-F. **g** Noncovalent interaction (NCI) plots between the layers in HHF-F.

The friction sensitivity and impact sensitivity of HHF-F are 50 J and 360 N, respectively. Compared with the neutral molecule NF (10 J, 160 N, see detail in Supplementary Table 20), HHF-F is very insensitive. The reported isomer hydroxylammonium 2-amino-5-nitramino-1,3,4-oxadiazolate (r) has poorer sensitivity with a lower thermal decomposition temperature (8 J, 144 N, 138 °C). The two isomers have the same explosophores, but the distinct size is greatly affected by the difference of substituent positions. Due to the size mismatch, the 1,3,4-oxadiazole unit extrudes into the interlayer position formed by the hydroxylammonium, and the hydrogen bonded framework structure that effectively separates the oxadiazole structure by the hydroxylammonium cation is not formed. A closer distance (2.7 Å) of the nitro O^…^O in hydroxylammonium 2-amino-5-nitramino-1,3,4-oxadiazolate without a hydrogen bond is the possible reason for its high sensitivity (see detail in Supplementary Fig. 40). The hydrogen bonded framework formed by the hydroxylammonium structure of HHF-F effectively reduces the O^…^O interaction between the furazan rings (5.4%, Fig. 5d). This was demonstrated by the analysis of non-covalent interactions, two-dimensional (2D) fingerprints and Hirshfeld surfaces for HHF-F (Fig. 5e, f). According to the analysis of noncovalent interactions for HHF-F, the strong hydrogen bond between the hydroxylammonium cation and the 4-amino-5-nitramino-furazan was observed in the plane of the crystal. There are also ${\pi-\pi}$ interactions between layers and interlayer hydrogen bonds (Fig. 5g). Interlayer hydrogen bonds are beneficial to the stability of the crystal and the shortening of interlayer spacing. The above comparison shows that the ordered packing has a significant effect on the properties of energetic materials, and also indicates the impact of hydrogen bonded frameworks on improving the stability.

**Fig. 6:**
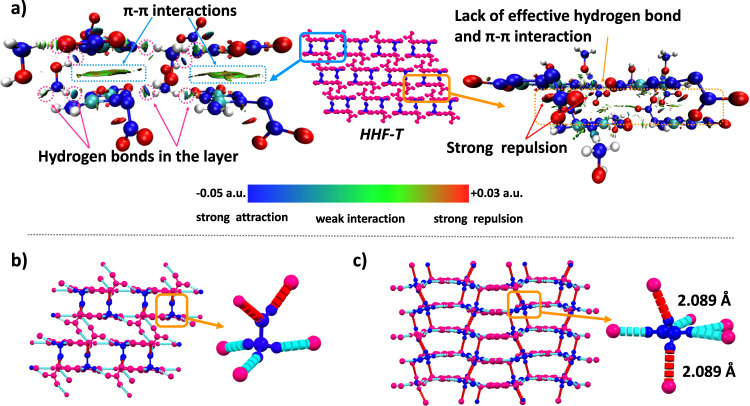
The weak interactions in HHF-T. **a** Noncovalent interaction (NCI) plots of HHF-T. **b** 3D network of HHF-T, and the hydrogen bonded environment of the hydroxylamine cation; **c** 3D network of HHF-F, and the hydrogen bonded environment of the hydroxylamine cation. (The hydrogen bond in the layer is blue, and the hydrogen bond between layers is marked as red).

From the perspective of improving the energy of energetic materials, the trisubstituted triazole ring can be substituted with more nitro groups than disubstituted furazan rings. Comparison of the two neutral molecules NT and NF prepared in this work indicates that the trisubstituted triazole has a relatively higher density (NT: 1.758 g cm^−3^; NF: 1.737 g cm^−3^), but both of them have high sensitivity and poor thermal stability (NT: IS = 2 J, FS = 60 N, T_d_ = 135 °C; NF: IS = 10 J, FS = 160 N, T_d_ = 126 °C). However, as a result of the size mismatch of triazoles in HHF-T, the 1,2,4-triazole is not in the layer formed by the hydroxylammonium moiety, but is squeezed into the interlayer and displays lower stacking efficiency compared with HHF-F (2: 74.4%; HHF-F: 77.7%; Fig. S5). It also shows high sensitivity (IS = 10 J, FS = 160 N) due to the short distance between nitramino groups, which can be obtained through the analysis of non-covalent interactions (given in Fig. 6a). According to the hydrogen bond environment analysis of hydroxylammonium cations in the crystal data, more hydrogen bonds are formed when hydroxylammonium and N-rich azoles are located in the same layer (Fig. 6c), and more hydrogen bonds are also conducive to improved stability. More importantly, the hydroxylammonium in HHF-F could form hydrogen bonds with both intra- and interlayer N-rich azoles, which is attributed to their suitable size. In addition, this layered structure has been shown to be more conducive to sliding in response to external mechanical stimuli, resulting in improved mechanic stability of the energetic materials.

**Fig. 7:**
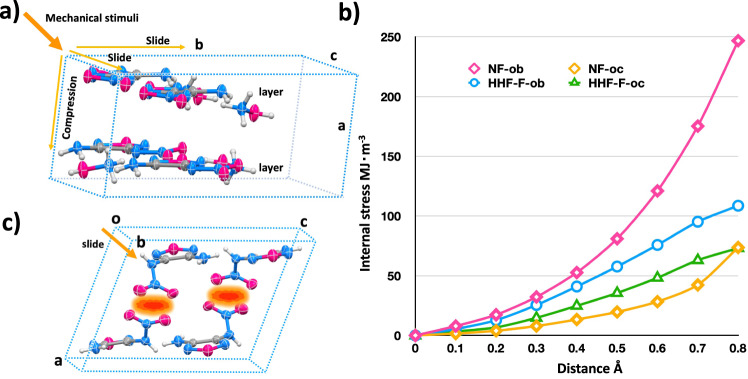
Analysis of energy changes before and after molecular deformation for HHF-F and NF. **a** Model for external forces acting on HHF-F crystal unit cell. **b** Internal stress curve along the b and c axes for HHF-F (HHF-F-ob and HHF-f-oc) and NF (NF-ob and NF-oc). **c** Plot showing the sliding constraint of the NF crystal unit cell along the b axis.

It is generally believed that crystals with a layer-by-layer stacking mode can be cushioned by interlayer slides when subjected to external forces^44^. For a deep understanding of the desensitizing mechanism of size matched induced layer by layer packing, a force field was established for HHF-F and NF, and we used a deformation potential (for convenient comparison, the value is converted from mol units into volume units by dividing by the unit cell volume, see details in Supplementary Tables 21 and 22) to describe their energy changes before and after molecular deformation based on their unit cell geometries (Fig. 7b). The results display a significant difference in deformation potential energy between the neutral compound NF and the hydroxylammonium salt HHF-F. The maximum energy change caused by the neutral molecule sliding along the b axis (0–246.7 MJ m^−3^), and the horizontal sliding of HHF-F causes energy changes in the range of 0–108.6 MJ m^−3^, indicating that the slide is severely limited for NF caused by its disordered packing mode in the crystal (Fig. 7c). Therefore, the conclusion is that HHF-F with a layered structure can easily absorb mechanical stimuli, converting kinetic energy into interlaminar sliding and preventing the formation of hot spots (Fig. 7a)^45,46^. This calculation analysis partially explains why HHF-F has a lower mechanical sensitivity than NF. It means that the size matched hydrogen bonded Hx frameworks strategy is feasible to improve the stability of energetic materials while ensuring their energetic properties.

For N-rich azole anions, the combination of different skeletons and substituents can get various structures with different sizes. In general, for the monocyclic systems studied in this work, their dimensions are mostly in the range of 7–15 Å.

According to the source classification of N-rich azoles acid hydrogen for energetic hydroxylammonium salts in the CSD database, the most abundant ones are nitrosamine anionic guest and heterocyclic acid hydrogen guest, respectively. The acidic hydrogen of HHF-F and HHF-T reported in this work is derived from the nitroamine based structure.

**Fig. 8:**
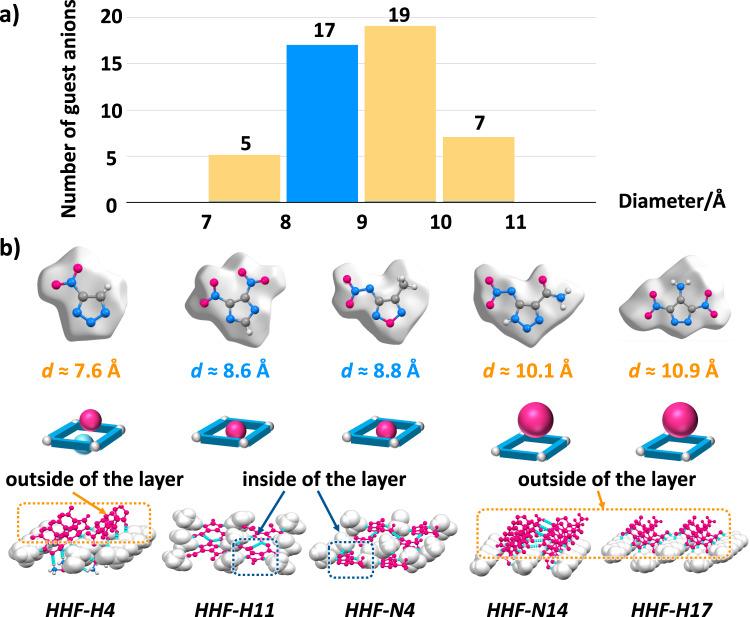
The further application of the size matching strategy. **a** The predicted diameter for N-rich azole anion (48 hits). **b** Layer assembly mode of HHF-H4, HHF-H11, HHF-N4, HHF-N14, HHF-H17, and crystal packing diagram (Red balls represent the guest anions, whereas the blue ball in HHF-H4 represents the cocrystallized ammonium oxide).

For the further application of this strategy, we constructed the nitroamine guest based on the monocyclic N-rich azoles as the skeleton and a series of anion guests formed by N-rich heterocyclic azoles with acid hydrogen. To adjust the size of guest anions, two candidate libraries of guest anions are established by introducing different substituent groups. We have predicted the size of these guest anions by theoretical calculations and present the statistical results in Fig. 8a (see details in Supplementary Figs. 44 and 51), and by predicting the size of these guest anions to guide us in obtaining the next such HHF system (HHF-H11 and HHF-N4, Fig. 8b). By analyzing the relationship between the guest molecule size and the crystal stacking structure of 7 examples obtained in this work, it can be concluded that the guest molecules of HHF-F (8.5 Å), HHF-N4 (8.8 Å), and HHF-H11 (8.6 Å), which have guest molecule sizes of 8–9 Å, are embedded in the hydroxylammonium composed of hydrogen-bonded frameworks and exhibit layer-by-layer stacking. In contrast, the other over- or undersized guest molecules of HHF-N14 (10.1 Å), HHF-H17 (10.9 Å), HHF-T (11.1 Å), and HHF-H4 (7.6 Å) display a more disordered crystal structure. This simple method to design energetic materials based on the size-matched strategy could promote an ordered hydrogen-bonded assembly between energetic molecules, thus displaying layer-by-layer packing in a solid state and forming a hydrogen bonded framework.

In summary, we have developed a simple method to design high-performance low-sensitive energetic materials based on the size matching strategy to promote an ordered hydrogen bonded assembly between energetic molecules, thus displaying layer by layer packing in a solid state and forming a hydrogen bonded framework. By screening different-sized N-rich azoles, an energetic guest (3-amino-4-nitramino-furazan) is encapsulated into the hydrogen bonded hydroxylammonium framework, giving rise to a new energetic material HHF-F. Analysis of crystal structures and modeling calculations show that the layer-by-layer geometry of HHF-F can transform kinetic energy into layer-by-layer sliding, which can easily absorb mechanical stimuli to reduce sensitivity. It shows a good detonation performance (D = 9286 m s^−1^; P = 34.95 GPa), excellent sensitivity (IS = 50 J; FS = 360 N) comparable to RDX, and better thermal stability (T_d_ = 181 °C) than reported monocyclic N-rich azole hydroxylammonium salts. In this study, the crystal packing pattern was regulated by the design of molecular size, which provides a new way for the development of novel HEDMs.

## Methods

Caution! The prepared compounds are energetic materials and may explode under certain conditions. Appropriate safety precautions should be taken when preparing and handling.

### General

All reagents were purchased from Energy Chemical or Aladdin in analytical grade. ^1^H and ^13^C NMR spectra were recorded on Bruker AVANCE 400 nuclear magnetic resonance spectrometer. DMSO-d_6_ was employed as a solvent and locking solvent. Infrared (IR) spectra were recorded on an FT-IR spectrometer (Thermo Nicolet AVATAR 370). Decomposition (onset) temperature were recorded on a differential scanning calorimeter (DSC, TA Instruments discovery DSC25) at a scan rate of 10 °C min^−1^. Elemental analyses (C, H, N) were performed on a Vario Micro cube Elementar Analyzer. Impact and friction sensitivity measurements were made using a standard BAM Fallhammer and a BAM friction tester.

### N-(5-Amino-3-nitro-1H-1,2,4-triazol-1-yl)nitramide (NT)

To a solution of 3-nitro-1H-1,2,4-triazole-1,5-diamine (10 mmol, 1.44 g) in conc. H_2_SO_4_ (20 mL), ammonium nitrate (20 mmol, 1.60 g) was added at −10 °C. The reaction was stirred vigorously at −10 °C until a transparent solution was obtained. The resulting reaction was then quenched by ice (ca. 100 g) and the aqueous layer was extracted with ethyl ether (3 × 50 mL). The combined organic layers were dried over Na_2_SO_4_ and the solvent was removed by rotary evaporation to give NT (87% yield). ^1^H NMR (400 MHz, DMSO-d_6_) δ 7.37–7.29 (m, 3H); ^13^C NMR (101 MHz, DMSO) δ 158.3, 156.5; IR (KBr pellet): υ 3437, 3320, 3003, 2849, 2797, 1662, 1631, 1573, 1534, 1475, 1404, 1341, 1312, 1274, 1151, 1094, 1031, 899, 836, 812, 757, 724, 646, 621, 570 cm^−1^; Elemental analysis (%) calcd for: C_2_H_3_N_7_O_4_ (189.09): C, 12.70; H, 1.60; N, 51.85; found: C, 12.54; H, 1.56; N, 50.02.

### Hydroxylammonium N-(5-amino-3-nitro-1H-1,2,4-triazol-1-yl)nitramidate (HHF-T)

A solution of NT (189 mg, 1 mmol) in methanol (5 mL) was stirred at room temperature until all the solids were dissolved, and hydroxylamine (50 wt% in water, 0.06 ml, 1 mmol) was added. After stirring at room temperature for 30 min, methanol was removed by blowing air and the residue was dried in vacuo to yield HHF-T (56%). ^1^H NMR (400 MHz, DMSO-d_6_) δ 9.23 (br, 4H), 6.53 (s, 2H); ^13^C NMR (125 MHz, DMSO) δ 156.4, 153.3; ^15^N NMR (D_6_-DMSO): δ −3.73, −26.83, −109.41, −122.82, −175.31, −186.78, −291.93, −329.22; IR (KBr pellet): υ 3442, 3369, 3303, 3259, 3168, 3149, 3135, 2723, 1654, 1547, 1516, 1425, 1360, 1240, 1196, 1150, 1072, 1010, 910, 838, 760, 726, 714, 618 cm^−1^; Elemental analysis (%) calcd for: C_2_H_6_N_8_O_5_ (222.12): C, 10.81; H, 2.72; N, 50.45; found: C, 10.43; H, 2.86; N, 49.48.

### Hydroxylammonium 3-amino-4-nitramino-furazan (HHF-F)

To a solution of diaminofurazan (10 mmol, 1.00 g) in conc. H_2_SO_4_ (20 mL), ammonium nitrate (10 mmol, 0.80 g) was added at −15 °C. The reaction was stirred at −15 °C for 2 h. The final transparent solution was then quenched by ice (ca. 100 g) and the aqueous layer was extracted with ethyl ether (3 × 50 mL). To the combined organic layers, aqueous hydroxylamine (50 wt%. in water, 10 mmol, 660 mg) was added. The precipitate was collected by filtration and was then dried in vacuo to give HHF-F (1.26 g, 71% yield). ^1^H NMR (400 MHz, DMSO-d_6_) δ 10.11 (s, 4H), 5.42 (s, 2H). ^13^C NMR (101 MHz, DMSO) δ 153.5, 152.1. IR (KBr pellet): υ 3361, 3293, 3055, 2693, 1611, 1505, 1306, 1170, 1035, 1005, 908, 852, 762, 741, 714, 701, 537, 460 cm^−1^; Elemental analysis (%) calcd for: C_2_H_6_N_6_O_4_ (178.0451): C, 13.49; H, 3.40; N, 47.19; found: C, 13.33; H, 3.47; N, 47.26. DSC: T_d_ 181 °C.

### 3-Amino-4-nitramino-furazan (NF)

Compound HHF-F (10 mmol, 1.62 g) was added to ice-cold 5% H_2_SO_4_ and the aqueous mixture was extracted by ethyl ether (3 × 25 mL). The combine extracts were concentrated by rotary evaporation to give NF (1.32 g, 91%). ^1^H NMR (400 MHz, DMSO-d_6_) δ 11.58 (s, 1H), 5.02 (s, 2H). ^13^C NMR (101 MHz, DMSO) δ 154.3, 142.8. IR (KBr pellet): υ 3460, 3347, 1617, 1547, 1469, 1398, 1346, 1272, 1027, 918, 895, 845, 776, 754, 706, 649, 567, 502, 445 cm^−1^; Elemental analysis (%) calcd for: C_2_H_3_N_5_O_3_ (145.0236): C, 16.56; H, 2.08; N, 48.27; found: C, 16.49; H, 2.06; N, 48.28. DSC: T_d_ 126 °C.

### Hydroxylammonium (4-methyl-1,2,5-oxadiazol-3-yl)nitramidate (HHF-N4)

To a solution of 4-methyl-1,2,5-oxadiazol-3-amine (10 mmol, 0.99 g) in conc. H_2_SO_4_ (20 mL), ammonium nitrate (20 mmol, 1.60 g) was added at −10 °C. The reaction was stirred vigorously at −10 °C until a transparent solution was obtained. The resulting reaction was then quenched by ice (ca. 100 g) and the aqueous layer was extracted with ethyl ether (3 × 50 mL). The combined organic layers were dried over Na_2_SO_4_ and the solvent was removed by rotary evaporation to give N-(4-methyl-1,2,5-oxadiazol-3-yl)nitramide. A solution of N-(4-methyl-1,2,5-oxadiazol-3-yl)nitramide (144 mg, 1 mmol) in methanol (5 mL) was stirred at room temperature until all the solids were dissolved, and hydroxylamine (50 wt% in water, 0.06 ml, 1 mmol) was added. After stirring at room temperature for 30 min, methanol was removed by blowing air and the residue was dried in vacuo to yield HHF-N4 (71%). ^1^H NMR (400 MHz, DMSO-d_6_) δ 10.06 (s, 4H), 2.13 (s, 3H); ^13^C NMR (125 MHz, DMSO) δ 158.44, 149.08, 8.66; Elemental analysis (%) calcd for: C_3_H_7_N_5_O_4_ (177.05): C, 20.34; H, 3.98; N, 39.54; found: C, 20.46; H, 3.81; N, 39.81.

### Hydroxylammonium (5-carbamoyl-2H-1,2,3-triazol-4-yl)nitramidate (HHF-N14)

To a solution of 5-amino-2H-1,2,3-triazole-4-carboxamide (10 mmol, 1.27 g) in conc. H_2_SO_4_ (20 mL), ammonium nitrate (20 mmol, 1.60 g) was added at −10 °C. The reaction was stirred vigorously at −10 °C until a transparent solution was obtained. The resulting reaction was then quenched by ice (ca. 100 g) and the aqueous layer was extracted with ethyl ether (3 × 50 mL). The combined organic layers were dried over Na_2_SO_4_ and the solvent was removed by rotary evaporation to give 5-(nitroamino)-2H-1,2,3-triazole-4-carboxamide. A solution of 5-(nitroamino)-2H-1,2,3-triazole-4-carboxamide (172 mg, 1 mmol) in methanol (5 mL) was stirred at room temperature until all the solids were dissolved, and hydroxylamine (50 wt% in water, 0.06 ml, 1 mmol) was added. After stirring at room temperature for 30 min, methanol was removed by blowing air and the residue was dried in vacuo to yield HHF-N14 (63%). ^1^H NMR (400 MHz, DMSO-d_6_) δ 10.33 (s, 4H), 7.67 (s, 1H), 7.37 (s, 1H); ^13^C NMR (125 MHz, DMSO) δ 163.0, 142.8, 127.0; Elemental analysis (%) calcd for: C_3_H_7_N_7_O_4_ (205.06): C, 17.57; H, 3.44; N, 47.80; found: C, 17.73; H, 3.26; N, 47.72.

#### General Synthesis procedure of HHF-H

Reactant(1 mmol,1eq) containing acidic protons was dissolved in methyl alcohol(5 mL), then aqueous hydroxylamine (50 wt% in water, 1 mmol, 1eq) was added dropwise. After stirring for 12 h in room temperature, the solvent was removed and crystalline solid was collected by filtration to give corresponding hydroxylammonium salt.

## Supplementary information

Supplementary Information

## Data Availability

Data that support the finding of this study are available from the corresponding authors on request. X-ray coordinates from the crystal structure determination have been deposited with the Cambridge Crystallographic Data Centre (CCDC), under deposition number (NT, 2102199; HHF-T, 2102200; NF, 2094882; and HHF-F, 2094884; HHF-N14, 2201536; HHF-N4, 2201537; HHF-H4, 2201538; HHF-H11, 2201539; HHF-H17, 2201540). These data can be obtained free of charge from The Cambridge Crystallographic Data Centre via www.ccdc.cam.ac.uk/data_request/cif. All relevant data were included within this article and Supplementary Information.
