# Supplementary material for: Size-matched hydrogen bonded hydroxylammonium frameworks for regulation of energetic materials
Source: Nat Commun. 2022 Nov 14;13:6937. doi: 10.1038/s41467-022-34686-8 (PMC9663426; doi:10.1038/s41467-022-34686-8)
Supplement: Supplementary file 1 — Supplementary Information [file 41467_2022_34686_MOESM1_ESM.pdf]

Supplementary Information

Size-Matched Hydrogen Bonded Hydroxylammonium Frameworks for Regulation of Energetic Materials

Qi Lai,‡[1,2,3] Le Pei,‡[1,2,3] Teng Fei,[1] Ping Yin* [1,2,3], Siping Pang*[1], Jean'ne M. Shreeve*[2]

[1] School of Materials Science & Engineering, Beijing Institute of Technology, Beijing 100081, China

[2] Department of Chemistry, University of Idaho, Moscow, ID 83844-2343, United States

[3] Beijing Institute of Technology Chongqing Innovation Center, Chongqing, 401120, China

*E-mail: pingyin@bit.edu.cn, pangsp@bit.edu.cn, jshreeve@uidaho.edu

‡ These authors contributed equally to this work

I. ORTEP Drawings of the Crystal Structures ….……….…….………S2

II. NMR Spectra Data …………………..…………….…………………S19

III. DSC Plots ……………………………………………………….......S25

IV. Computation and Properties…………………….……..………...…S28

V. Application expansion of size matching strategy…………………S39

VI. Supplementary References……………………….…….…..………S52

I. ORTEP Drawings of the Crystal Structure

1. Crystal data of compound NT

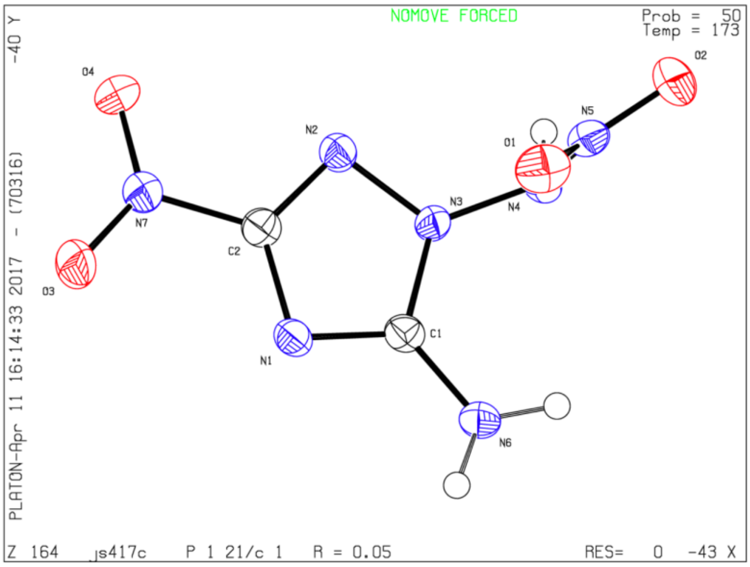

Supplementary Fig. 1. Asymmetric unit of js417c. Anisotropic displacement parameters are drawn at 50% probability. CCDC: 2102199

Supplementary Tab.1. Crystal data and structure refinement for NT.

Identification code	js417c
Empirical formula	$C_2H_3N_7O_4$
Formula weight	189.11
Temperature/K	173
Space group	P21/n
a/Å	6.4149(3)
b/Å	10.3449(5)
c/Å	10.7002(5)
α/°	90
β/°	98.909(4)
γ/°	90
Volume/Å^3	701.51(6)
Z	4
ρ_{calc}g/cm^3	1.791
μ/mm^{-1}	1.487
F(000)	384.0

2. Crystal data of HHF-T

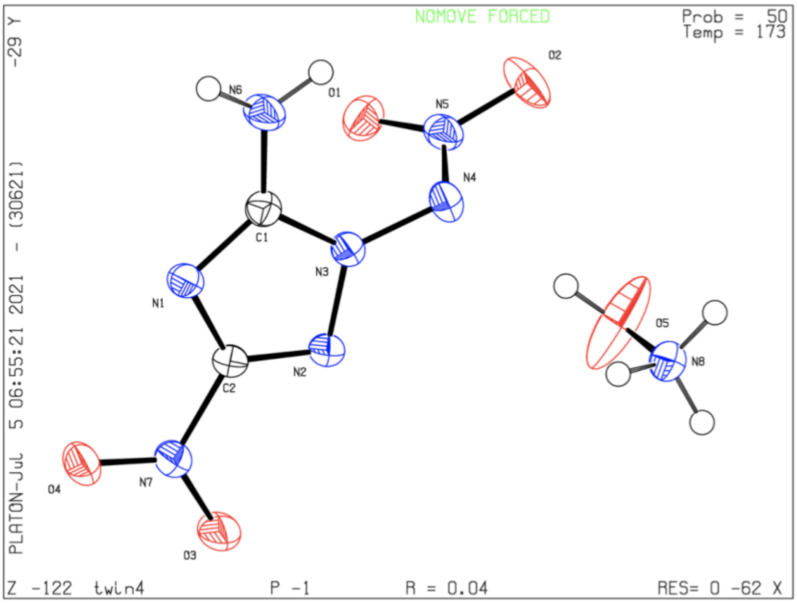

Supplementary Fig. 2. Asymmetric unit of twin4. Anisotropic displacement parameters are drawn at 50% probability. CCDC: 2102200

Supplementary Tab. 2. Crystal data and structure refinement for HHF-T.

Identification code	twin4
Empirical formula	$C_2H_2N_7O_4$, H_4NO
Formula weight	222.15
Temperature/K	173
Space group	P-1
a/Å	7.640(2)
b/Å	7.719(2)
c/Å	8.094(2)
α/°	78.312(3)
β/°	65.723(4)
γ/°	69.745(3)
Volume/Å^3	407.20(2)
Z	2
ρ_{calc}g/cm^3	1.812
μ/mm^{-1}	0.170
F(000)	228.0

3. Crystal data of compound NF

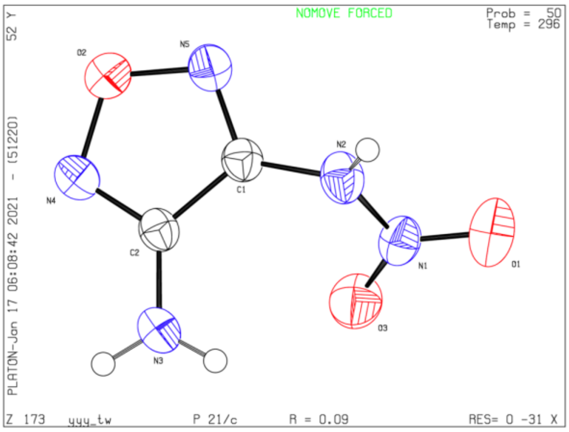

Supplementary Fig. 3. Asymmetric unit of yyy_tw. Anisotropic displacement parameters are drawn at 50% probability. CCDC: 2094882

Supplementary Tab. 3. Crystal data and structure refinement for NF.

Identification code	yyy_tw
Empirical formula	$C_2H_3N_5O_3$
Formula weight	145.09
Temperature/K	296
Space group	P21/c
a/Å	8.483(12)
b/Å	6.177(8)
c/Å	11.249(13)
α/°	90
β/°	109.716(9)
γ/°	90
Volume/Å^3	555.0(12)
Z	4
ρ_{calc}g/cm^3	1.737
μ/mm^{-1}	0.159
F(000)	296.0

4. Crystal data of compound HHF-F

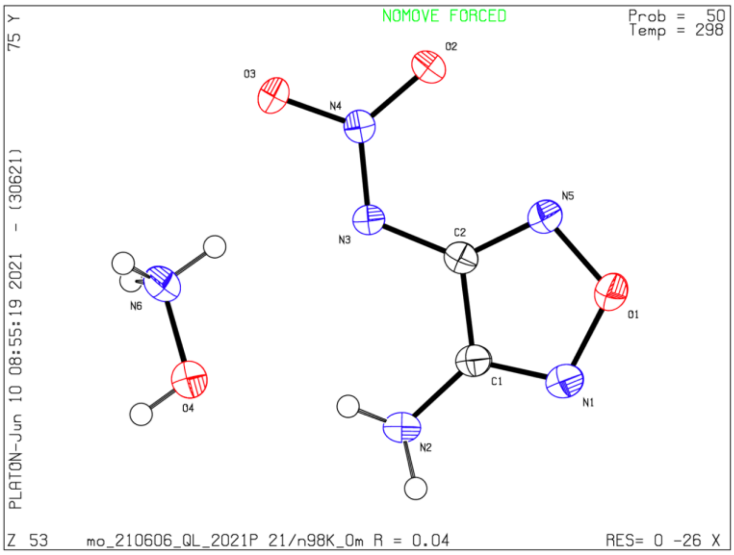

Supplementary Fig. 4. Asymmetric unit of mo_210606_QL_20210529_298K_0m. Anisotropic displacement parameters are drawn at 50% probability. CCDC: 2094484

Supplementary Tab. 4. Crystal data and structure refinement for HHF-F.

Identification code	mo_210606_QL_20210529_298K_0m
Empirical formula	$C_2H_2N_5O_3$, H_4NO
Formula weight	178.13
Temperature/K	298
Space group	P21/n
a/Å	6.589(10)
b/Å	12.545(18)
c/Å	8.200(13)
α/°	90
β/°	106.280(1)
γ/°	90
Volume/Å^3	650.6(17)
Z	4
ρ_{calc}g/cm^3	1.819
μ/mm^{-1}	0.169
F(000)	368.0

5. Crystal data of compound HHF-N14

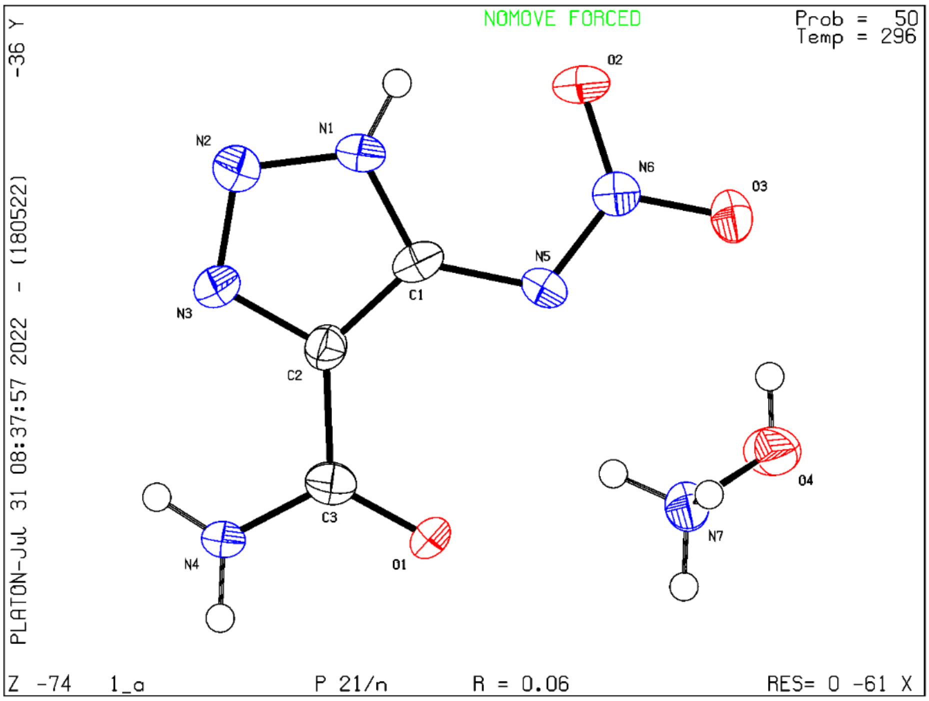

Supplementary Fig. 5. Asymmetric unit of 1-a. Anisotropic displacement parameters are drawn at 50% probability. CCDC: 2201536

Supplementary Tab. 5. Crystal data and structure refinement for HHF-N14.

Identification code	1-a
Empirical formula	$C_3H_7N_7O_4$
Formula weight	205.16
Temperature/K	296
Space group	P21/n
a/Å	5.1960(6)
b/Å	4.8598(5)
c/Å	29.794(3)
α/°	90
β/°	93.102(3)
γ/°	90
Volume/Å^3	751.24(14)
Z	4
ρ_{calc}g/cm^3	1.814
μ/mm^{-1}	0.163
F(000)	424.0

6. Crystal data of compound HHF-N4

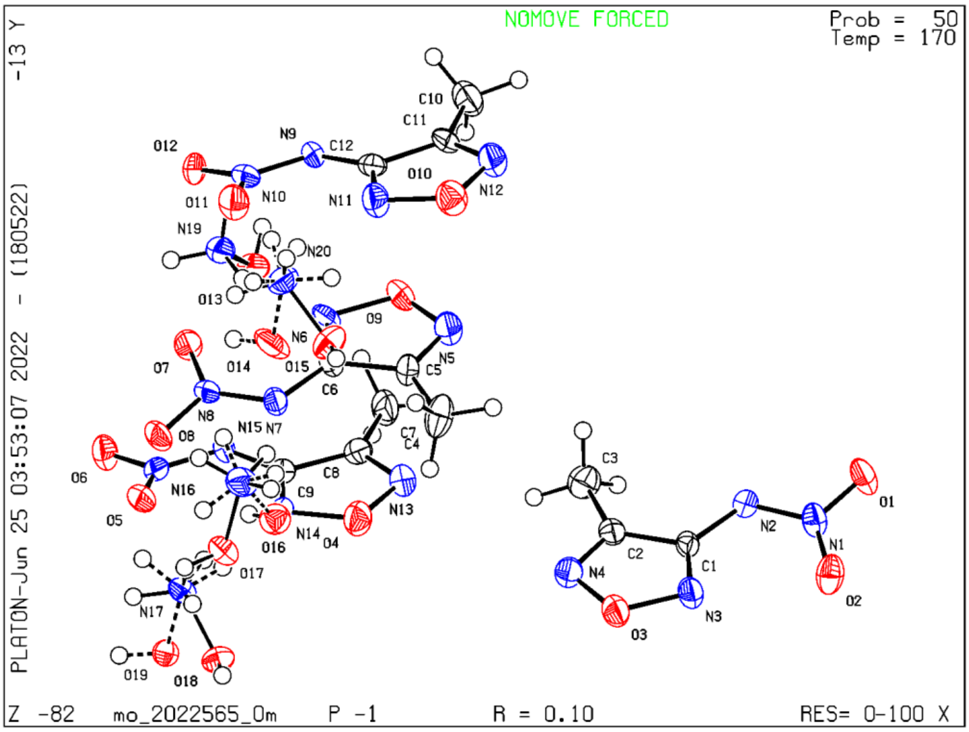

Supplementary Fig. 6. Asymmetric unit of mo_2022565_0m. Anisotropic displacement parameters are drawn at 50% probability. CCDC: 2201537

Supplementary Tab. 6. Crystal data and structure refinement for HHF-N4.

Identification code	mo_2022565_0m
Empirical formula	$C_3H_7N_5O_4$
Formula weight	145.09
Temperature/K	170
Space group	P-1
a/Å	10.320(3)
b/Å	10.354(2)
c/Å	13.616(3)
α/°	92.100(8)
β/°	91.385(8)
γ/°	94.516(8)
Volume/Å^3	1448.9(6)
Z	8
ρ_{calc}g/cm^3	1.624
μ/mm^{-1}	0.147
F(000)	736.0

7. Crystal data of compound HHF-H4

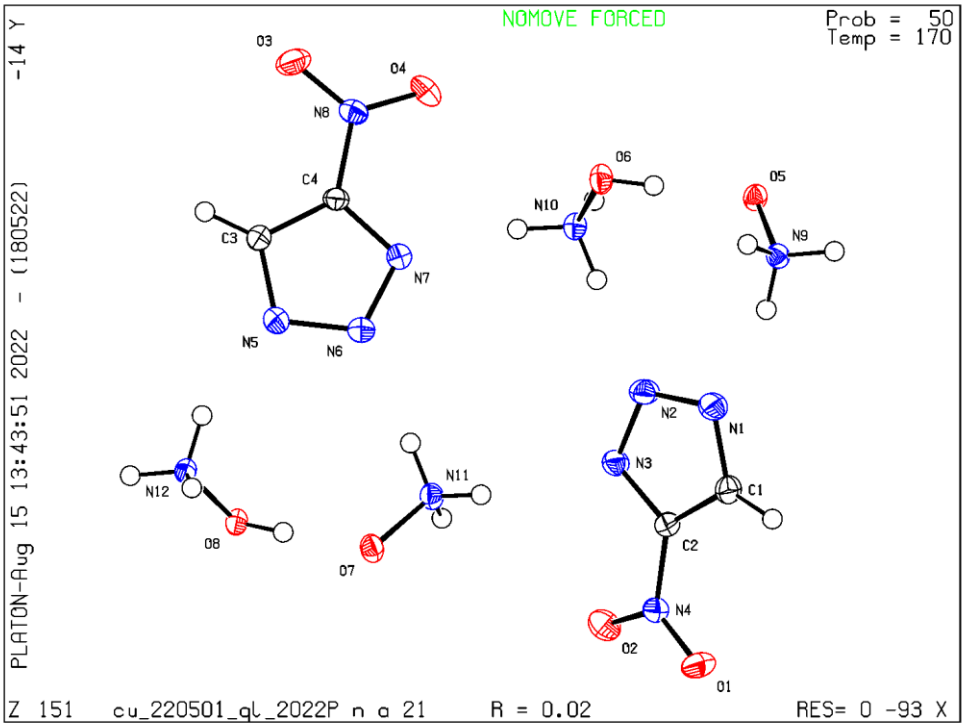

Supplementary Fig. 7. Asymmetric unit of cu_220501_QL_20220428_6. Anisotropic displacement parameters are drawn at 50% probability. CCDC: 2201538

Supplementary Tab. 7. Crystal data and structure refinement for HHF-H4.

Identification code	cu_220501_QL_20220428_6
Empirical formula	$C_2H_8N_6O_4$
Formula weight	180.14
Temperature/K	170
Space group	$Pna2_1$
a/Å	24.7340(12)
b/Å	3.5707(2)
c/Å	3.5707(2)
α/°	90
β/°	90
γ/°	90
Volume/Å^3	3.5707(2)
Z	8
ρ_{calc}g/cm^3	1.645
μ/mm^{-1}	1.341
F(000)	752.0

8. Crystal data of compound HHF-H11

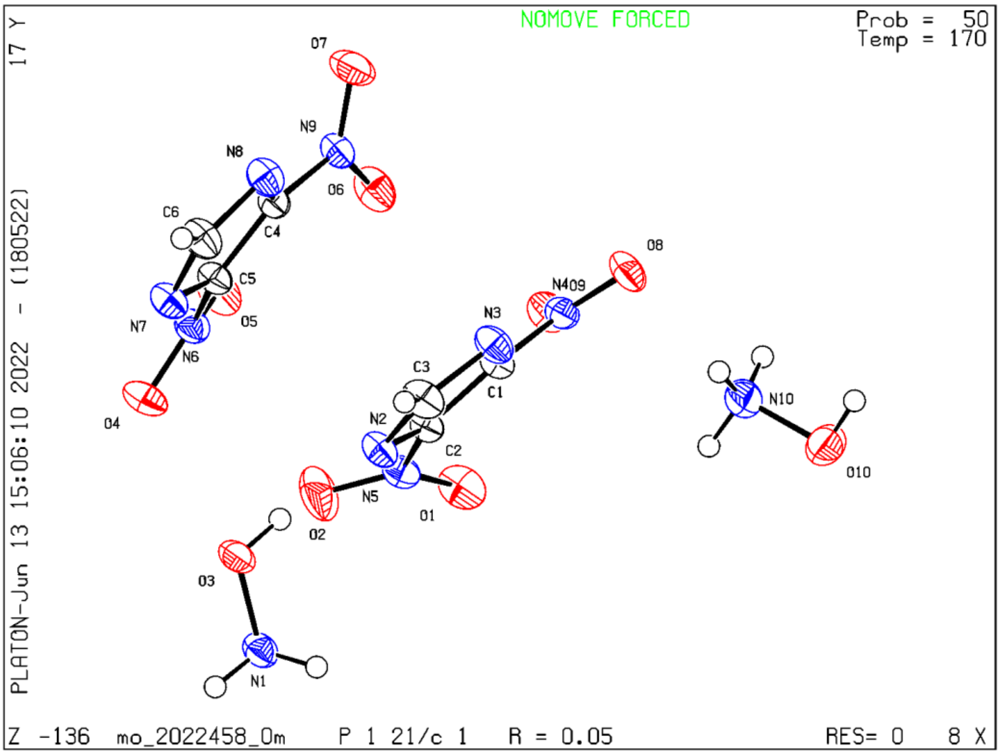

Supplementary Fig. 8. Asymmetric unit ofmo_2022458_0m. Anisotropic displacement parameters are drawn at 50% probability. CCDC: 2201539

Supplementary Tab. 8. Crystal data and structure refinement for HHF-H11.

Identification code	mo_2022458_0m
Empirical formula	$C_3H_5N_5O_5$
Formula weight	191.12
Temperature/K	170
Space group	P 21/c
a/Å	3.801(6)
b/Å	24.39(5)
c/Å	9.309(14)
α/°	90
β/°	91.038(3)
γ/°	90
Volume/Å^3	1422.41(9)
Z	8
ρ_{calc}g/cm^3	1.785
μ/mm^{-1}	0.168
F(000)	784.0

9. Crystal data of compound HHF-H17

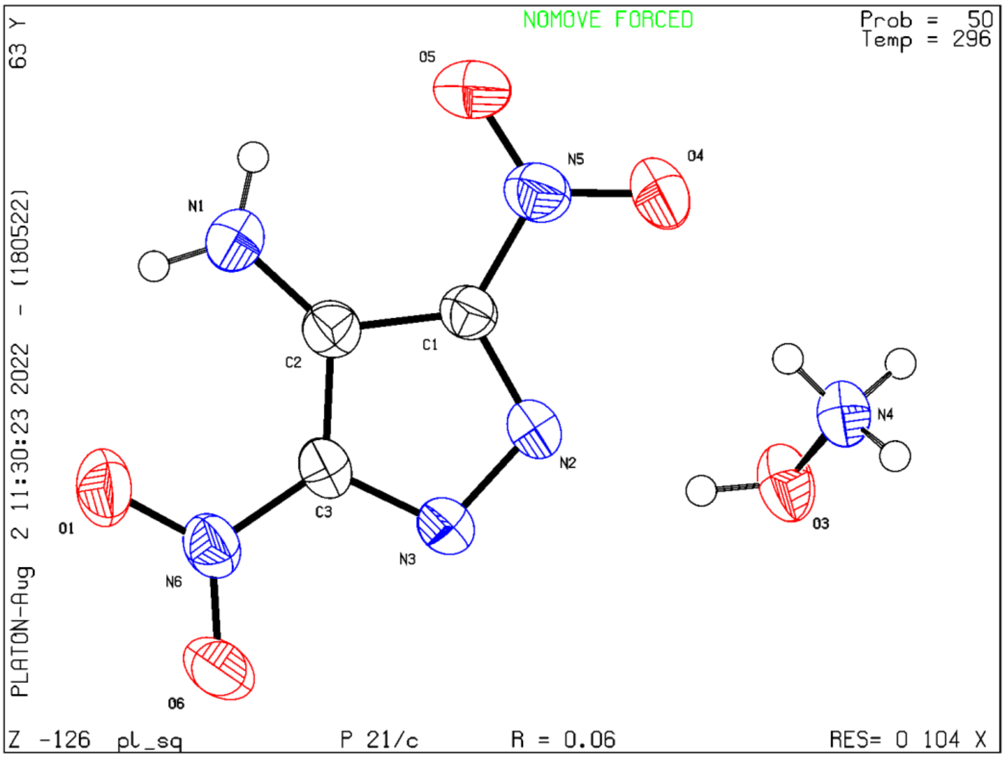

Supplementary Fig. 9. Asymmetric unit of pl_sq. Anisotropic displacement parameters are drawn at 50% probability. CCDC: 2201540

Supplementary Tab. 9. Crystal data and structure refinement for HHF-H17.

Identification code	pl_sq
Empirical formula	$C_3H_6N_6O_5$
Formula weight	206.14
Temperature/K	296
Space group	P 21/c
a/Å	3.801(6)
b/Å	24.39(5)
c/Å	9.309(14)
α/°	90
β/°	96.726(15)
γ/°	90
Volume/Å^3	857(2)
Z	4
ρ_{calc}g/cm^3	1.598
μ/mm^{-1}	0.149
F(000)	424.0

10. Crystal data of N-1

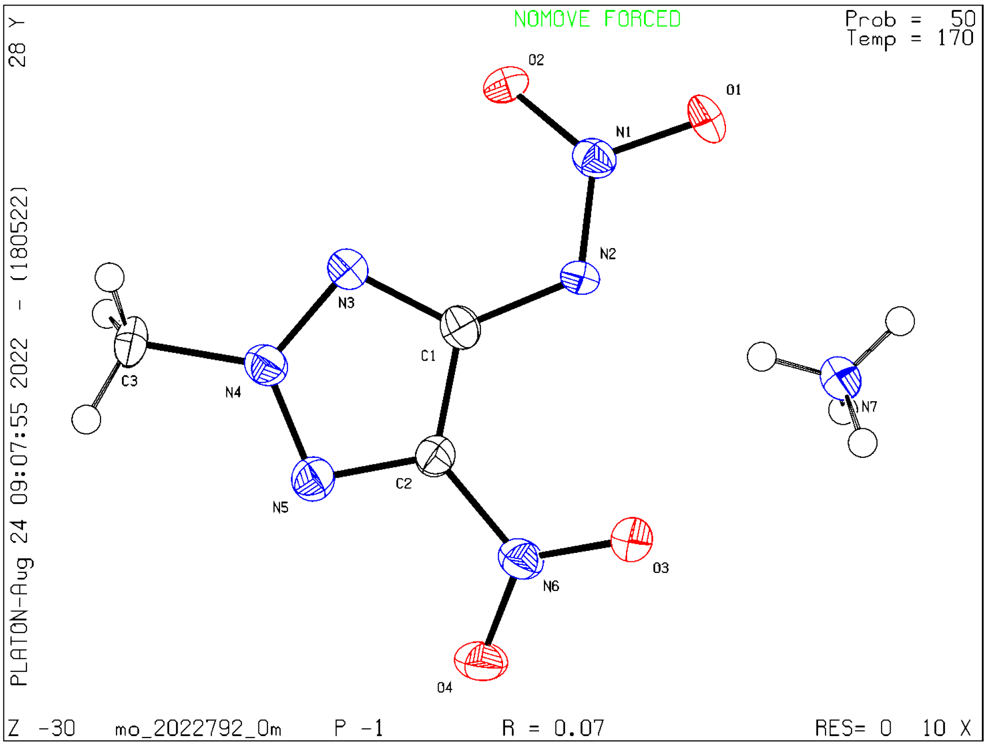

Supplementary Fig. 10. Asymmetric unit of mo_2022792_0m. Anisotropic displacement parameters are drawn at 50% probability. CCDC: 2203242

Supplementary Tab. 10. Crystal data and structure refinement for N-1.

Identification code	mo_2022792_0m
Empirical formula	$C_3H_7N_7O_4$
Formula weight	205.16
Temperature/K	170
Space group	P-1
a/Å	6.695(4)
b/Å	7.286(6)
c/Å	8.487(7)
α/°	99.79(2)
β/°	102.865(1)
γ/°	99.038(18)
Volume/Å^3	298.85(2)
Z	2
ρ_{calc}g/cm^3	1.748
μ/mm^{-1}	0.157
F(000)	212.0

11. Crystal data of N-2

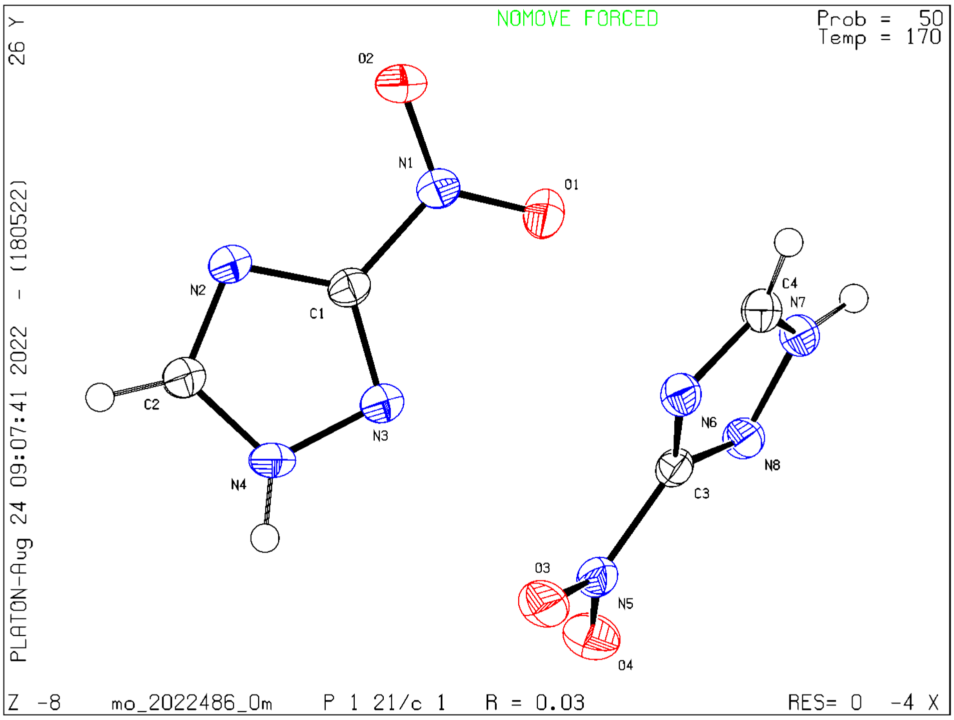

Supplementary Fig. 11. Asymmetric unit of mo_2022486_0m. Anisotropic displacement parameters are drawn at 50% probability. CCDC: 2203236

Supplementary Tab. 11. Crystal data and structure refinement for N-2.

Identification code	mo_2022486_0m
Empirical formula	$C_2H_2N_4O_2$
Formula weight	114.08
Temperature/K	170
Space group	P21/c
a/Å	8.8973(6)
b/Å	10.0597(6)
c/Å	9.9665(6)
α/°	90
β/°	106.828(2)
γ/°	90
Volume/Å^3	853.84(9)
Z	8
ρ_{calc}g/cm^3	1.775
μ/mm^{-1}	0.157
F(000)	464.0

12. Crystal data of N-3

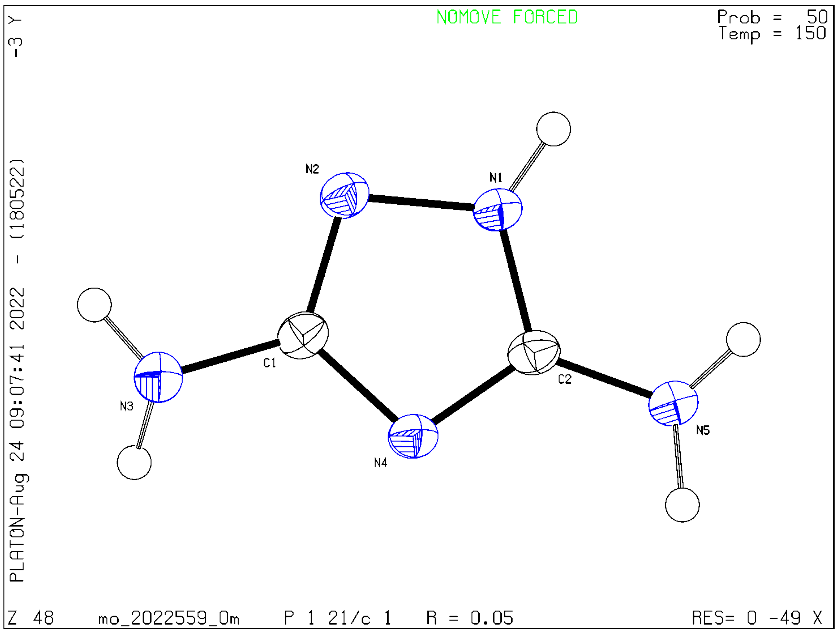

Supplementary Fig. 12. Asymmetric unit of mo_2022559_0m. Anisotropic displacement parameters are drawn at 50% probability. CCDC: 2203237

Supplementary Tab. 12. Crystal data and structure refinement for N-3.

Identification code	mo_2022559_0m
Empirical formula	$C_2H_5N_5$
Formula weight	99.11
Temperature/K	150
Space group	P21/c
a/Å	10.6320(12)
b/Å	4.2922(6)
c/Å	10.8180(6)
α/°	90
β/°	118.755(4)
γ/°	90
Volume/Å^3	432.80(9)
Z	4
ρ_{calc}g/cm^3	1.521
μ/mm^{-1}	0.115
F(000)	208.0

13. Crystal data of N-4

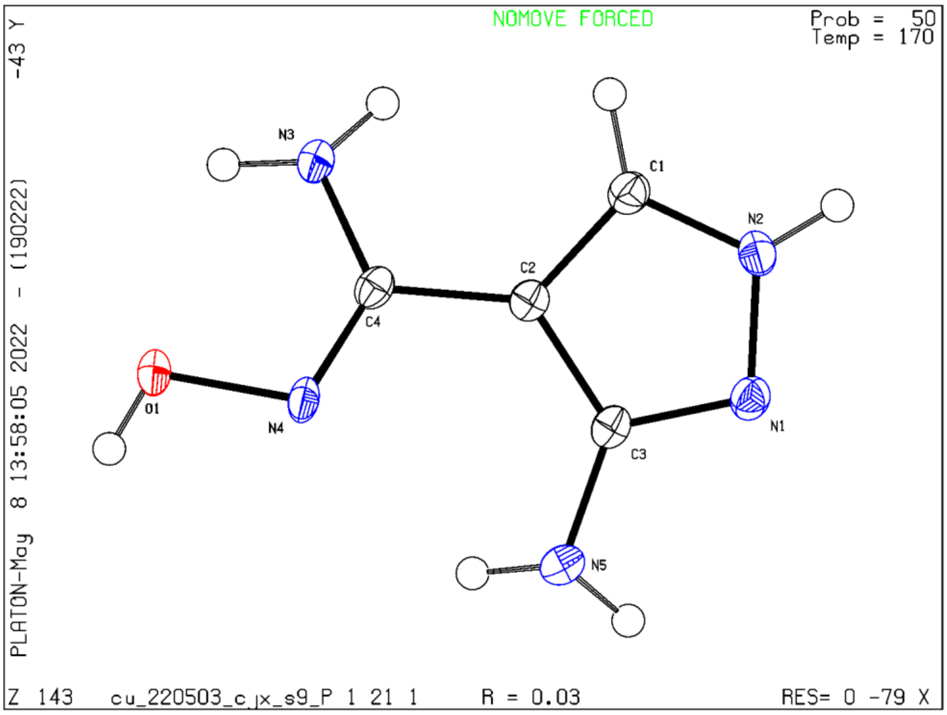

Supplementary Fig. 13. Asymmetric unit of cu_220503_cjx_s9_3no2_0m. Anisotropic displacement parameters are drawn at 50% probability. CCDC: 2201541

Supplementary Tab. 13. Crystal data and structure refinement for N-4.

Identification code	cu_220503_cjx_s9_3no2_0m
Empirical formula	$C_4H_7N_5O$
Formula weight	141.15
Temperature/K	170
Space group	P21
a/Å	4.8345(2)
b/Å	8.9774(4)
c/Å	7.0631(3)
α/°	90
β/°	102.865(1)
γ/°	90
Volume/Å^3	298.85(2)
Z	2
ρ_{calc}g/cm^3	1.569
μ/mm^{-1}	1.026
F(000)	148.0

14. Crystal data of N-5

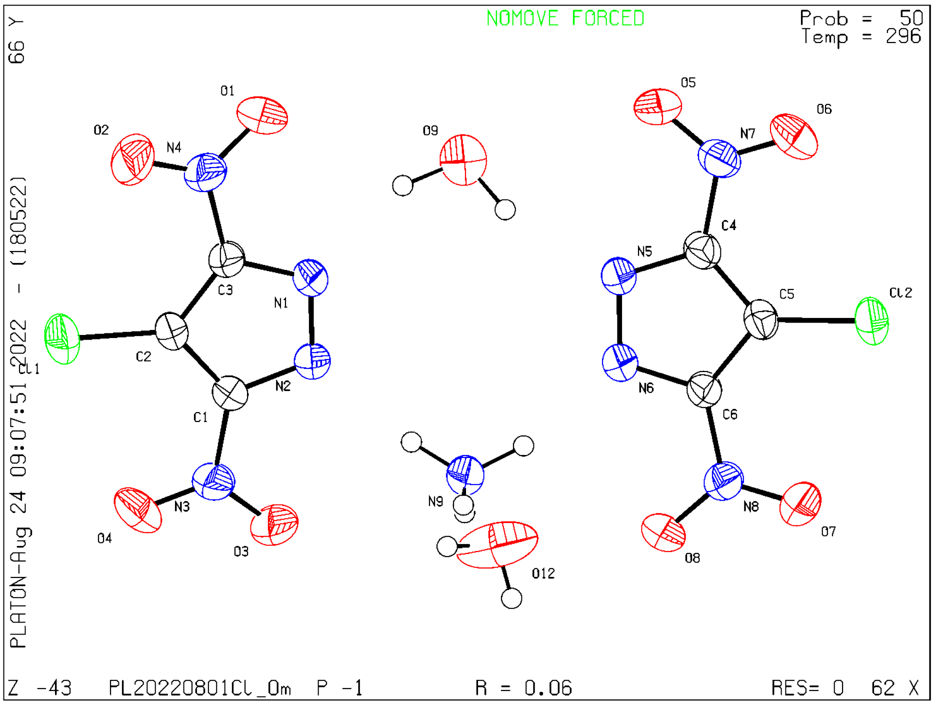

Supplementary Fig. 14. Asymmetric unit of PL20220801Cl_0m. Anisotropic displacement parameters are drawn at 50% probability. CCDC: 2203240

Supplementary Tab. 14. Crystal data and structure refinement for N-5.

Identification code	mo_2022792_0m
Empirical formula	$2(C_3ClN_4O_4)$, $2(H_2O)$, H_4N
Formula weight	437.11
Temperature/K	296
Space group	P-1
a/Å	7.668(7)
b/Å	8.115(8)
c/Å	14.459(14)
α/°	91.573(14)
β/°	102.948(15)
γ/°	108.177(13)
Volume/Å^3	298.85(2)
Z	2
ρ_{calc}g/cm^3	1.748
μ/mm^{-1}	0.466
F(000)	442.0

15. Crystal data of N-6

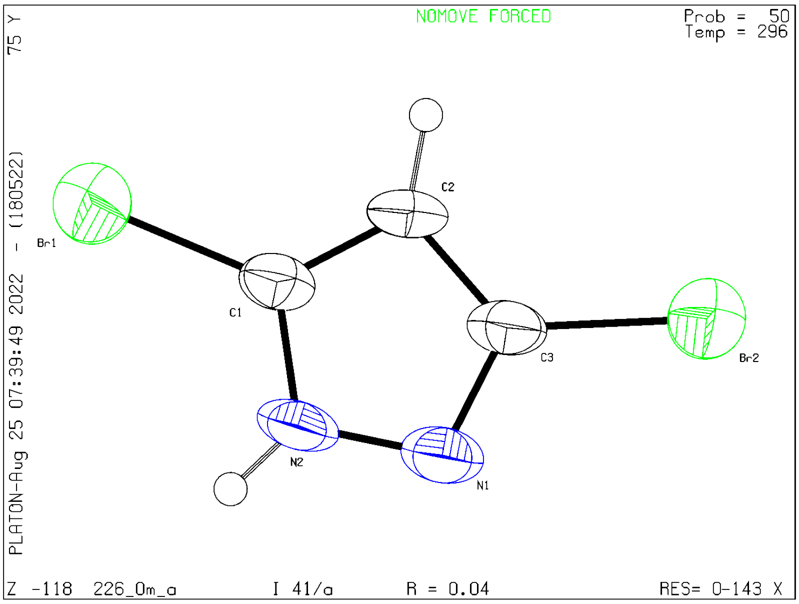

Supplementary Fig. 15. Asymmetric unit of 226_0m_a. Anisotropic displacement parameters are drawn at 50% probability. CCDC: 2203238

**Supplementary Tab. 15.** Crystal data and structure refinement for NT-6.	
Identification code	226_0m_a
Empirical formula	$C_3H_2N_2Br_2$
Formula weight	225.87
Temperature/K	296
Space group	I41/a
a/Å	15.730(6)
b/Å	15.730(6)
c/Å	9.663(4)
α/°	90
β/°	90
γ/°	90
Volume/Å^3	2391(2)
Z	16
ρ_{calc}g/cm^3	2.510
μ/mm^{-1}	13.434
F(000)	1664.0

16. Crystal data of **N-7**

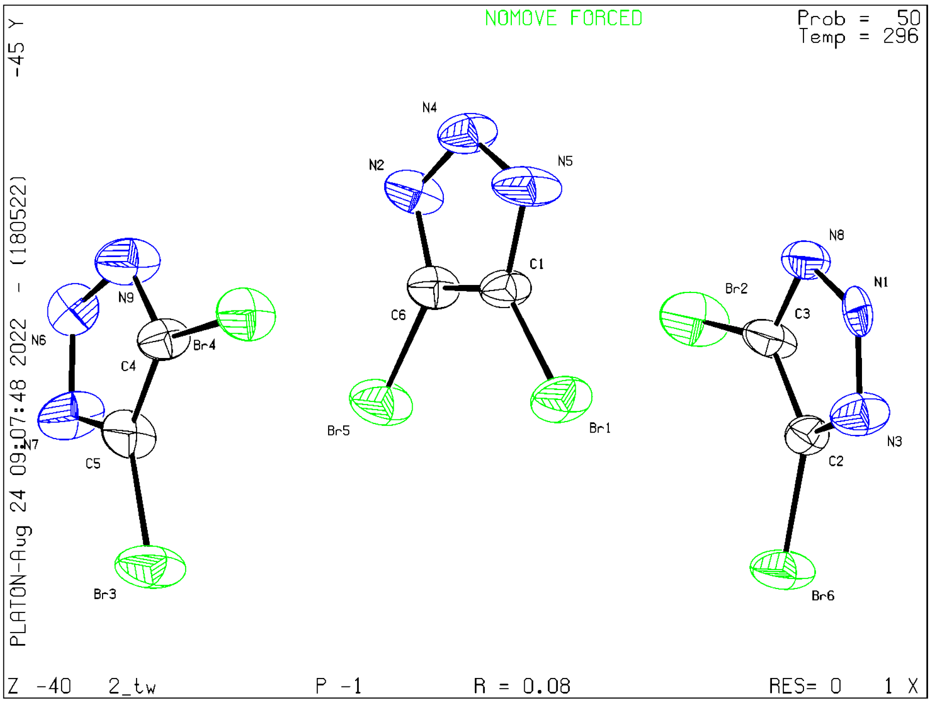

Supplementary Fig. 16. Asymmetric unit of 2_tw. Anisotropic displacement parameters are drawn at 50% probability. CCDC: 2203239

**Supplementary Tab. 16.** Crystal data and structure refinement for NT-7.	
Identification code	2_tw
Empirical formula	$C_3H_2N_2Br_2$
Formula weight	225.87
Temperature/K	296
Space group	P-1
a/Å	7.9127(14)
b/Å	10.4942(15)
c/Å	11.8451(14)
α/°	112.250(11)
β/°	104.401(11)
γ/°	101.103(15)
Volume/Å^3	835.4(3)
Z	16
ρ_{calc}g/cm^3	2.510
μ/mm^{-1}	14.423
F(000)	618.0

17. Crystal data of **N-8**

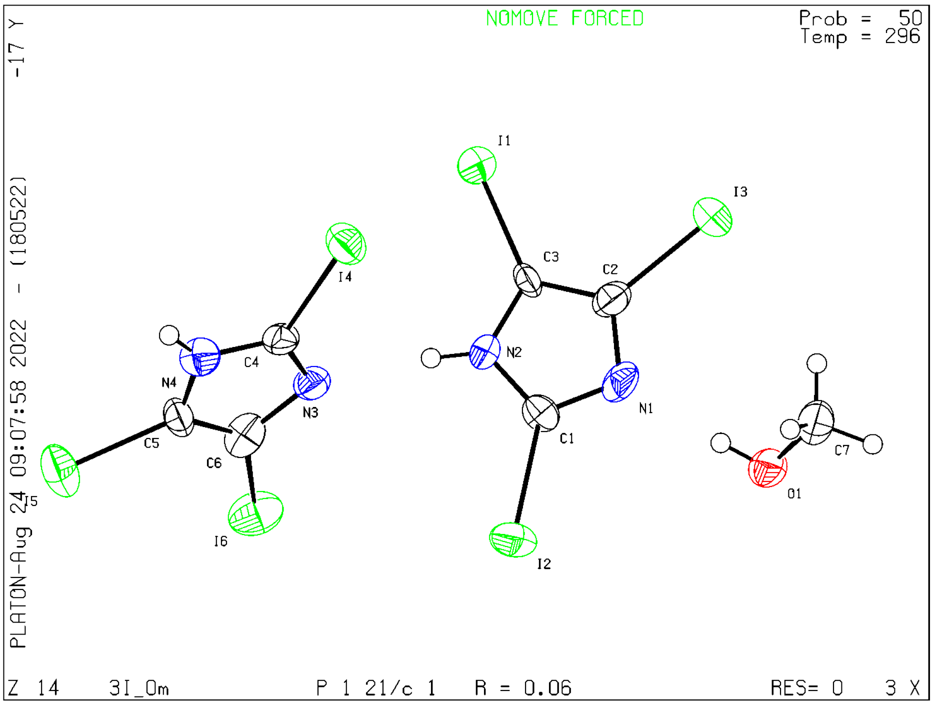

Supplementary Fig. 17. Asymmetric unit of 3I_0m. Anisotropic displacement parameters are drawn at 50% probability. CCDC: 2203241

**Supplementary Tab. 17.** Crystal data and structure refinement for NT-8.	
Identification code	3I_0m
Empirical formula	$2(C_3HN_2I_3)$, CH_4O
Formula weight	923.56
Temperature/K	296
Space group	P21/c
a/Å	11.354(15)
b/Å	9.119(12)
c/Å	18.04(2)
α/°	90
β/°	94.910(18)
γ/°	90
Volume/Å^3	1861(4)
Z	4
ρ_{calc}g/cm^3	3.296
μ/mm^{-1}	10.011
F(000)	1608.0

II. NMR Spectra Data

Supplementary Fig. 18. ^{1}H NMR of compound NT

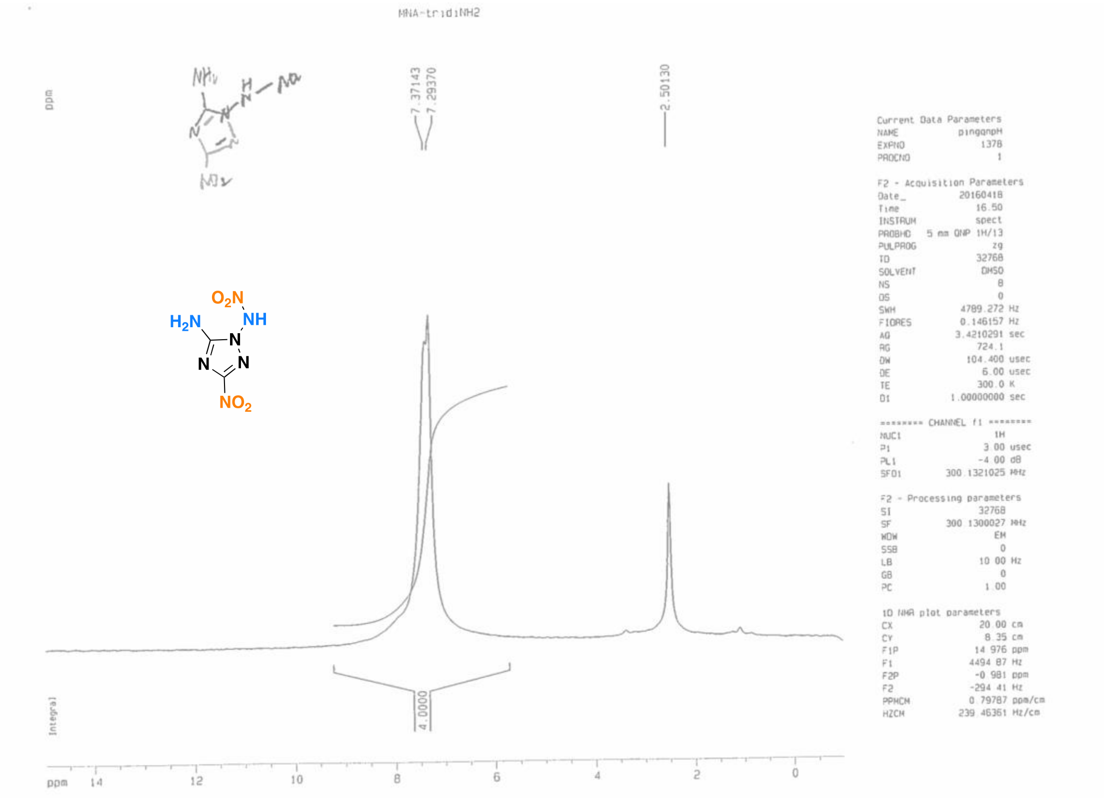

Supplementary Fig. 19. ^{13}C NMR of compound NT

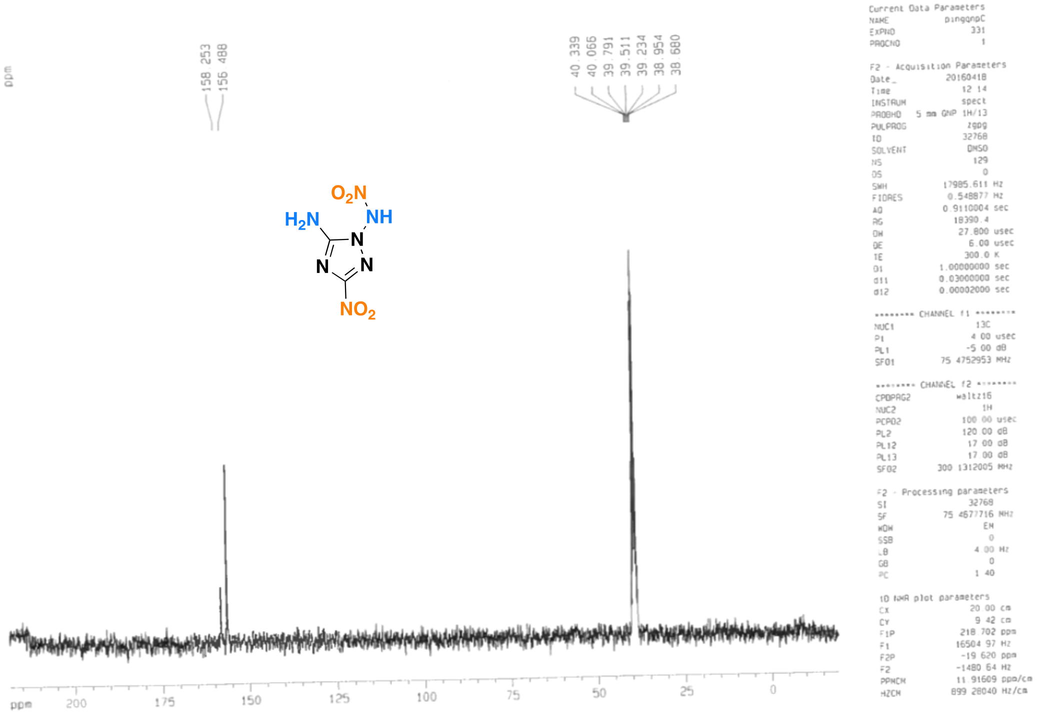

Supplementary Fig. 20. ^{1}H NMR of compound HHF-T

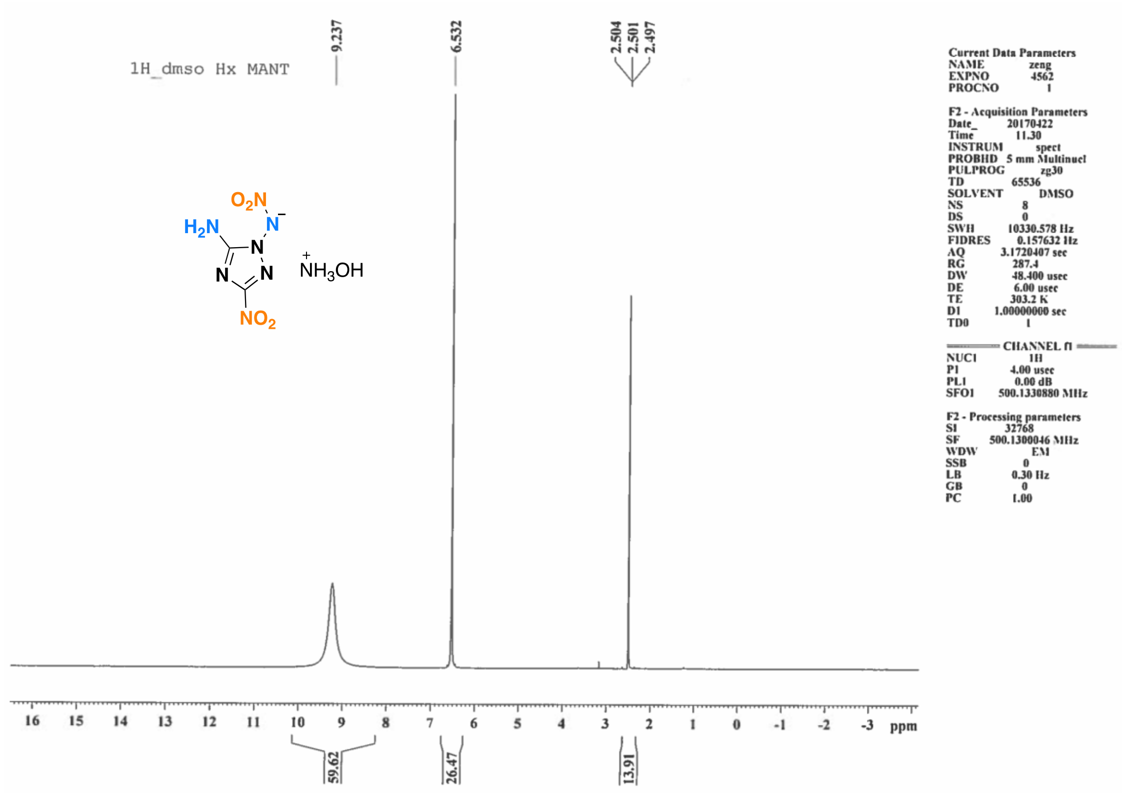

Supplementary Fig. 21. ^{13}C NMR of compound HHF-T

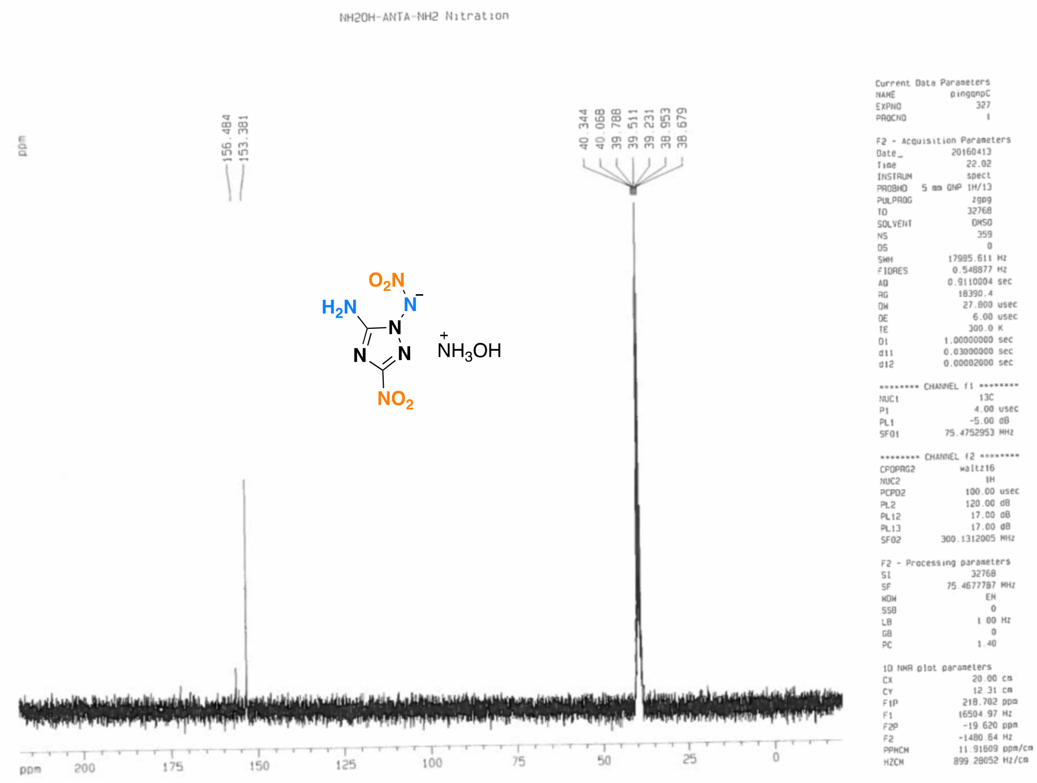

Supplementary Fig. 22. ^{1}H NMR of compound NF

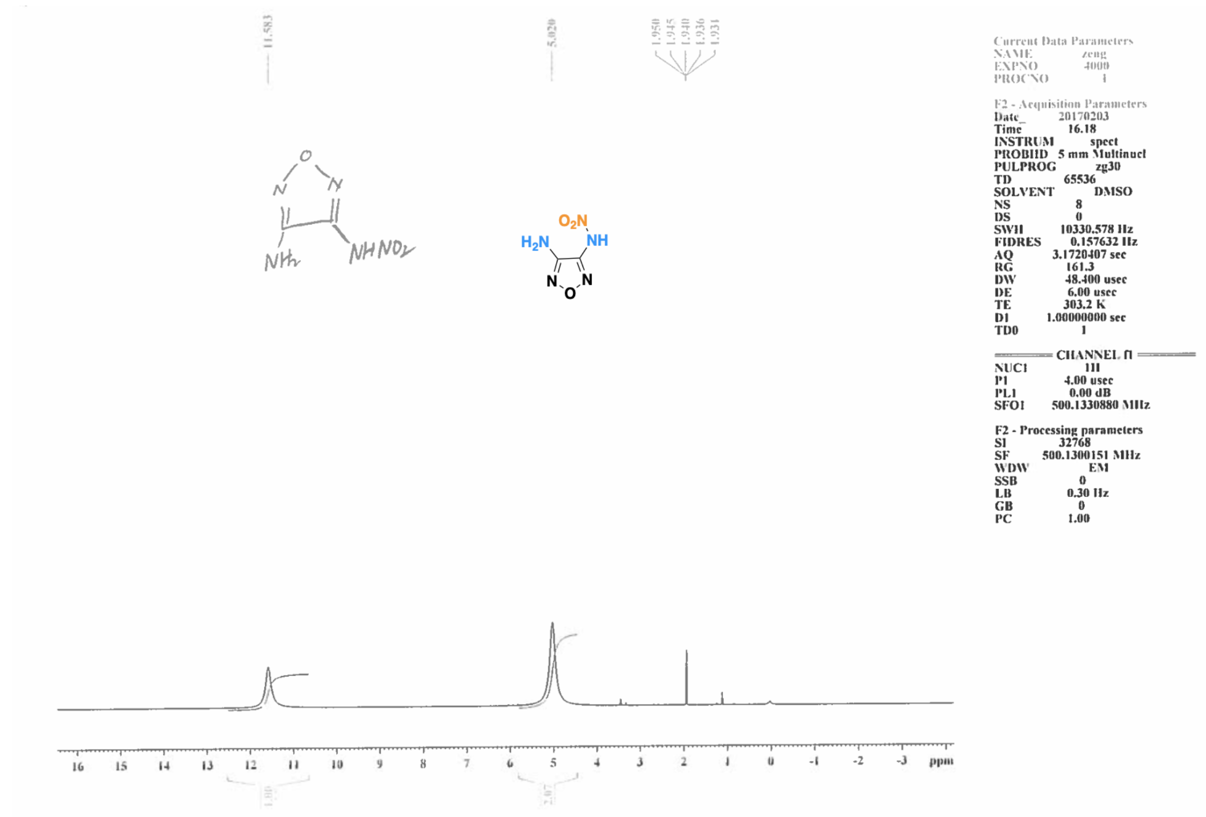

Supplementary Fig. 23. ^{13}C NMR of compound NF

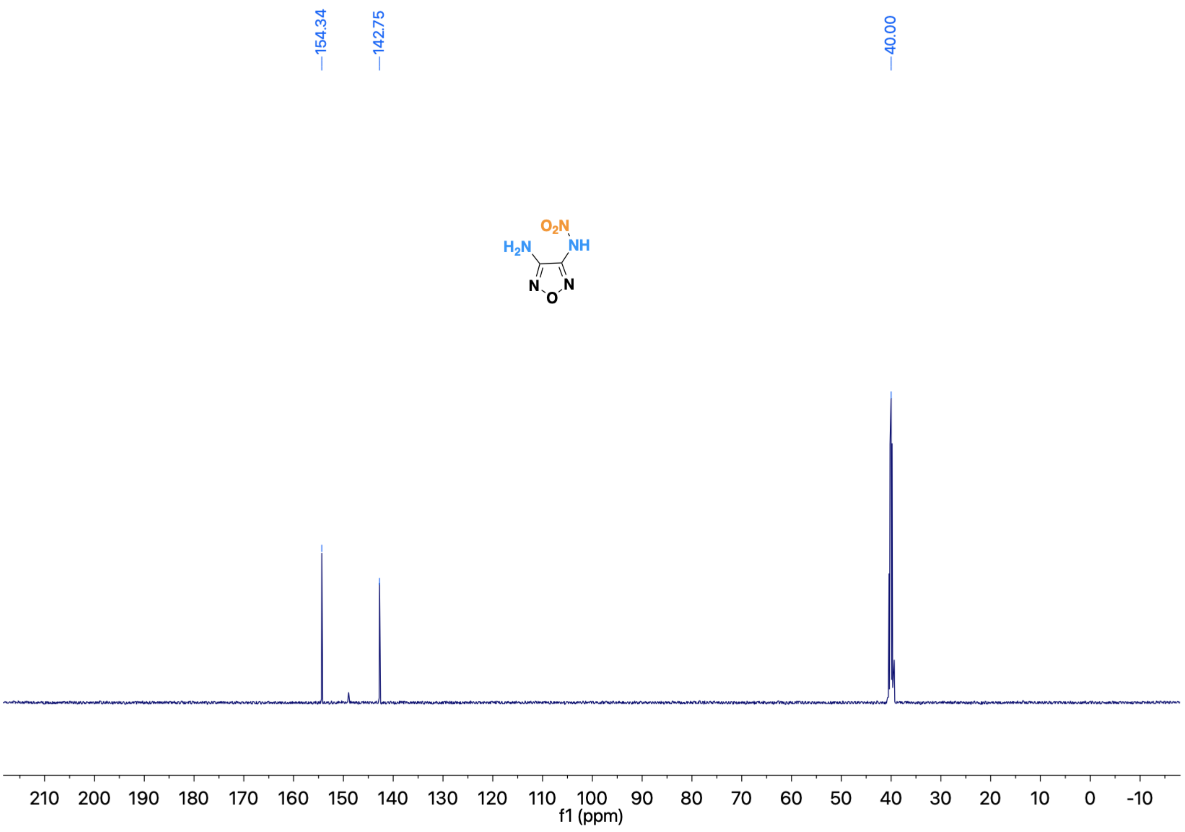

Supplementary Fig. 24. ^{1}H NMR of compound HHF-F

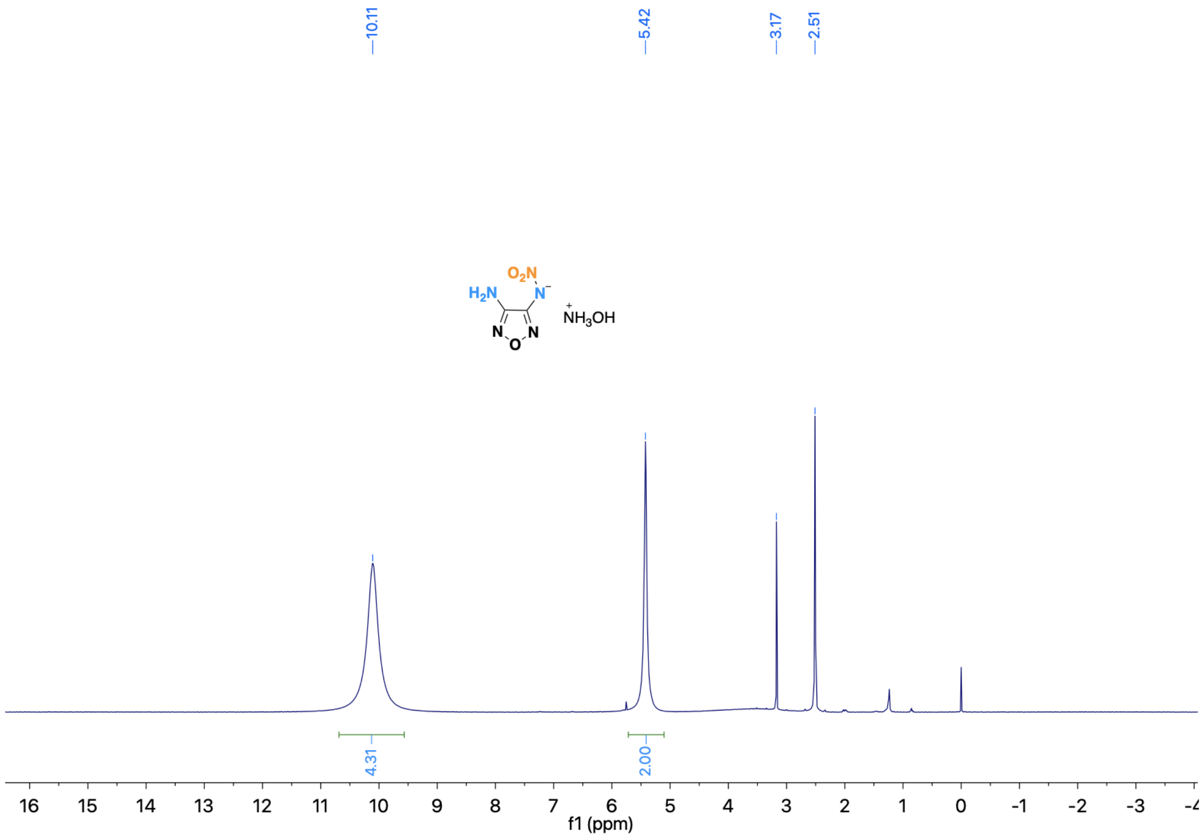

Supplementary Fig. 25. ^{13}C NMR of compound HHF-F

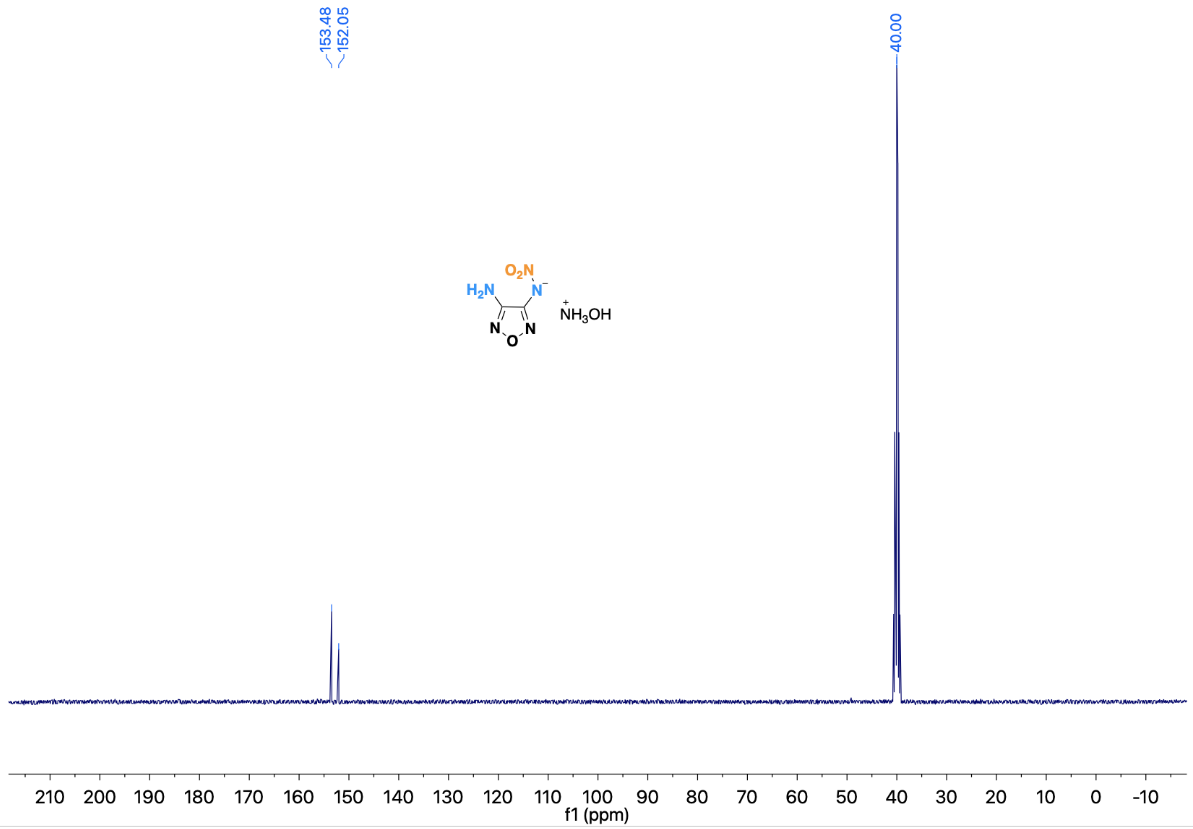

Supplementary Fig. 26. ^{1}H NMR of compound HHF-N4

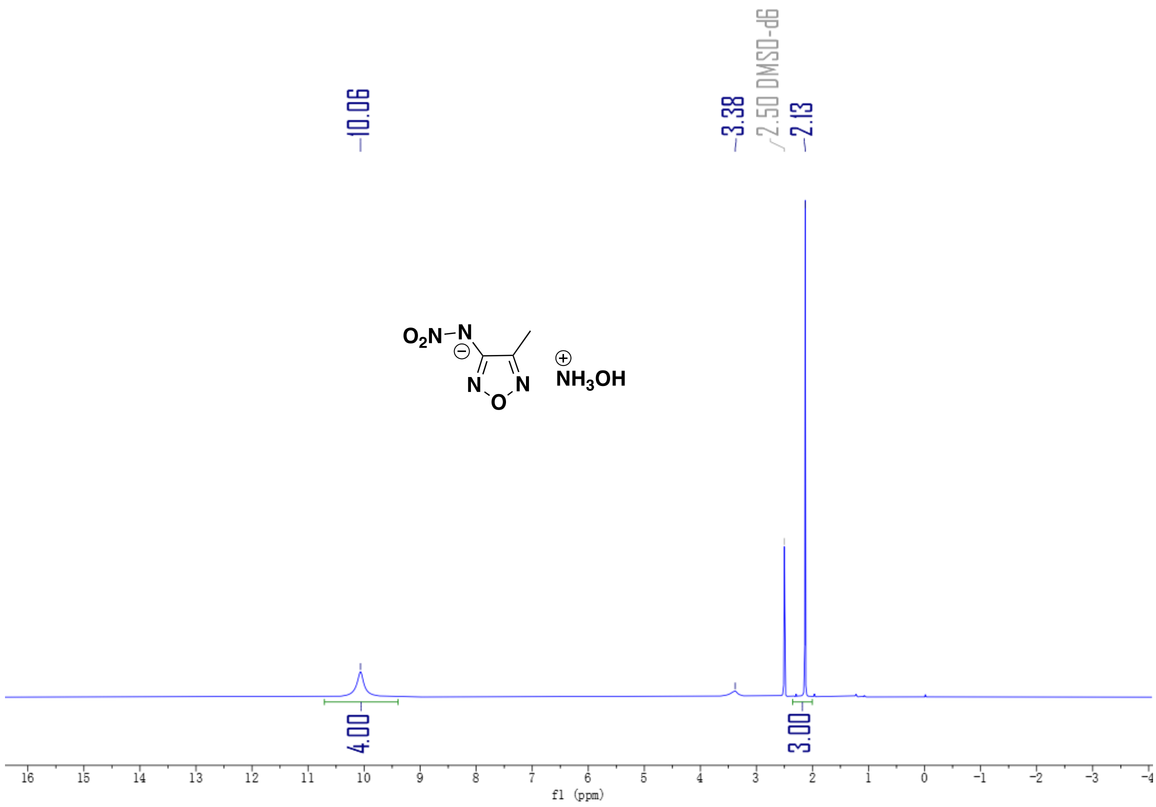

Supplementary Fig. 27. ^{13}C NMR of compound HHF-N4

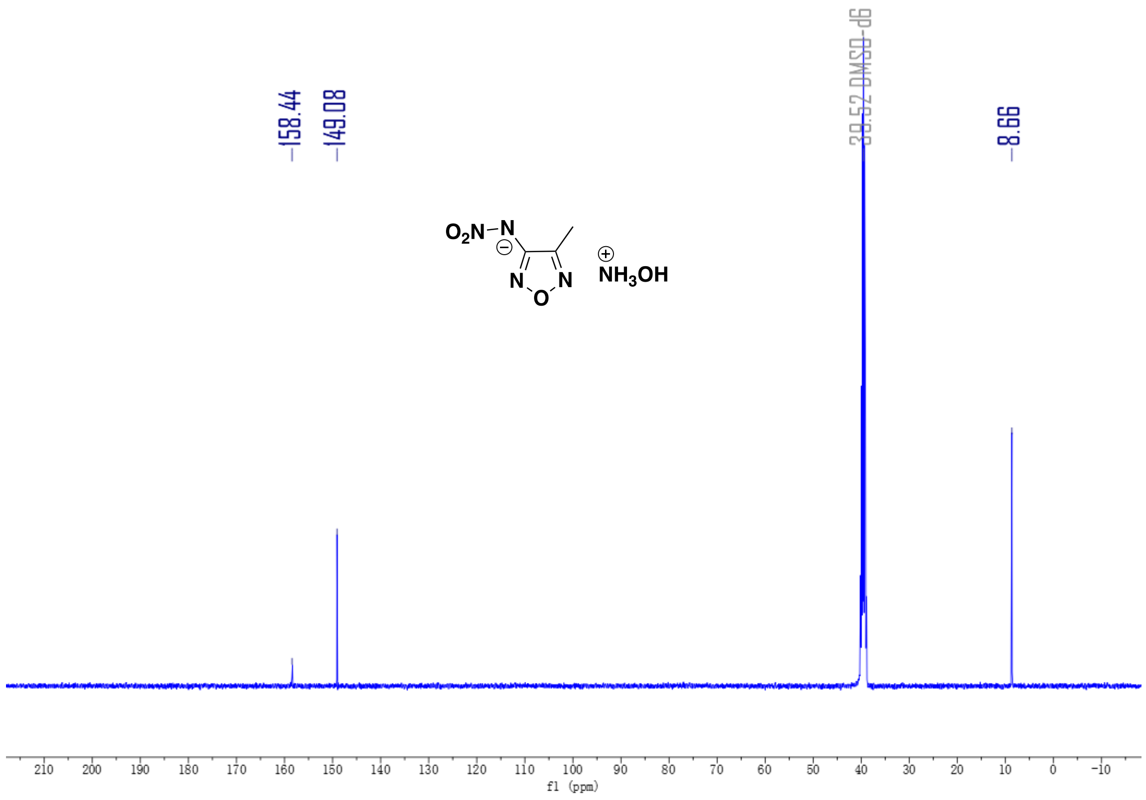

Supplementary Fig. 28. ^{1}H NMR of compound HHF-N14

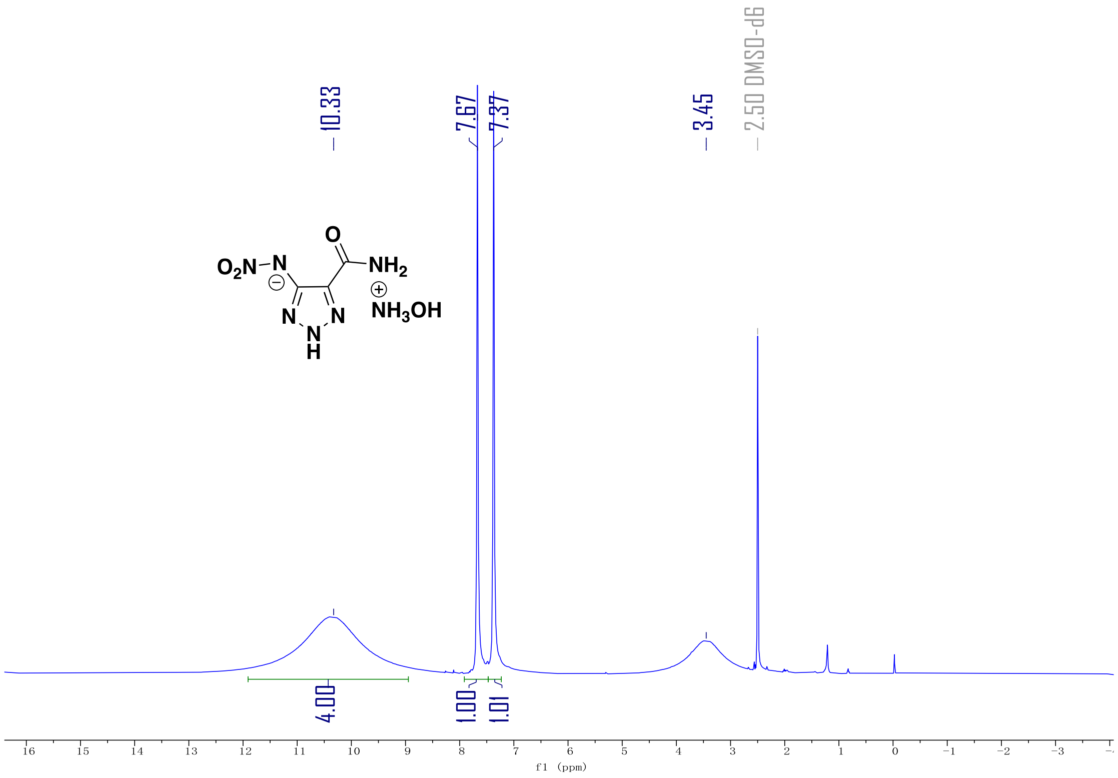

Supplementary Fig.29 ^{13}C NMR of compound HHF-N14

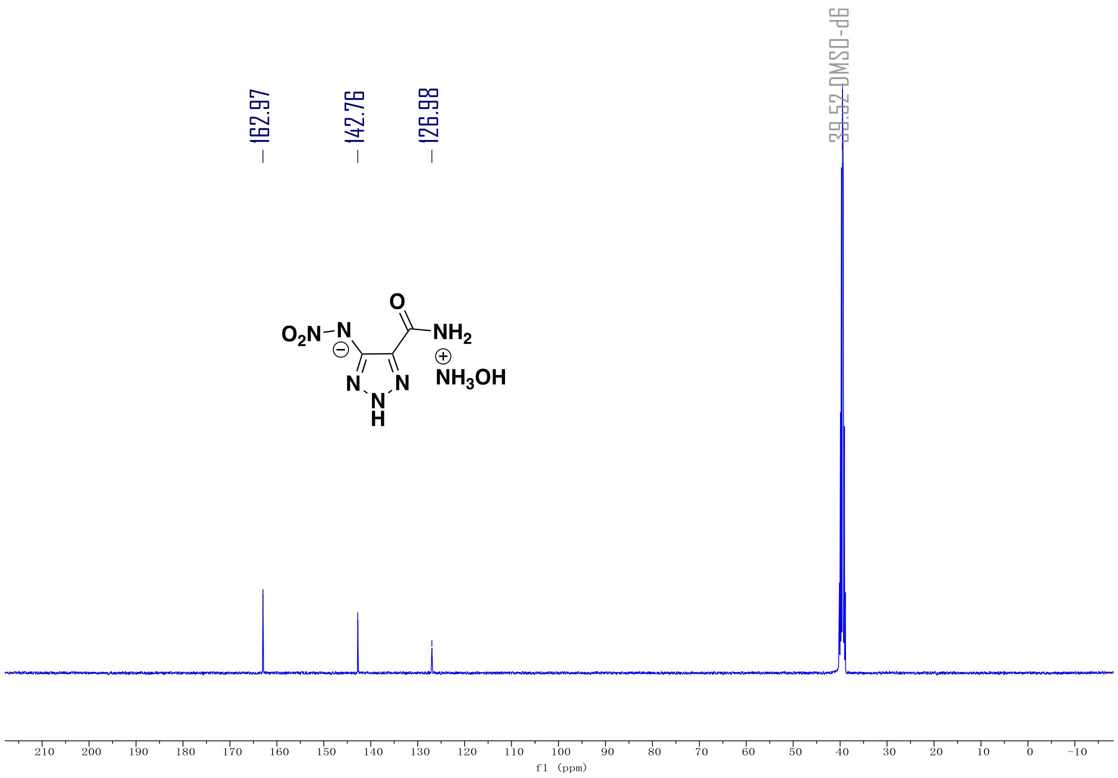

III. DSC Data

Supplementary Fig. 30. DSC of compound NT

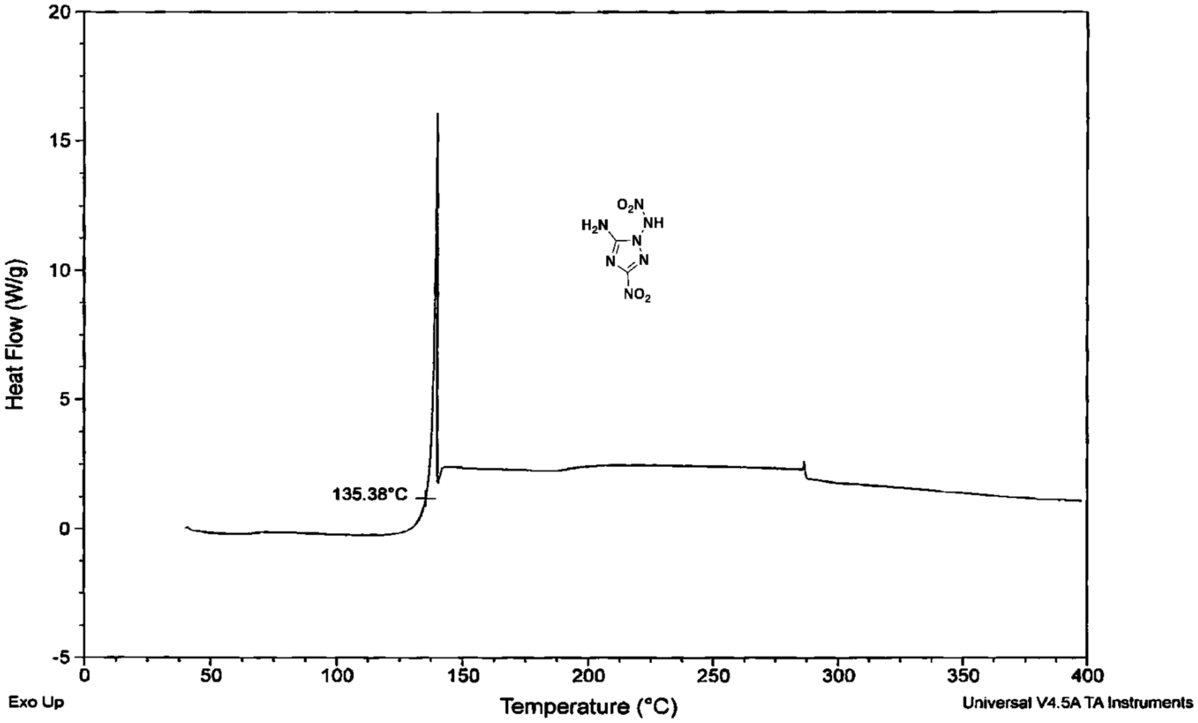

Supplementary Fig. 31. DSC of compound HHF-T

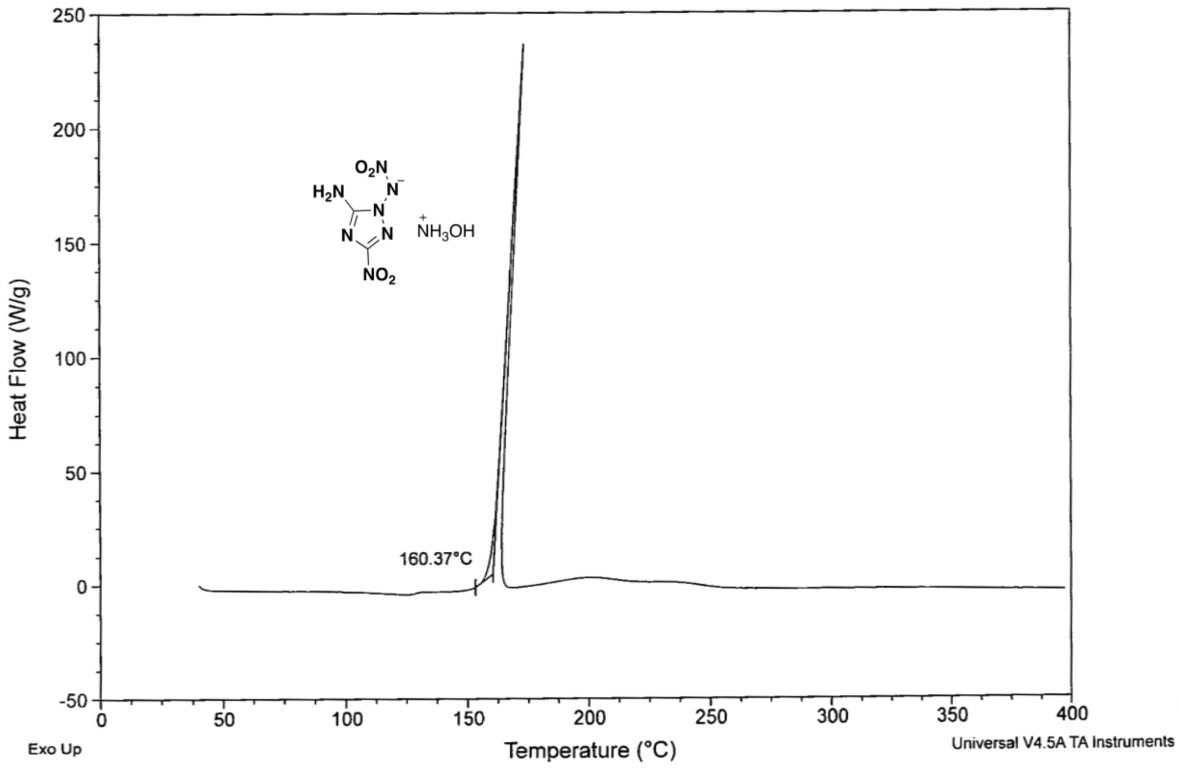

Supplementary Fig. 32. DSC of compound NF

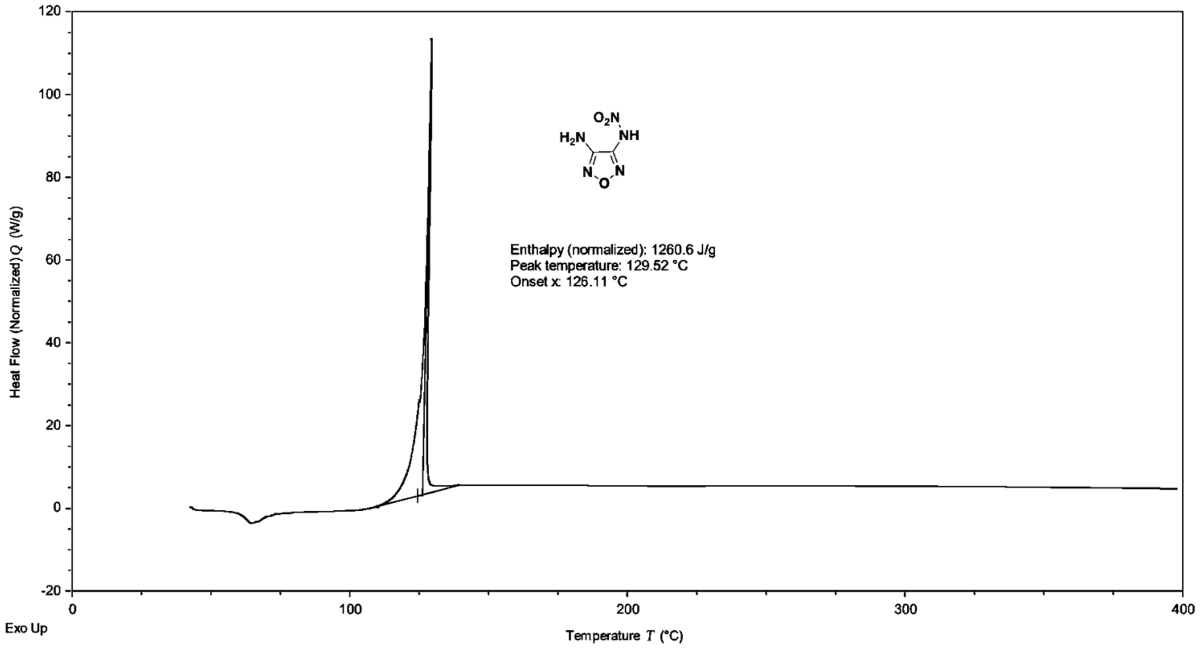

Supplementary Fig. 33. DSC of compound HHF-F

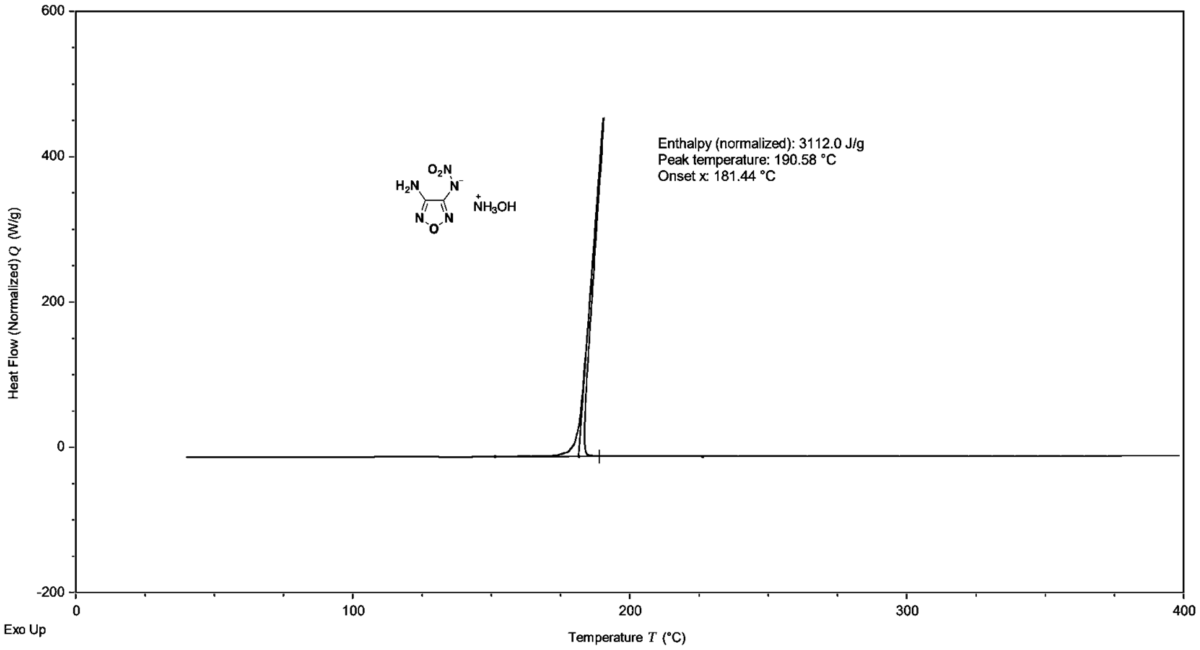

Supplementary Fig. 34. DSC of compound HHF-N4

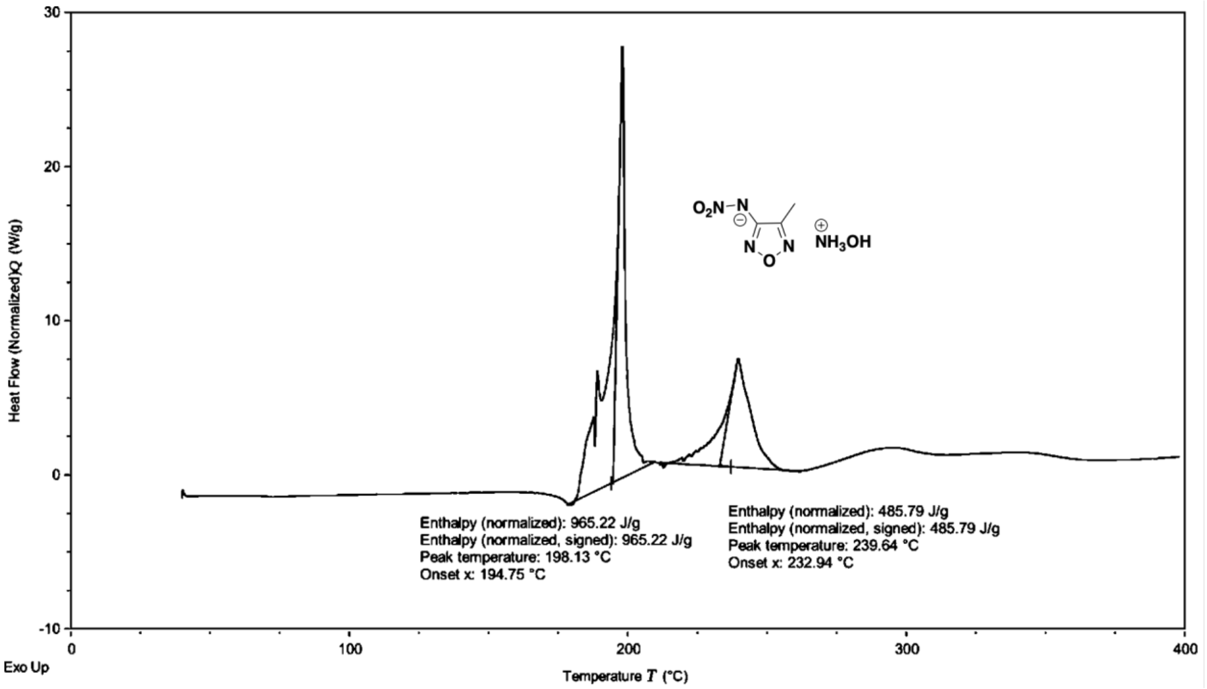

Supplementary Fig. 35. DSC of compound HHF-N14

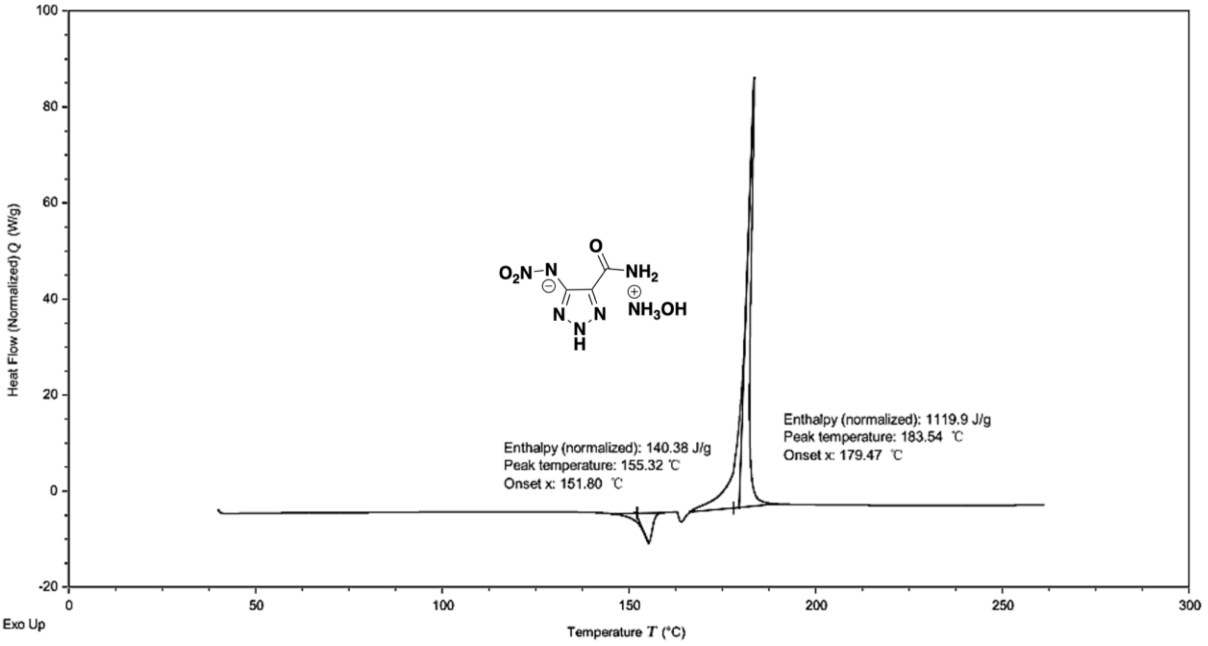

IV. Computation and properties

Packing Coefficient Calculations

Packing coefficients were calculated by using equation (1), where V_m and V_c are the molecular and crystal volumes, respectively. A volume enclosed through a surface with an assigned electronic density was considered as V_m. In this work, the electronic density was calculated at the theory level of M062x/6-311+G (d, p) and the density of 0.003 au was adopted for V_m calculations.

$$PC=\Sigma V_{mol} V_{cell}^{-1} \qquad (1)$$

2D fingerprint plot for intermolecular contact

Hirshfeld surfaces in a crystal are constructed in terms of the electron distribution, calculated as the sum of spherical atom electron densities.[1, 2] The normalized contact distance (d_{norm}) is determined by d_i and d_e, the distances from the surface to the nearest atom interior and exterior to the surface, respectively, and the van der Waals radii of the atoms. d_{norm} enables the identification of the regions of particular significance to intermolecular interactions. That is to say, a Hirshfeld surface is composed of lots of points, and each point parametrized as (d_i, d_e) can provide information about related contact distances from it. The smaller $d_i + d_e$ implies closer atom–atom contact. Both d_i and d_e were constrained in a range of 0-3.0 Å. Mapping these (d_i, d_e) points and considering their relative frequencies, we can get a two-dimensional (2D) fingerprint plot. For any symmetrically dependent molecule in any crystal, the fingerprint is unique. This is the basis for identifying a crystal environment of a given molecule. The color mapping distinguishes the intensity of points, and the red and the blue represent the high and low intensities, respectively. Therefore, through the locations of (d_i, d_e) points and their relative frequencies discernible on the surface and the 2D fingerprint plot, we can ascertain the distances and intensities of these contacts. All the fingerprint plots were created using CrystalExplorer 17.5[3] in this work, and the surfaces were mapped over a d_{norm} range of -0.4 to 1.4 Å.

Theoretical study of heat of formation

The geometric optimization and frequency analyses of the structures were calculated using M062X/6-31+G** level. [4,5] The heats of formation for N-(5-amino-3-nitro-1H-1,2,4-triazol-1-yl)nitramide, (5-amino-3-nitro-1H-1,2,4-triazol-1-yl)(nitro)amide ion, N-(4-amino-1,2,5-oxadiazol-3-yl)nitramide, (4-amino-1,2,5-oxadiazol-3-yl)(nitro)amide ion, hydroxylammonium ion were obtained by atomization approach using G2 composite method[6] (Supplementary Tab. 18). The gas-phase enthalpies of the molecules were obtained by using the atomization method with the G2 ab initio calculations.

Methods for estimating the diameter of guest molecules

A method[7] for estimating the molecular kinetic diameter based on the iso-electronic density surfaces. The Gaussian 09 program (Revision D.01) was used to calculate the geometric optimization and frequency analyses of the guest structure, and the PBE0/def2-TZVP level is used. subsequently, the coordinates of the iso-electronic density surfaces vertices are calculated

using Multiwfn, and finally the distance between the surface vertices is measured by VMD to derive the dimensions of the guest structure.

Supplementary Tab. 18. Enthalpies of the gas-phase species M (G2 method).

M	ΔH_f^o (kJ mol^{-1})
N-(5-amino-3-nitro-1H-1,2,4-triazol-1-yl)nitramide	320.697817
(5-amino-3-nitro-1H-1,2,4-triazol-1-yl)(nitro)amide	75.59139622
N-(4-amino-1,2,5-oxadiazol-3-yl)nitramide	232.5717802
(4-amino-1,2,5-oxadiazol-3-yl)(nitro)amide	51.92925468
hydroxylammonium	669.5240231

Based on the literature, the heat of sublimation is estimated with Trouton's rule.[8] The solid phase heat of formation of 1 was calculated with equation 1, in which T_d represents the decomposition temperature.[8]

$$\Delta H_f = \Delta H_f(g) - \Delta H_{sub} = \Delta H_f(g) - 188[\text{J mol}^{-1}\ \text{K}^{-1}] \times T_m \quad (1)$$

According to empirical formula (2)[9] the heat of sublimation can be computed from the temperature of melting (T represents either the melting point or the decomposition temperature when no melting occurs prior to decomposition).

$$\Delta H_{sub} = 188*(T+273.15)/1000 \qquad (2)$$

The solid phase heats of formation of **1** and **4** were calculated with eq 3.

$$\Delta H_{f,\ soliid} = \Delta H_{f,\ gas} - \Delta H_{sub} \qquad (3)$$

For energetic salts, the solid-phase heat of formation is calculated based on a Born-Haber energy cycle (**Supplementary Fig. 36.**).[9] The number is simplified by equation 4:

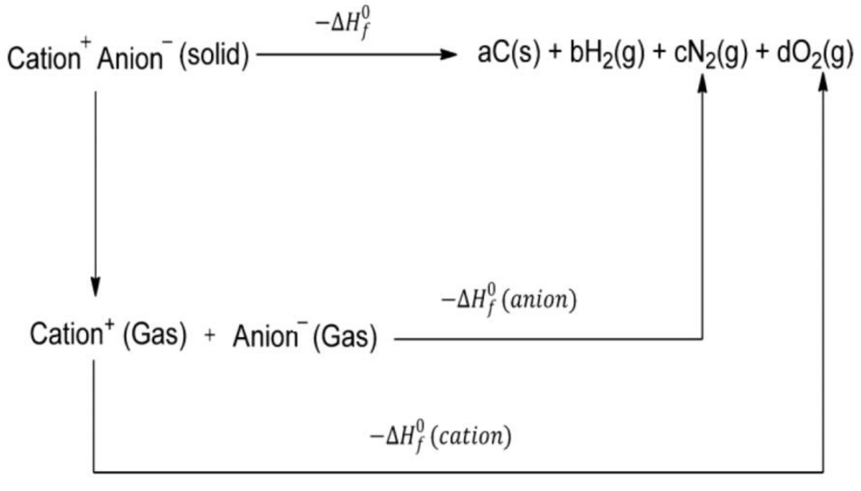

Supplementary Fig. 36. Born-Haber Cycle for the Formation of energetic salts.

ΔH_f^o (salt, 298 K) = ΔH_f^o (cation, 298K) + ΔH_f^o (anion, 298K) – ΔH_L (4)

in which ΔH_L can be predicted by using the formula suggested by Jenkins, et al.[9] (equation 5):

$$\Delta H_L = U_{pot} + [p(n_M/2 - 2) + q(n_X/2 - 2)]RT \qquad (5)$$

In this equation, n_M and n_X depend on the nature of the ions Mp^+ and Xq^-, respectively. The equation for lattice potential energy U_{pot} (equation 6) has the form:[9]

$$U_{POT}\ [\mathrm{kJ\ mol^{-1}}] = \gamma(\rho_m/M_m)^{1/3} + \delta \qquad (6)$$

where ρ_m [g cm^{-3}] is the density of the salt, M_m is the chemical formula mass of the ionic material, and values for g and the coefficients γ (kJ mol^{-1}cm) and δ (kJ mol^{-1}) are assigned literature values.[9] By using the measured room temperature densities, all the newly prepared compounds are listed in Supplementary Tab. 19.

Supplementary Tab. 19. The density, calculated lattice energy and calculated heat of formation.

compound	d[a] [g cm^3]	ΔH_L[b]	ΔH_f [c] [kJ mol^{-1}]
**NT**	1.791(173K)	-	243.97
**HHF-T**	1.812(173K)	500.2	239.95
**NF**	1.737	-	157.53
**HHF-F**	1.819	533.6	182.85

[a] Density was calculated based on a single crystal at room temperature. [b] Calculated lattice energy. [c] Calculated heat of formation.

Supplementary Tab. 20. Physical and detonation properties of energetic materials (**NT**, **HHF-T**, **NF, and HHF-F**).

Energetic compounds	Chemical Formula	T_d ^{a} (°C)	d ^{b} (g·cm^{-3})	ΔH_f ^{d} (kJ·mol^{-1})	D ^{e} (m·s^{-1})	P ^{f} (GPa)	OB(CO$_2$) ^{g} (%)	IS ^{h} (J)	FS ^{i} (N)
**NT**	$C_2H_3N_7O_4$	135	1.758^c	243.97	8769	32.7	-12.7	2	60
**HHF-T**	$C_2H_6N_8O_5$	160	1.779^c	239.95	9099	34.95	-14.4	10	160
**NF**	$C_2H_3N_5O_3$	126	1.737	157.53	8433	29.17	-27.57	10	160
**HHF-F**	$C_2H_6N_6O_4$	181	1.819	182.85	9286	35.85	-26.95	50	360

a Decomposition temperature (onset). b Density ((calculated based on a single crystal at room temperature). c Single crystal density calculated at 170 °C. d Heat of formation (calculated). e Detonation velocity (calculated). f Detonation pressure (calculated). g Oxygen balance (based on CO_2) for CaHbOcNd, 1600 (c–2a–0.5b)/MW, MW = molecular weight. h Impact sensitivity. i Friction sensitivity.

The enthalpy of formation ($\Delta_f H°$) is a significant parameter to evaluate the energy performance of EMs. The constant-volume combustion heat ($\Delta_c U°$) measurement of the HHF-F was carried out by oxygen bomb calorimetry (Parr 6200 Oxygen bomb calorimeter (NLFRM-45)), and the test value of $\Delta_c U°$ is 9.23 MJ kg^{-1}. The standard molar enthalpy of combustion ($\Delta_c H°$) can be obtained according to the formula (Eq. 7).[10] The ideal combustion reaction equation of HHF-F is given in Eq. 8. The standard

enthalpy of formation ($\Delta_f H°$) of HHF-F was calculated from $\Delta_c H°$(Eq. 9, Hess's Law) to be -4.59 kJ mol^{-1}[$\Delta_f H°(CO_2, g) = –393.51$ kJ mol^{-1}; $\Delta_f H°(H_2O, l) = –285.83$ kJ mol^{-1}].

$$\Delta_c H° = \Delta_c U° + \Delta nRT \qquad (7)$$

$\Delta n = n_g(\text{products}) – n_g(\text{reactants})$ (n_g is the total molar amount of gases in the products or reactants, R = 8.314 J $mol^{-1}\cdot K^{-1}$, T = 298.15 K.

$$C_2H_6O_4N_6 + 7/2O_2 \rightarrow 2CO_2 + 3H_2O + 3N_2 \qquad (8)$$

$$\Delta_f H_m(C_2H_6N_4O_6, s) = [2\Delta_f H°(CO_2, g) + 3\ \Delta_f H°\ (H_2O, l)] – \Delta_c H°(\ C_2H_6N_4O_6, s) \qquad (9)$$

Computational analysis of internal stress

Any external mechanical force acting on an energetic material can lead to a certain shape change to produce strain and store mechanical energy.[11] When this mechanical energy exceeds the limit of the energetic material, decomposition will be activated and trigger a series of explosions.[12] In order to explore the different performance of HHF-F and NF on safety issues, a force field was established for both unit cells and their abilities to handle external stimuli were probed. To simplify the simulation, only vertical compression and horizontal sliding of the crystal unit cell were investigated since all direction stimuli can be split into these two types.[13,14] Therefore, the deformation potential (P) can be expressed by the energy difference before and after deformation for energetic materials. For convenient comparison, the value is converted from mol units into volume units by dividing by the unit cell volume (eq 10).

$$P = (E_{after\ def} - E_{before\ def})/V_{unit\ cell} \quad (10)$$

In this part of the calculations, single point energies were obtained from optimized structures using BLYP-D3 def2-SVP method[14] using ORCA 4.0.1[15]

$$V_{unit\ cell} = 0.001 \times M \times Z/d$$

M: molar mass; Z: Number of molecules in a cell; d: calculated density of single crystal.

Supplementary Tab. 21. Absolute energy and internal stress (P) of HHF-F.

Distance (Å)	SPE1[a] (hartrees)	P_{ob} $kJmol^{-1}/MJm^{-3}$	SPE2[b] (hartrees)	P_{oc} $kJmol^{-1}/MJm^{-3}$
0	-2832.1999	0/0	-2832.1999	0/0
0.1	-2832.1991	2.100/5.364	-2832.1994	1.313/3.353
0.2	-2832.1980	4.988/12.740	-2832.1989	2.626/6.707
0.3	-2832.1961	9.977/25.482	-2832.1977	5.776/14.753
0.4	-2832.1938	16.016/40.907	-2832.1962	9.714/24.810
0.5	-2832.1913	22.579/57.670	-2832.1946	13.915/35.54
0.6	-2832.1886	29.668/75.776	-2832.1927	18.903/48.281
0.7	-2832.1857	37.282/95.224	-2832.1905	24.680/63.036
0.8	-2832.1837	42.533/108.636	-2832.1890	28.617/73.092

[a] Single point energy of HHF-F after sliding along ob axix. [b] Internal stress for HHF-F after sliding along oc axis.

Supplementary Tab. 22. Absolute energy and internal stress (P) of NF.

Distance (Å)	SPE1[a] (hartrees)	P_{ob} $kJmol^{-1}/MJm^{-3}$	SPE2[b] (hartrees)	P_{oc} $kJmol^{-1}/MJm^{-3}$
0	-2306.3457	0/0	-2306.3457	0/0
0.1	-2306.3447	2.626/7.859	-2306.3455	0.526/1.574

0.2	-2306.3435	5.776/17.289	-2306.3452	1.312/3.929
0.3	-2306.3416	10.76/32.220	-2306.3447	2.625/7.859
0.4	-2306.3390	17.590/52.653	-2306.3440	4.463/13.360
0.5	-2306.3354	27.042/80.944	-2306.3432	6.564/19.647
0.6	-2306.3303	40.433/121.02	-2306.3421	9.452/28.291
0.7	-2306.3234	58.549/175.248	-2306.3407	13.128/39.293
0.8	-2306.3143	82.440/246.762	-2306.3390	24.607/73.653

[a] Single point energy of NF after sliding along ob axix. [b] Internal stress for NF after sliding along oc axis.

Supplementary Statistic works of Monocyclic N-rich azole hydroxylammonium salts

Based on the CCDC database, almost all monocyclic N-rich azole hydroxylammonium salts (a-w) were found in the literature.[16-32]

They are listed in **Supplementary Fig. 37.**, and their decomposition temperatures are listed in Supplementary Table 23.

From Supplementary Table 23, it is obvious that except for compounds d, h, k, l, and q, the decomposition temperatures of all monocyclic N-rich azole hydroxylammonium salts are lower than 170 °C.

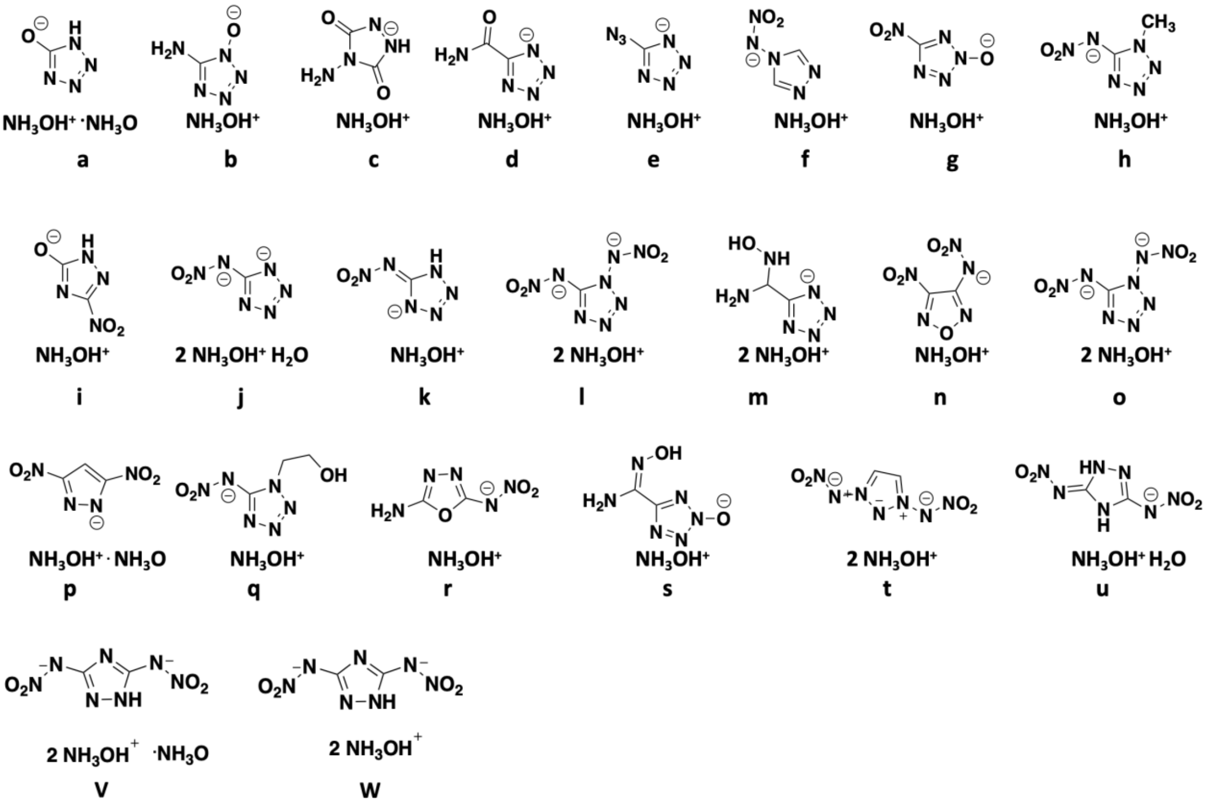

Supplementary Fig. 37. Structural formulas of reported monocyclic N-rich azole hydroxylammonium salts. According to the types of azole rings，the above structures can be

divided into substituted triazole (c, f, i, t, u-w); oxadiazole (n, r); tetrazole (a, b, d, e, g, h, j, k, l, m, o, q, s) and pyrazole (p).

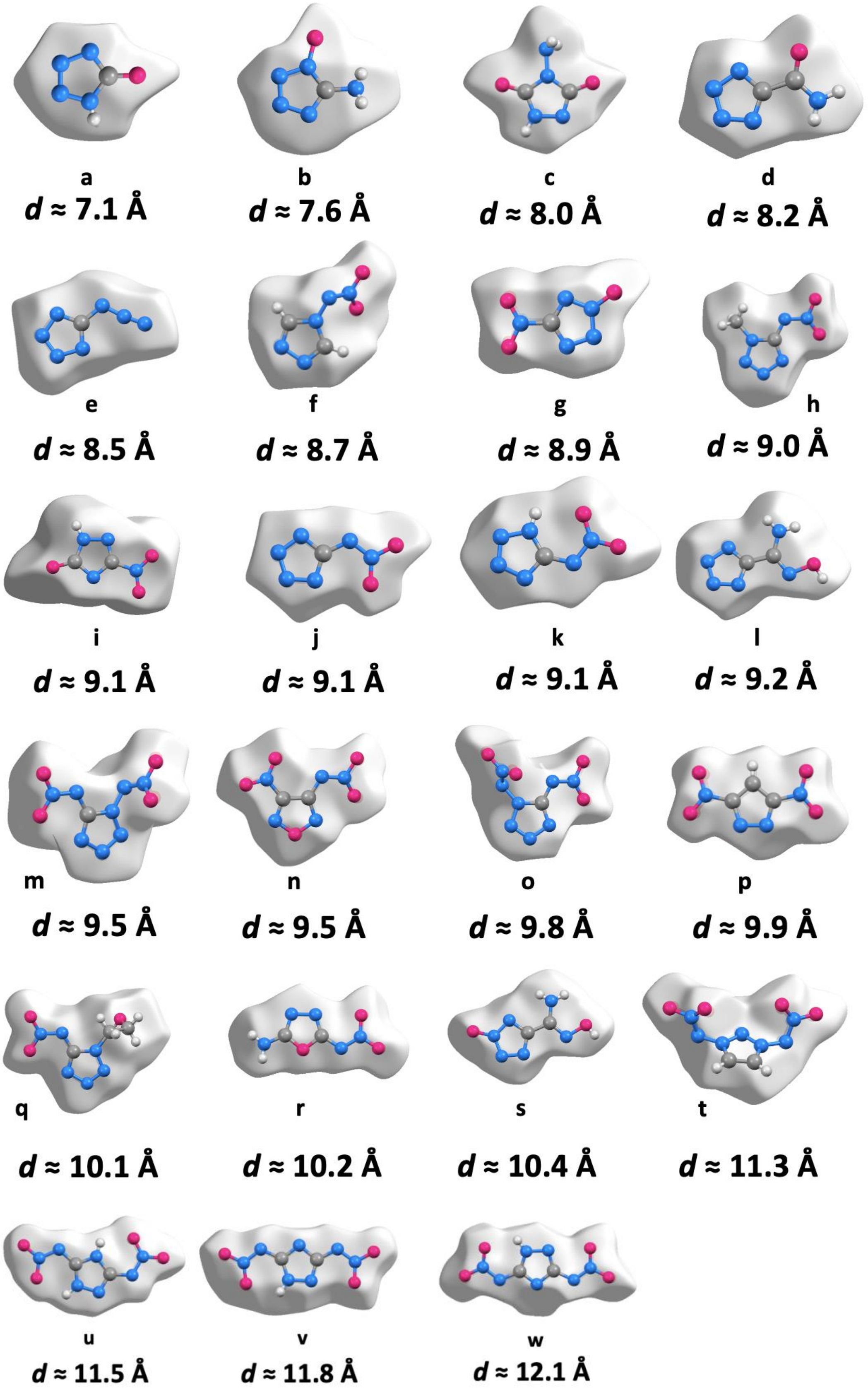

Supplementary Fig. 38. The diameter(d) of azole anions based on Hirshfeld surfaces of reported monocyclic N-rich azole hydroxylammonium salts. According to the size of the anion diameter, they can be summarized as follows: 7 <d ≤ 8 Å (a-c); 8 < d ≤9 Å (d-h); 9 <d ≤ 10 Å (i-p); 10 <d ≤ 11 Å (q-s); 11 <d ≤ 12 Å (t-v); 12 <d ≤ 13 Å (w).

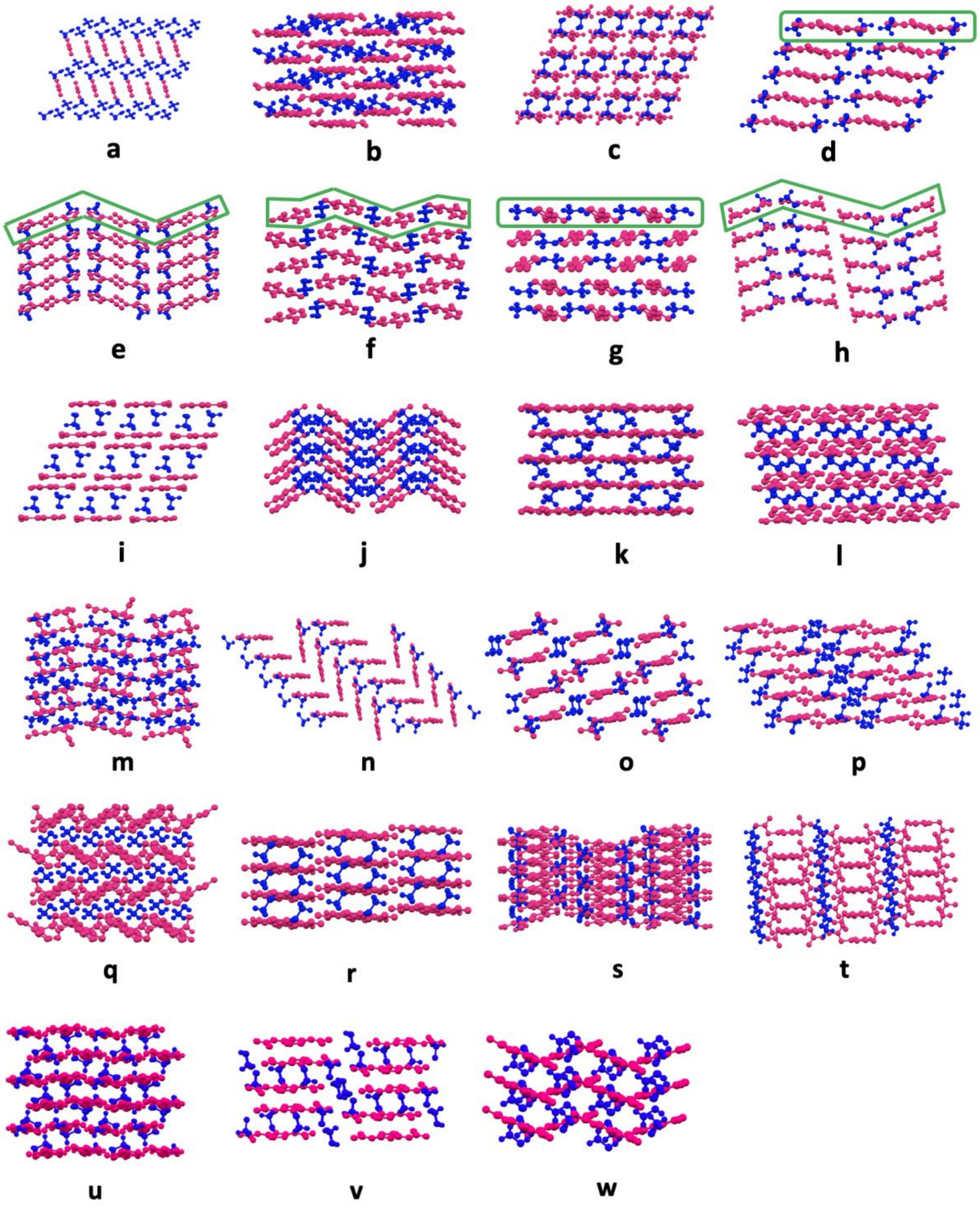

Supplementary Fig. 39. The crystal structure of reported monocyclic N-rich azole hydroxylammonium salts. To facilitate the observation of crystal packing, hydroxylammonium cations are blue, and azole anions are red. Examples of hydroxylammonium cations in layers

are marked with green boxes. The comparative analysis about crystal packing mode shows that most of them are sandwich-like (b, c, i, k-m, q-r, u, v), and the crystal structures of a, j, n, o, p, s, t, w show mixed packing mode, only five cases are layer-by-layer packing of the hydroxylammonium cation within the layer (d-h).

Supplementary Tab. 23. Decomposition temperatures and density of reported monocyclic N-rich azole hydroxylammonium salts

Compound	Decomposition (°C)	Density (g cm^{-3}) [a]	IS(J)	D (m s^{-1})	Note
**a**	138	1.634	>40	9034	Ref 16
**b**	155	1.664	10	9056	Ref 17
**c**	138	1.75(127k)	>40	8779	Ref 18
**d**	173	1.660	40	8180	Ref 19
**e**	96	1.649	<1	8959	Ref 20
**f**	146(Melting point)	1.74(103k)	17	8876	Ref 21
**g**	157	1.85	4	9499	Ref 22
**h**	185	1.627	8	8651	Ref 23
**i**	-	1.891(293k)	-	-	Ref 24
**j**	166	1.771	10	9214	Ref 23
**k**	172	1.785	2	9236	Ref 23
**l**	171	1.639(100k)	10	8614	Ref 25
**m**	-	1.848	-	-	Ref 26
**n**	140	1.875	10	9589	Ref 27
**o**	130	1.813(298k)	1.5	9807	Ref 26
**p**	141	1.772	-	-	Ref 28
**q**	178	1.614	15	8298	Ref 29
**r**	138	1.82(296k)	8	9087	Ref 30
**s**	164	1.704(100k)	>40	8934	Ref 25
**t**	149	1.804	1	9426	Ref 31
**u**	145	1.881[b]	3	9313	Ref 32
**v**	165	1.848[b]	10	9690	Ref 32
**w**	147	1.836[b]	4	9297	Ref 32

a. Density was calculated based on a single crystal at 173 K. b. Measured densities: gas pycnometer at room temperature.

The impact sensitivity of compounds a, c, d, and s in the above example is insensitive (is greater than or equal to 40 J), because none of these N-rich azole anions contain energetic substituents such as nitro or nitramine groups.

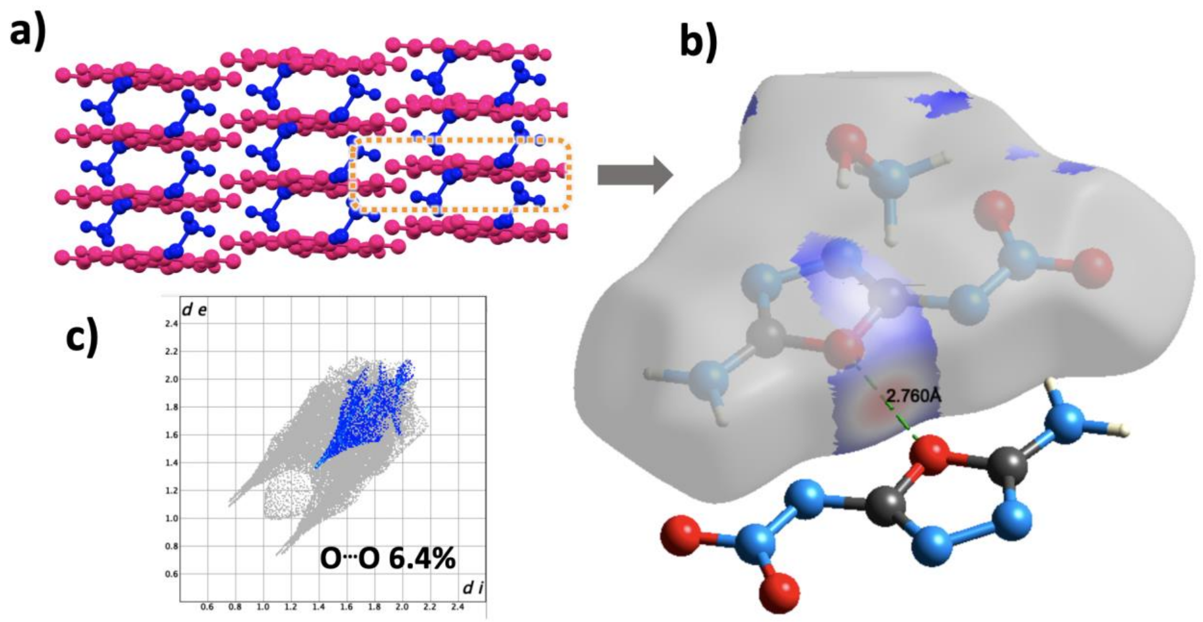

Supplementary Fig. 40. a) Crystal structure of hydroxylammonium 2-amino-5-nitramino-1,3,4-oxadiazolate (**r**); **b**) The part of the Hirshfeld surface attributed to the oxygen interaction; **c**) Highlight O···O contacts of 2D fingerprint plots in crystal stacking for hydroxylammonium 2-amino-5-nitramino-1,3,4-oxadiazolate (**r**).

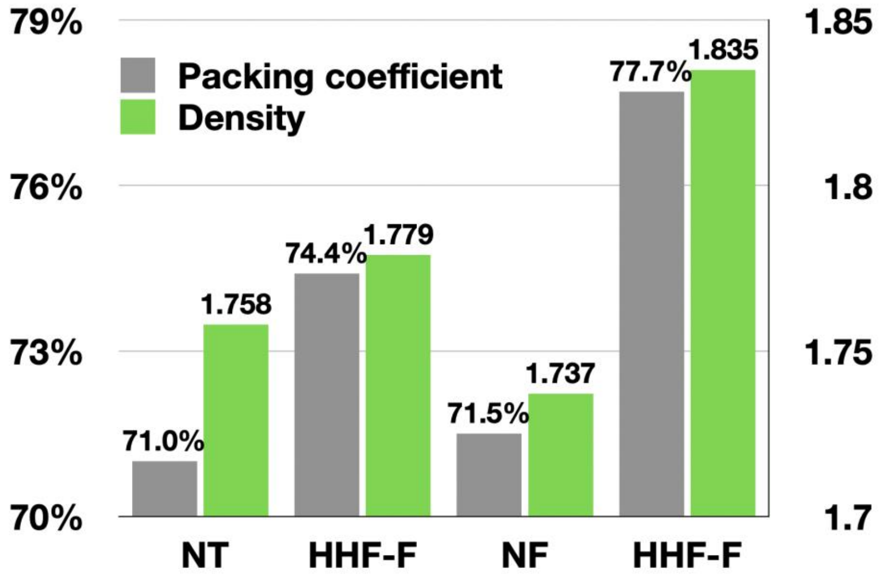

Supplementary Fig. 41. Crystal density (g cm^{-3}) and packing coefficient (NT, HHF-T, and HHF-F: 173 K; NF: 296 K).

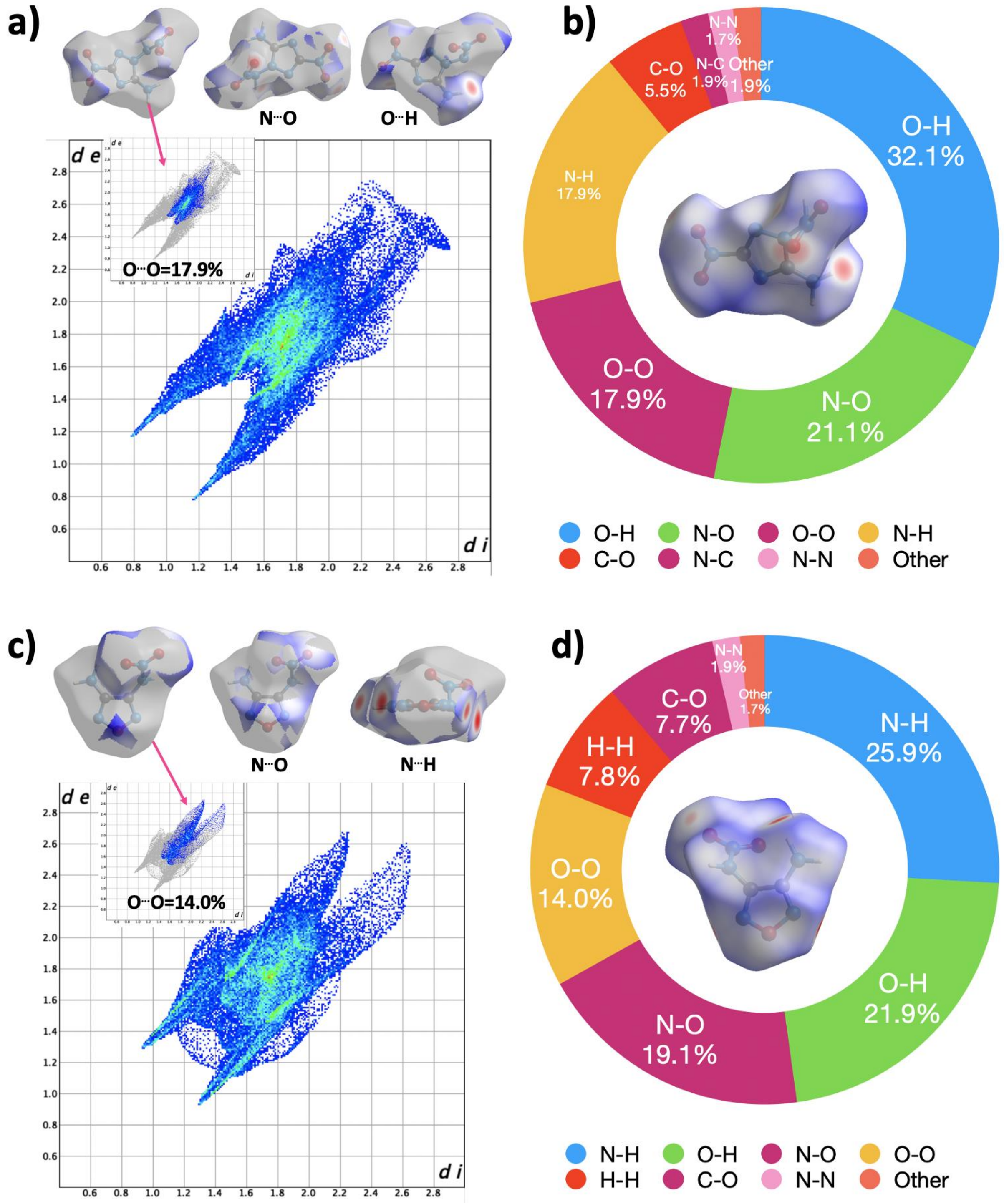

Supplementary Fig. 42. The 2D fingerprint plots in crystal stacking for NT(a) and NF(c); Pie graphs for NT (b) and NF (d) show the percentage contributions of the individual atomic contacts to the Hirshfeld surface, Hirshfeld surfaces (inside).

V Application expansion of size matching strategy

For N-rich azole anions, the combination of different skeletons and substituents can get various structures of different sizes. In general, for the monocyclic systems studied in this work, their dimensions are mostly in the range of 7-15 Å.

According to the source classification of acid hydrogen in N-rich azoles for energetic hydroxylammonium salts in the CSD database, the most abundant ones are nitroamino- and nitro-substituted heterocycles, respectively. The acidic hydrogen of HHF-F and HHF-T reported in this work is derived from nitroamine-based structures.

In order to expand the application of this strategy, we construct the nitroamine unit based on the monocyclic N-rich azoles as the skeleton and introduce the nitro group, amino group, alkyl group, amide group, and carboxylic acid in other positions to adjust the guest anion size. Thus, a candidate library of guest anions is established.

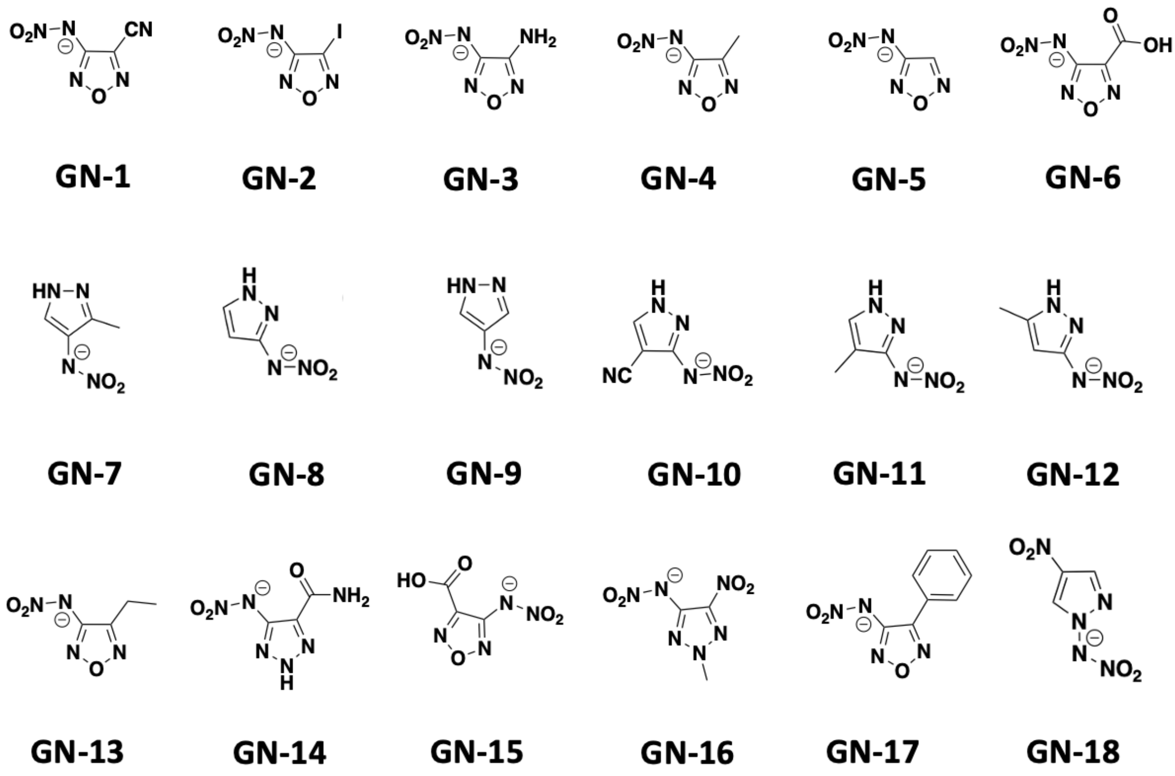

Supplementary Fig. 43. Guest-Nitramidate (GN)， according to the types of azole rings， the above structures can be divided into substituted triazole (GN-14, 16); oxadiazole (GN-1-6, 13, 15, 17) and pyrazole (GN-7-12, 18).

Then, theoretical calculation and prediction of the size of the above structure are carried out, and the predicted size is shown in the figure below:

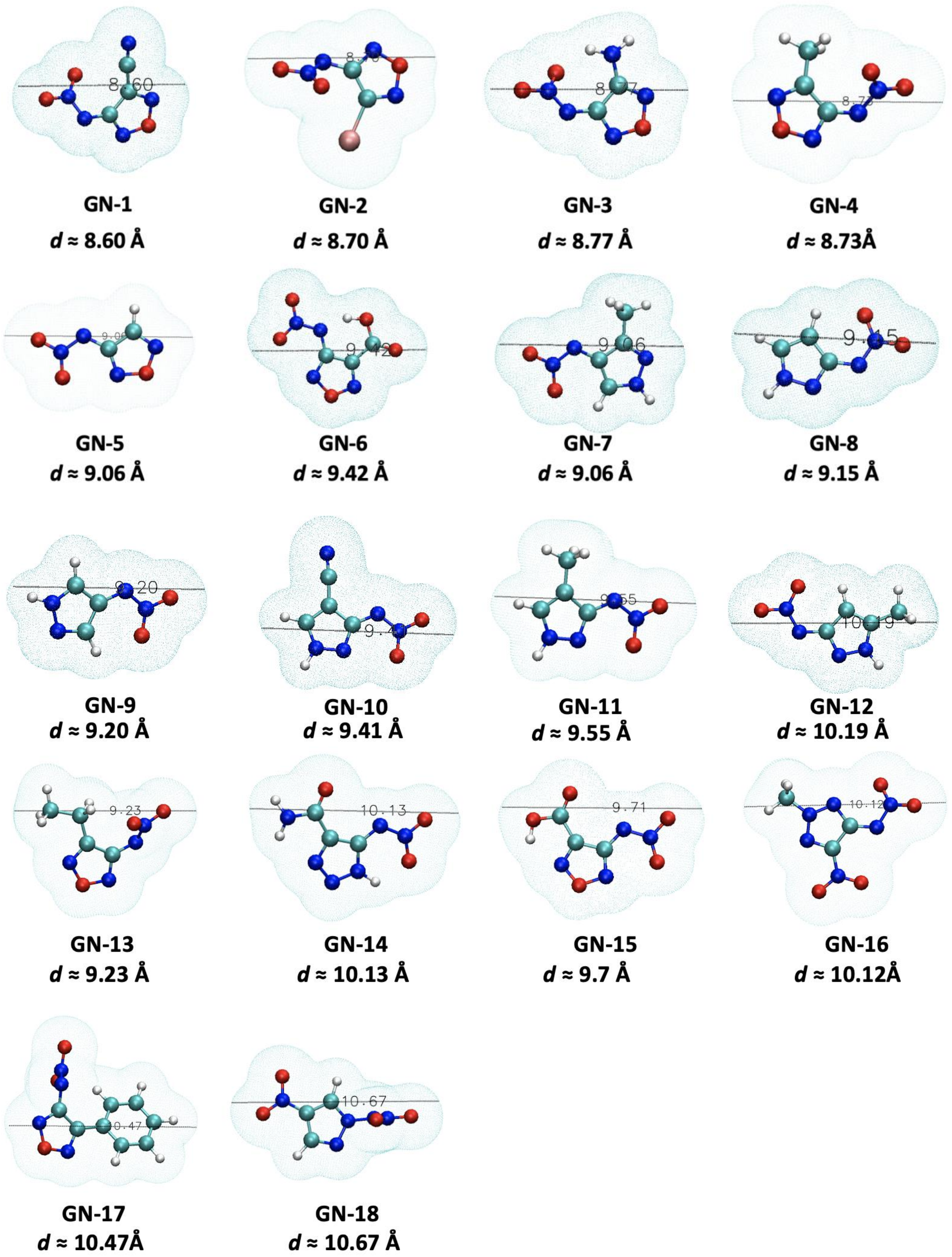

Supplementary Fig. 44. The predicted diameter of guest azole anions based on theoretical calculations. According to the size of the anion diameter, they can be summarized as follows: 8 < d ≤9 Å (GN-1-4); 9 <d ≤ 10 Å (GN-5-11, 13, 15); 10 <d ≤ 11 Å (GN-12, 14, 16-18).

According to the results of theoretical calculation, the diameter of the anionic guest GN1-4 is between 8-9 Å. Due to the large volume of the hydroxylamine structure itself, there is no

structure whose predicted diameter is less than 8 Å. The GN-3 (8.8 Å) is the guest structure of HHF-F which is mainly discussed in this paper, and its predicted diameter is very close to the actual Hershfield surface diameter (8.5 Å) measured by the crystal structure.

We attempted to synthesize the above structures to verify the correlation between the predicted size and the actual crystal packing. For the above synthesis of hydroxylammonium salts containing nitramine groups, we used the following general steps：

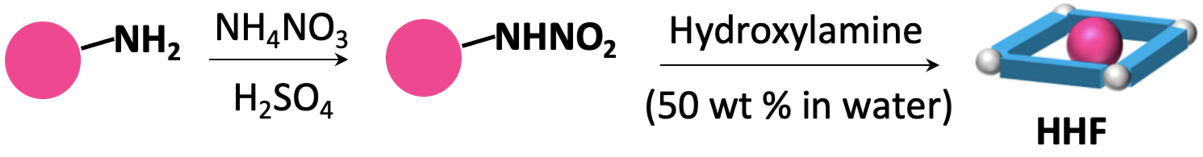

Supplementary Fig. 45. The general synthesis steps for HHF

With reference to the size prediction results, we prepared the crystals of hydroxylammonium salts corresponding to GN-1, GN-2, GN-3 and GN-4 with the guest anion size in the range of 8-9 Å. Also to verify the feasibility of the strategy, some hydroxylammonium salts with guest anion sizes larger than 10 Å were selected for synthesis. The crystal data of the following two compounds HHF-N4(hydroxylammonium (4-methyl-1,2,5-oxadiazol-3-yl)(nitro)amide) and HHF-N14 (hydroxylammonium 4-carbamoyl-5-nitramino-2H-1,2,3-triazole) were obtained. In addition, we obtained ammonium salt crystals of GN-16 (ammonium (5-carbamoyl-2-methyl-2H-1,2,3-triazol-4-yl)(nitro)amide, crystal-N-1) during the incubation of the hydroxymonium salt crystals of GN-16 (Supplementary Fig. S46).

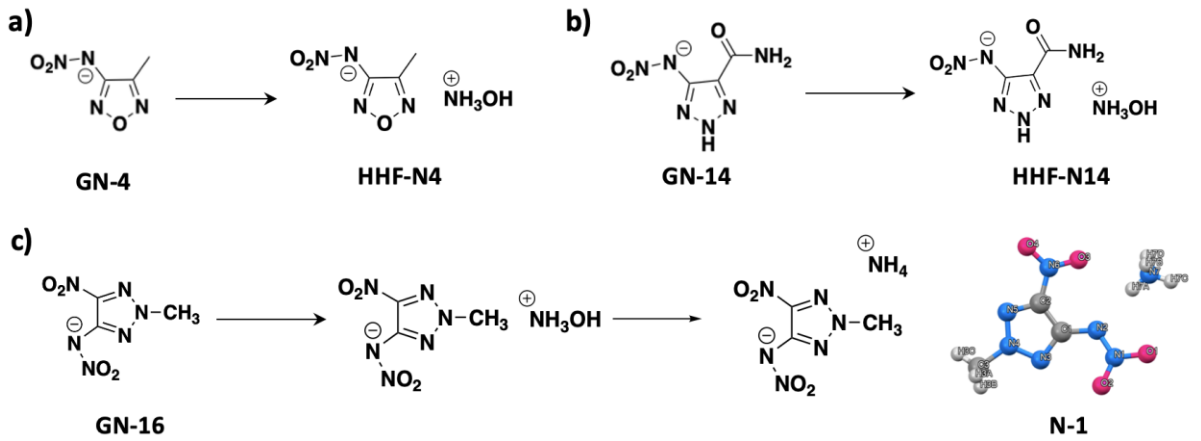

Supplementary Fig. 46. a) GN-4 as guest to form HHF-N4 (hydroxylammonium (4-methyl-1,2,5-oxadiazol-3-yl)(nitro)amide) **b)** GN-14 as guest to form HHF-N14 (hydroxylammonium 4-carbamoyl-5-nitramino-2H-1,2,3-triazole) **c)** GN-16 as guest to form ammonium (5-carbamoyl-2-methyl-2H-1,2,3-triazol-4-yl)(nitro)amide(N-1).

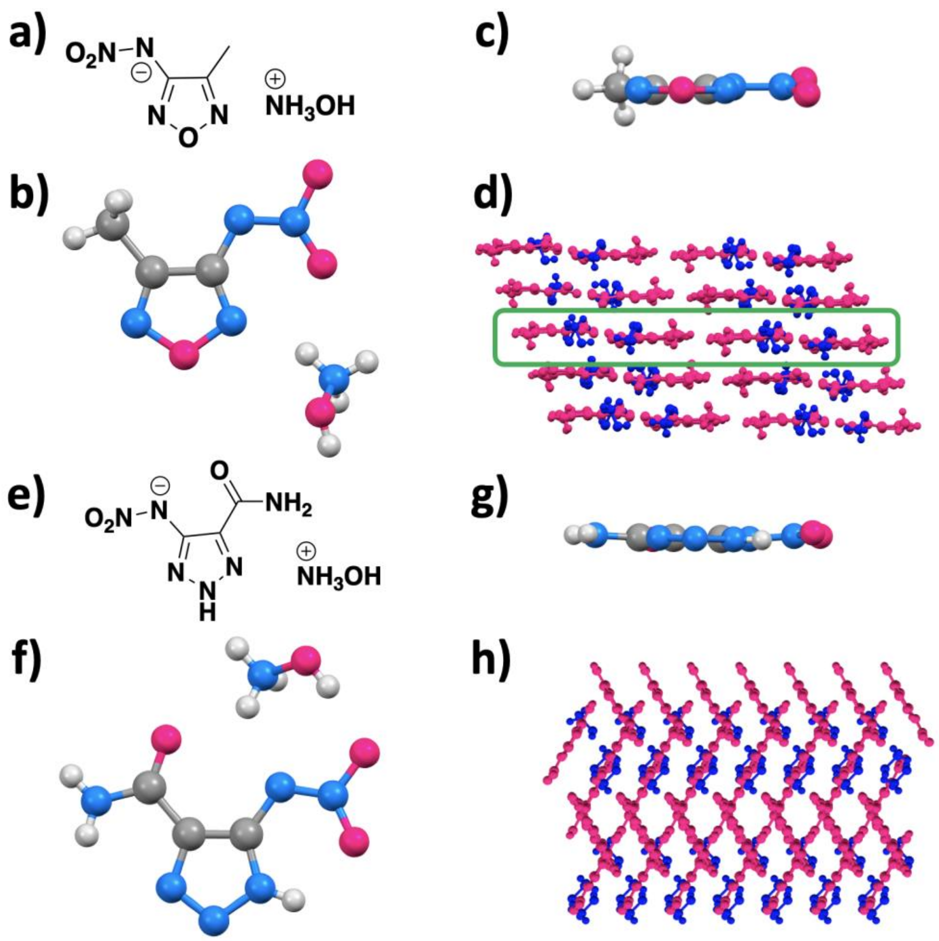

Supplementary Fig. 47. a) and **e)** Molecular structure and crystal packing of HHF-N4 and HHF-N14. **b)** and **f)** Molecular structure of HHF-N4 and HHF-N14. **c)** and **g)** Planar molecular geometry of HHF-N4 and HHF-N14. **d)** and **h)** Crystal stacking structures of HHF-N4 and HHF-N14.

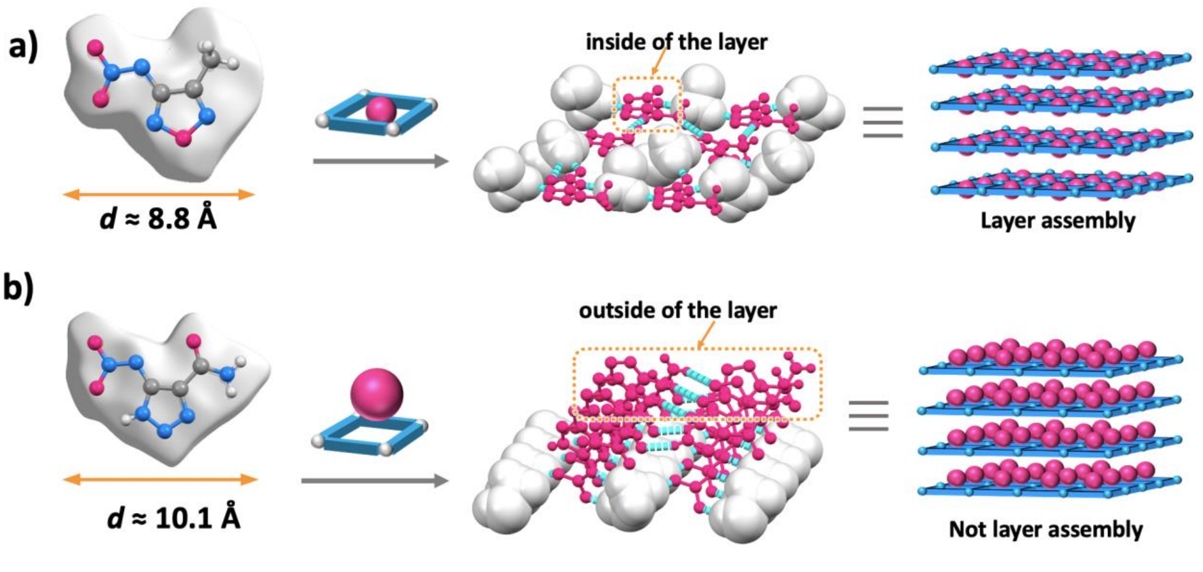

Supplementary Fig. 48. a) Layer assembly mode of HHF-N4 and its crystal packing diagram. **b**) Layer assembly mode of HHF-N14 and its crystal packing diagram.

From the above supplementary figure 47 and 48, the guest molecules in HHF-N4 are successfully embedded in the hydrogen bond framework composed of ammonium hydroxide, displaying face-to-face stacking. Analysis of the above two compounds, HHF-N4 and HHF-N14 by two-dimensional fingerprints and Hirshfeld surfaces revealed that HHF-N4 (8.8 Å), which has guest anion sizes of 8-9 Å, also has more O...H interactions in the crystal structure than HHF-N14 (10.1 Å). This means that for the nitro group, more hydrogen bonds can be made to stabilize it.

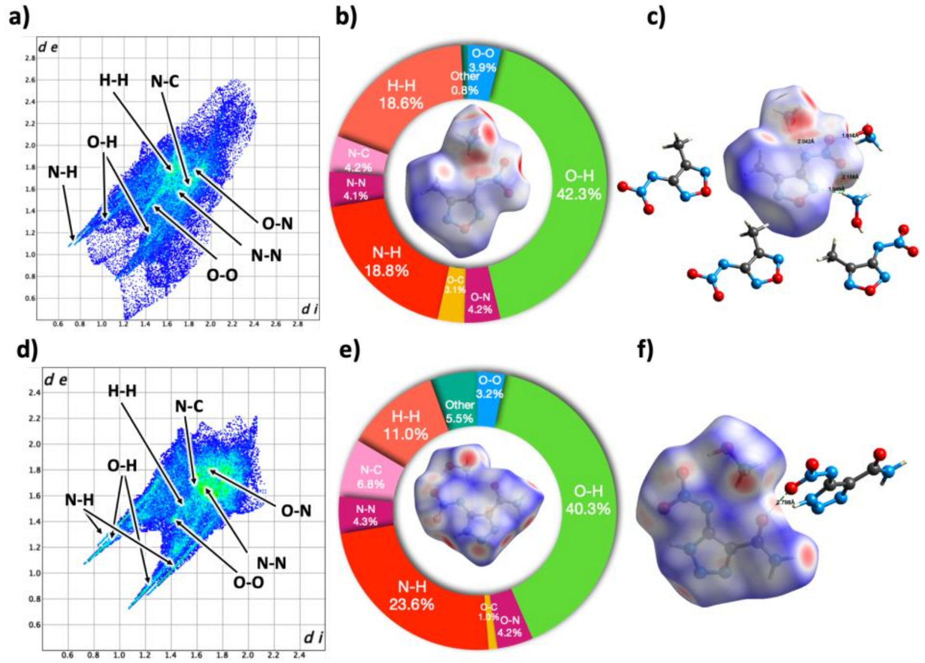

Supplementary Fig. 49. The 2D fingerprint plots in crystal stacking for HHF-N4 (**a**) and HHF-N14 (**d**); Pie graphs for HHF-N4 (**b**) and HHF-N14 (**e**) show the percentage contributions of the individual atomic contacts to the Hirshfeld surface, Hirshfeld surfaces (inside). Short contact for HHF-N4 (**c**) and HHF-N14 (**f**).

In this part, the Hirshfeld surface diameter of the guest anion is in good agreement with the predicted value, and their crystal packing mode is consistent with the conclusion that for a guest with size in the range of 8 to 9 Å, a more orderly crystal packing can be obtained. This indicates that the size-match strategy can be applied in this above series of N-rich azole anion guests. In addition, we extended our size-matching strategy to structure prediction of hydroxylamine hydrogen bond framework using a series of anions guest formed by N-rich heterocyclic azoles with acid hydrogen.

In addition, we extended our size-matching strategy to structure prediction of hydroxylamine hydrogen bond framework using a series of anions guest formed by N-rich heterocyclic azoles with acid hydrogen.

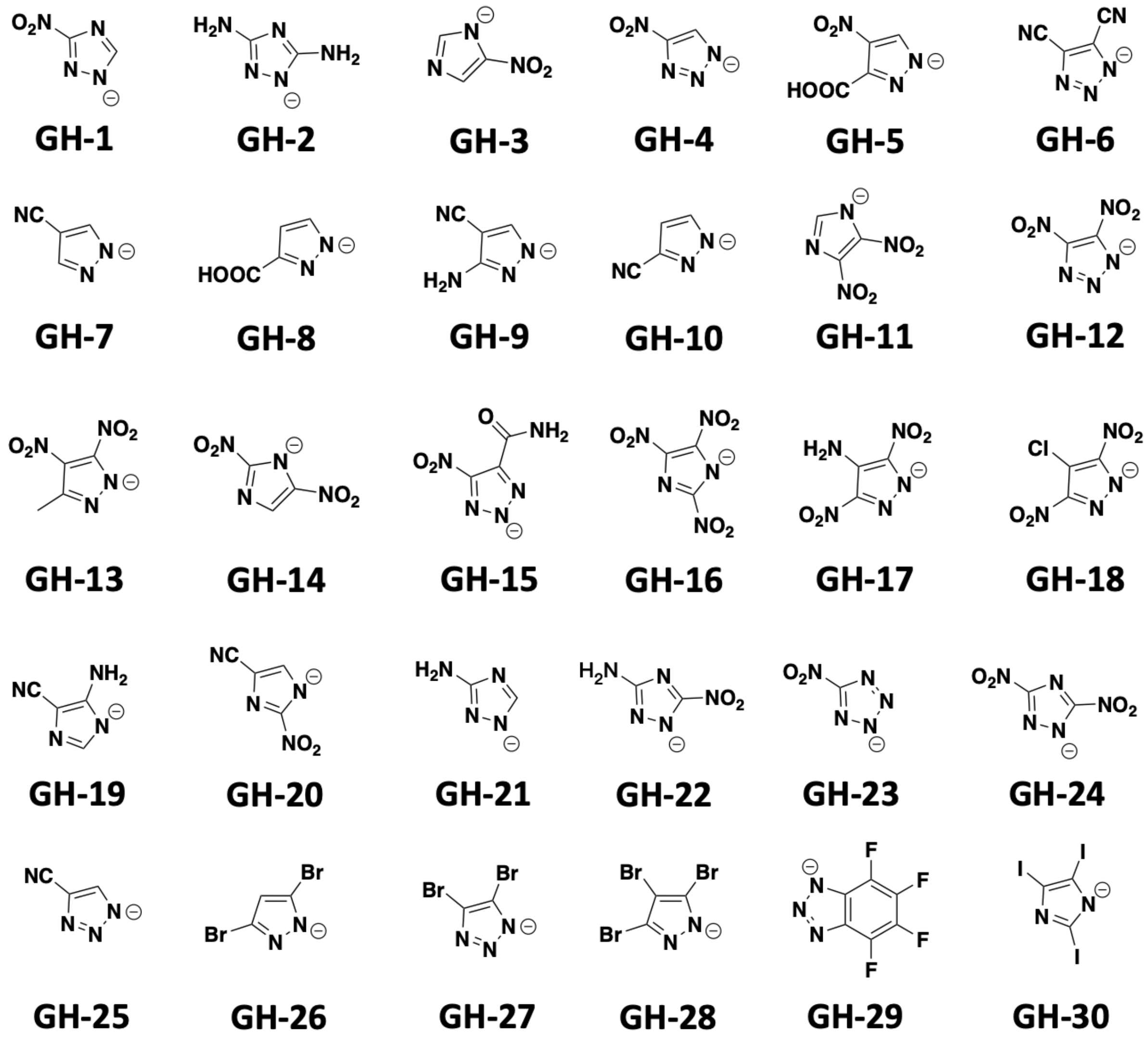

Supplementary Fig. 50. Guest-Heterocycle (GH), according to the types of azole rings，the above structures can be divided into substituted triazole (GH-1, 2, 4, 6, 12, 15, 21, 22, 24, 25, 27, 29); imidazole(GH-3, 11, 14, 16, 19, 20, 30); tetrazole (GH-23) and pyrazole (GH-5, 7-10, 13, 17, 18, 26, 28).

Theoretical calculation and prediction of the size for the above structure are carried out, and the predicted size is shown in the figure below:

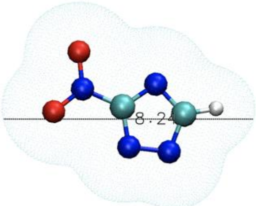

GH-1
***d* ≈ 8.2 Å**

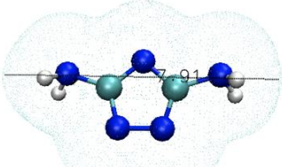

GH-2
***d* ≈ 7.9 Å**

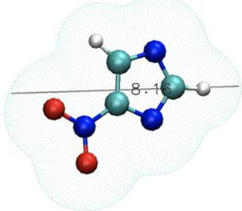

GH-3
***d* ≈ 8.2 Å**

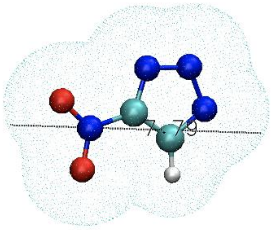

GH-4
***d* ≈ 7.8 Å**

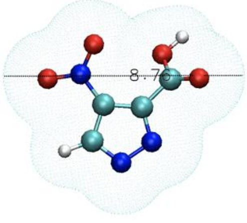

GH-5
***d* ≈ 8.8 Å**

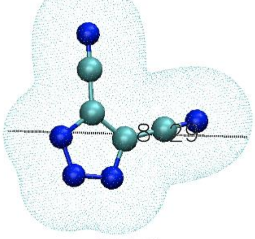

GH-6
***d* ≈ 8.3 Å**

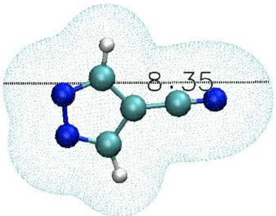

GH-7
***d* ≈ 8.4 Å**

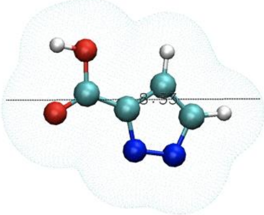

GH-8
***d* ≈ 8.3 Å**

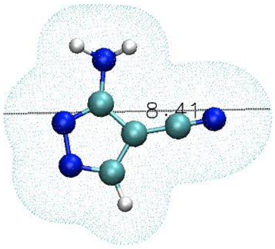

GH-9
***d* ≈ 8.4 Å**

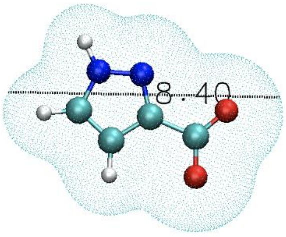

GH-10
***d* ≈ 8.4 Å**

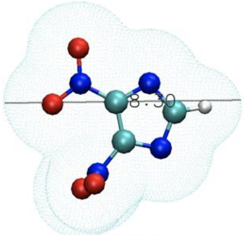

GH-11
***d* ≈ 8.7 Å**

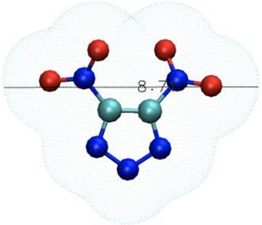

GH-12
***d* ≈ 8.7 Å**

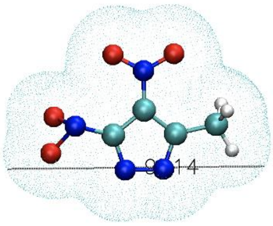

GH-13
***d* ≈ 9.1 Å**

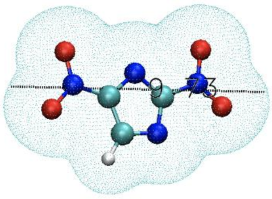

GH-14
***d* ≈ 9.7 Å**

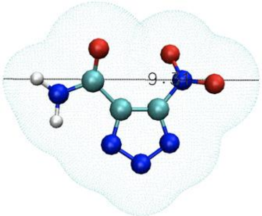

GH-15
***d* ≈ 9.3 Å**

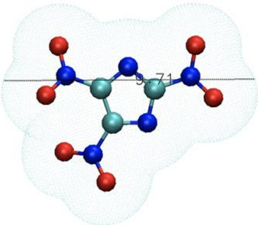

GH-16
***d* ≈ 9.7 Å**

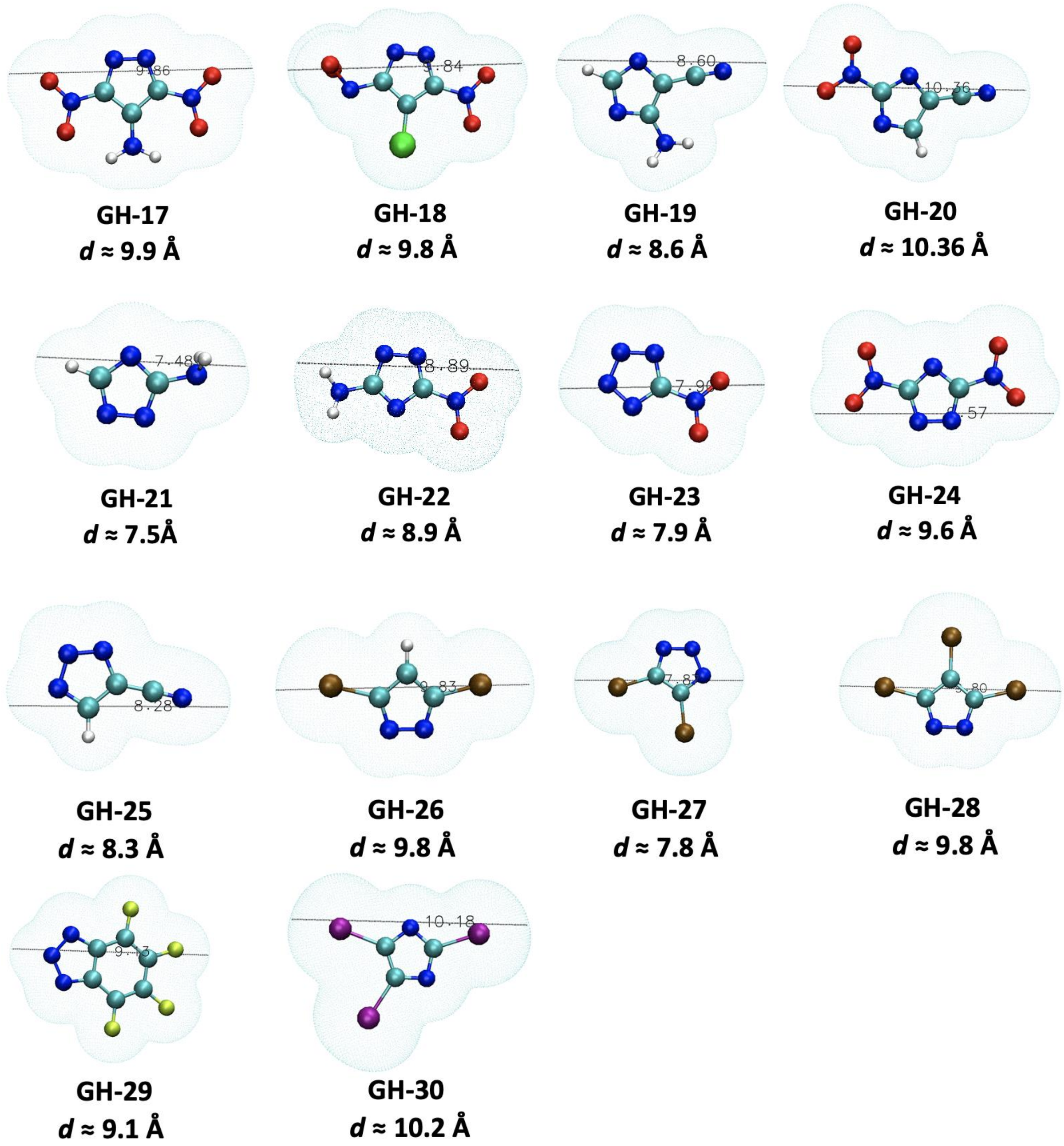

Supplementary Fig. 51. The predicted diameter of guest azole anions based on theoretical calculations. According to the size of the anion diameter, they can be summarized as follows: 7 <d ≤ 8 Å (GH-2, 4, 21, 23, 27); 8 < d ≤9 Å (GH-1, 3, 5-12, 19, 22, 25); 9 <d ≤ 10 Å (GH-13-18, 24, 26, 28, 29); 10 <d ≤ 11 Å (GH-20, 30).

From the above predictions, some of the smaller sized structures (GH-2, 4, 21, 23, 27) give ionic diameters less than 8 when the structures do not contain nitramine groups. Also, the predicted diameters of the guest GH-5-12,19, 22, and 25 are in the range of 8-9 Å. The other guest structures have diameters greater than 9 Å. To verify the correlation between the predicted size and the actual crystal packing, we try to synthesis those hydroxylammonium salts via the following general steps：

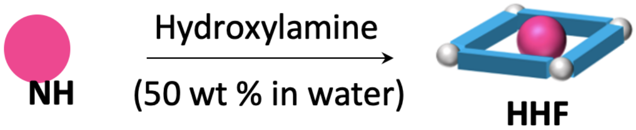

Supplementary Fig. 52. The general synthesis steps for HHF that N-rich heterocyclic azoles with acid hydrogen as the raw reagent.

With reference to the size prediction results, we try to prepare the crystals of hydroxylammonium salts corresponding to GH-5-12,19, 22, and 25 with the guest anion size in the range of 8-9 Å. Also to verify the feasibility of the strategy, some hydroxylammonium salts with guest anion sizes less than 8 Å and larger than 10 Å were selected for synthesis. The crystal data were successfully obtained for the following three compounds. They are HHF-H4 (hydroxylammonium 4-nitro-1,2,3-triazole-2-ide, hydroxylamine), HHF-H11 (hydroxylammonium 4,5-dinitroimidazol-1-ide) and HHF-H17 (hydroxylammonium 4-amino-3,5-dinitropyrazol-1-ide) (Supplementary Fig. 54. a, e, i).

In addition, we obtained ammonium salt crystals of GH-18 (ammonium 4-chloro-3,5-dinitropyrazol-1-ide, crystal-N5) during the incubation of the hydroxymonium salt crystals of GH-18 (Supplementary Fig. 53. g). During the preparation of such compounds, we observe that cyano-group turn into amino oximes (Supplementary Fig. 53. d). This may be one of the reasons why there are few hydroxylammonium salts containing cyano-zoles in the current CSD database. Also some of the structures (Supplementary Fig. 53. a, b, h, i, j).) may lack sufficient acidity to obtain hydroxylammonium salt crystals, and the corresponding neutral compounds are directly precipitated from the system. The above-mentioned problems also reflect the difficulty of preparing energetic hydroxylammonium crystals.

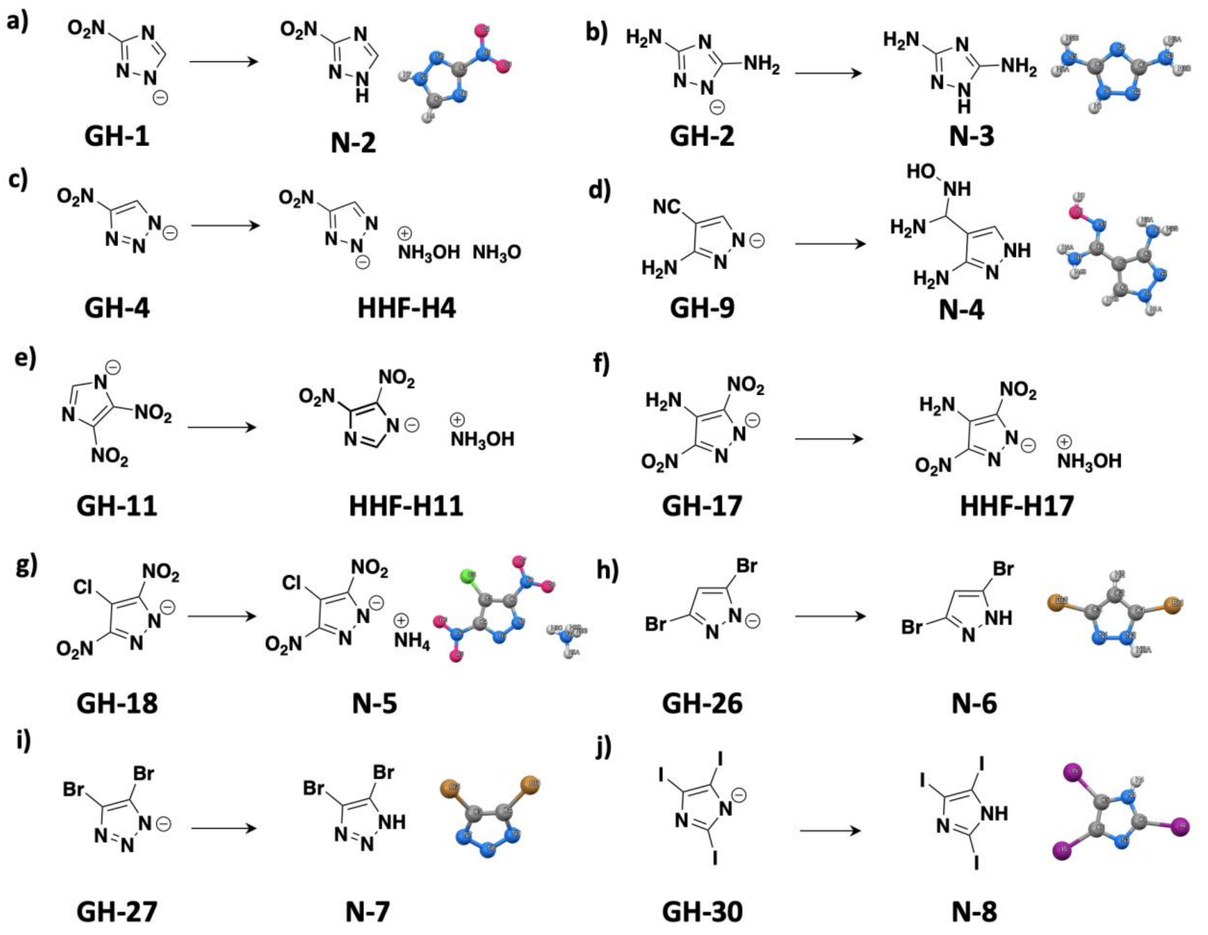

Supplementary Fig. 53. a) GH-1 as guest to form crystal N-2 (3-nitro-1H-1,2,4-triazole); **b)** GH-2 as guest to form crystal N-3 (1H-1,2,4-triazole-3,5-diamine); **c)** GH-4 as guest to form ammonium (hydroxylammonium 4-nitro-1,2,3-triazole-2-ide, hydroxylamine (HHF-H4); **d)** GH-9 as guest to form crystal N-4 (4-(amino(hydroxyamino)methyl)-1H-pyrazol-3-amine) **e)** GH-11 as guest to form crystal HHF-H11 (hydroxylammonium 4,5-dinitroimidazol-1-ide) **f)** GH-17 as guest to form ammonium (hydroxylammonium 4-amino-3,5-dinitropyrazol-1-ide (HHF-H17); **g)** GH-18 as guest to form crystal N-5 (ammonium 4-chloro-3,5-dinitropyrazol-1-ide); **h)** GH-26 as guest to form crystal N-6 (3,5-dibromo-1H-pyrazole) **i)** GH-27 as guest to form crystal N-7 (ammonium (4,5-dibromo-1H-1,2,3-triazole); **j)** GH-30 as guest to form crystal N-8 (2,4,5-triiodo-1H-imidazole).

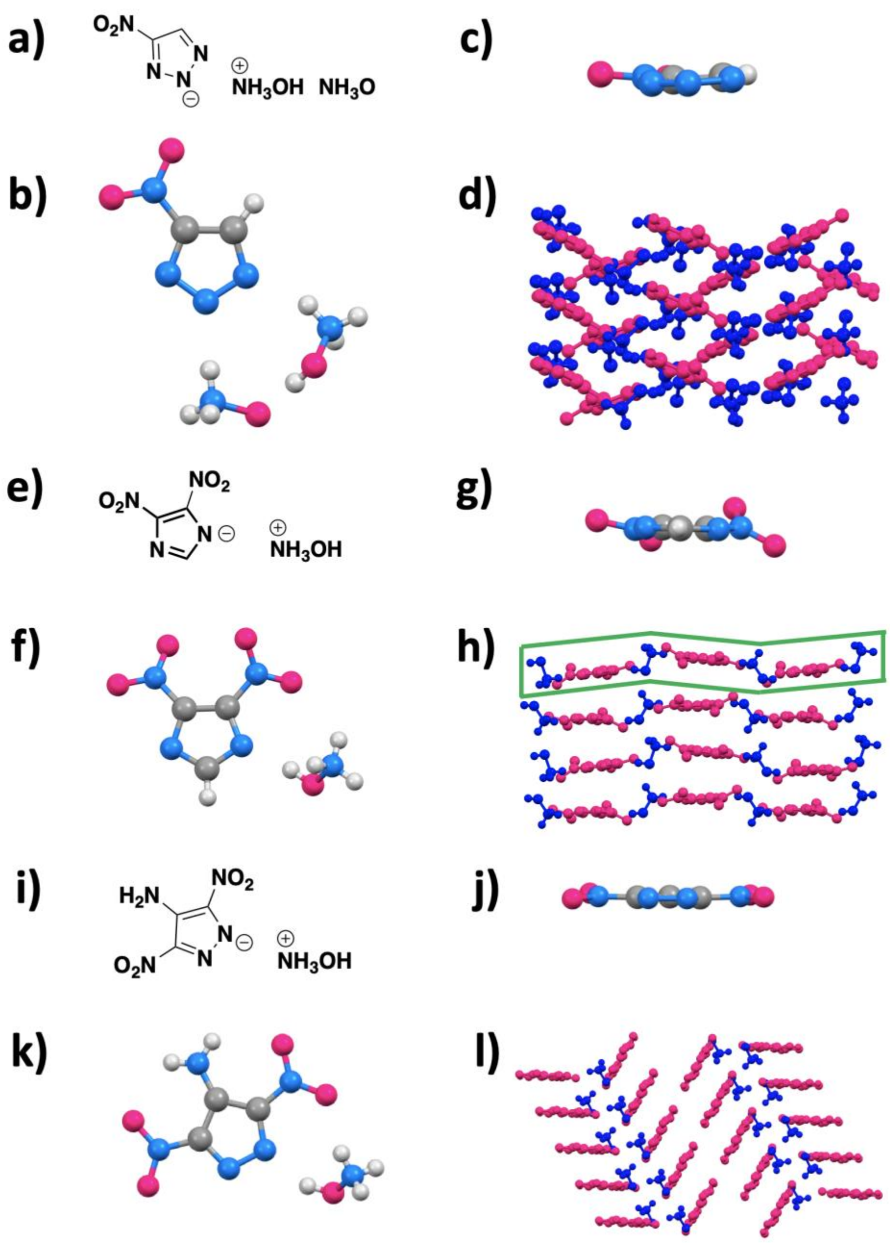

Supplementary Fig. 54. a), **e**) and **i**) Molecular structure of HHF-H4, HHF-H11 and HHF-H17**. b**), **f**) and **k**) Crystal structure of HHF-H4, HHF-H11 and HHF-H17. **c**), **g**) and **j**) Planar molecular geometry of HHF-H4, HHF-H11 and HHF-H17; **d**), **h**) and **l**) Crystal stacking structures of HHF-H4, HHF-H11 and HHF-H17.

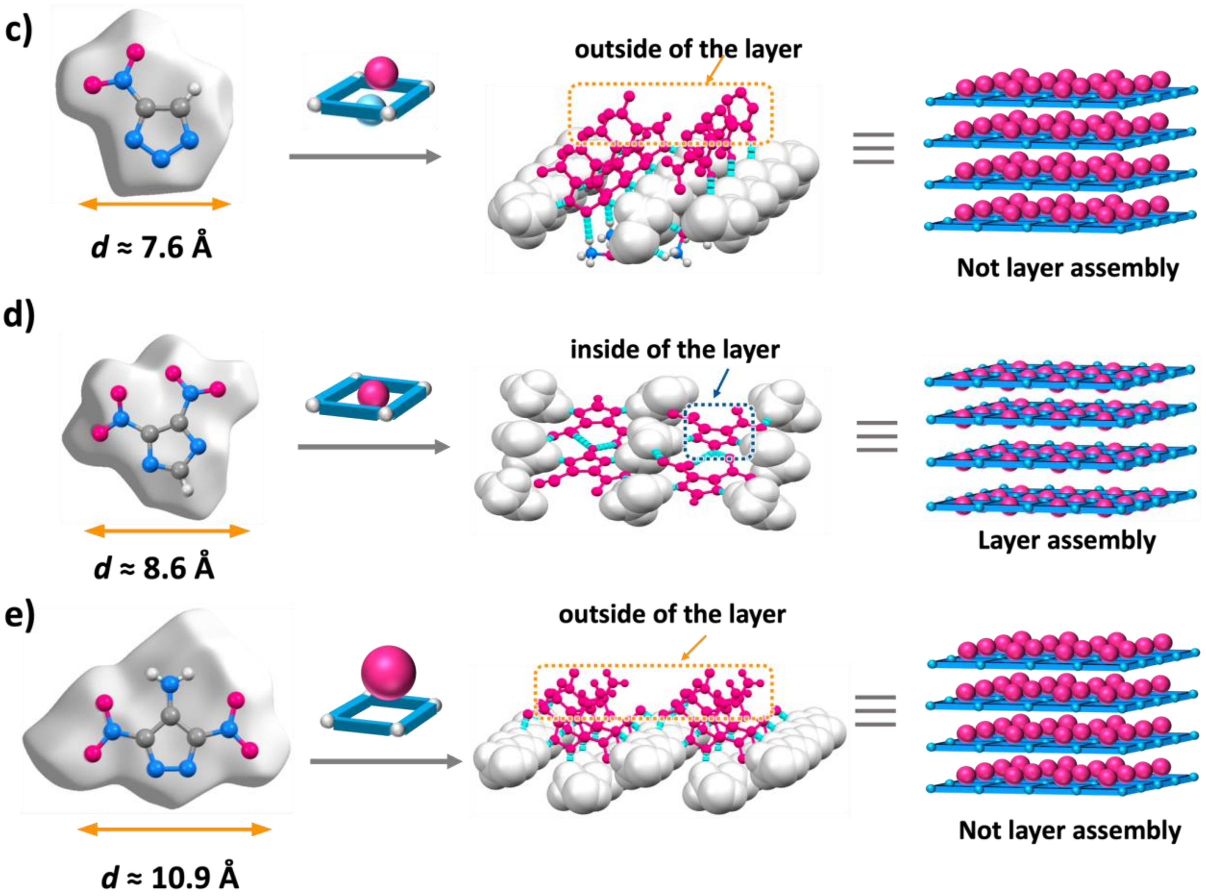

Supplementary Fig. 55. c) Layer assembly mode of HHF-H4 and its crystal packing diagram; **d**) Layer assembly mode of HHF-H11 and its crystal packing diagram; **e**) Layer assembly mode of HHF-H17 and its crystal packing diagram.

Using their crystal data, the Hershfeld surface diameters of the three guest anions were measured as GH-4 (7.6 Å), GH-11 (8.6 Å) and GH-17 (10.9 Å), which are very close to the diameters predicted by their theoretical calculations (GH-4 (7.8 Å), GH-11 (8.7 Å) and GH-17 (9.9 Å)).

To verify the relationship between the above types of anion guest size and crystal stacking, we analyzed their stacking mode, as shown in the figure above HHF-H4 not only does not possess a layer-by-layer stacking structure with hydroxylammonium units due to its small size, but also forms co-crystals with neutral hydroxylamine molecules. In contrast, HHF-H11 with a guest size of 8.6 Å shows an ordered crystal structure with the energetic guest wrapped by the hydroxylammonium hydrogen bonding framework, which possesses good layer-by-layer stacking in the three-dimensional structure, while HHF-H17 with a guest anion size of 11.0 Å does not enter the layers formed by the hydroxylammonium structure due to its larger size, which is consistent with our size matching strategy.

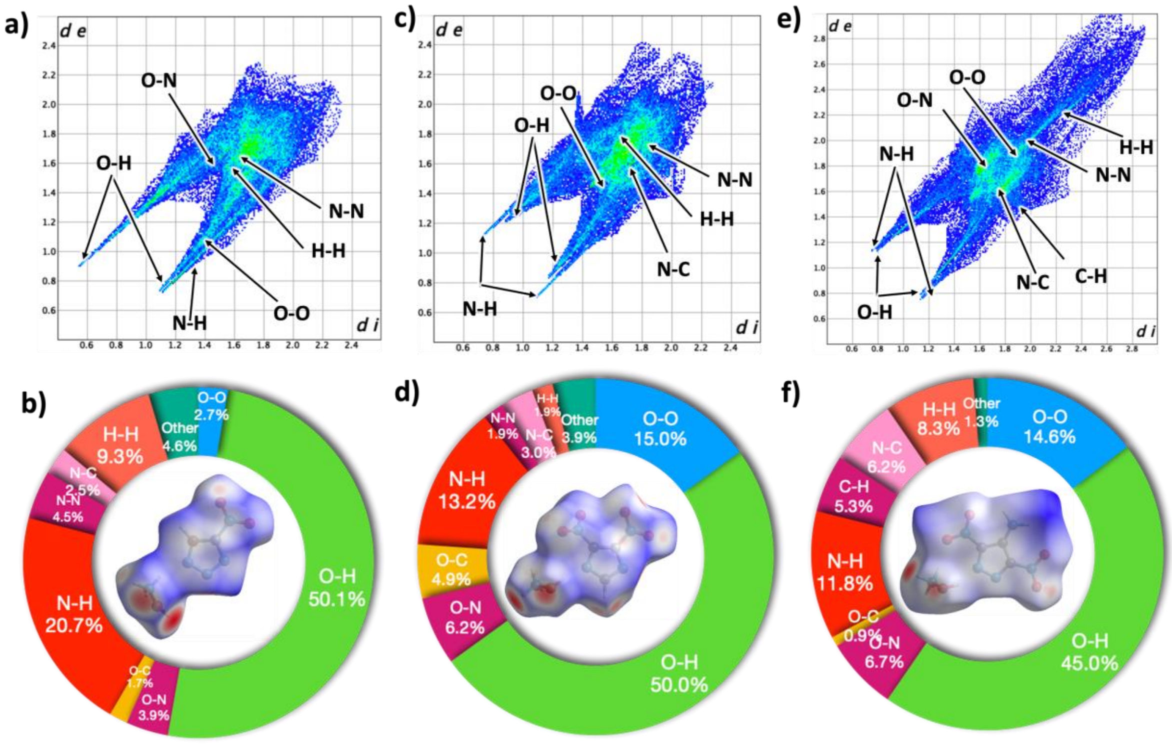

Supplementary Fig. 56. The 2D fingerprint plots in crystal stacking for HHF-H4 (**a**), HHF-H11 (**c**) and HHF-H17 (**e**); Pie graphs for HHF-H4 (**b**), HHF-H11 (**d**) and HHF-H17 (**f**) show the percentage contributions of the individual atomic contacts to the Hirshfeld surface, Hirshfeld surfaces (inside).

In summary, for the two guest systems mentioned above, by analyzing the relationship between the guest molecule size and the crystal stacking structure of seven examples obtained in this work, it can be concluded that the guest molecules of HHF-F (8.5 Å), HHF-N4 (7.6 Å), and HHF-H11 (8.6 Å), which have guest molecule sizes of 8-9 Å, are embedded in the hydroxylammonium composed of a hydrogen-bonded frameworks and exhibit layer-by-layer stacking, while the other over- or undersized guest molecules of HHF-N14 (10.1 Å), HHF-H17 (8.8 Å), HHF-T (11.1 Å), and HHF-H4 (7.6 Å) display a more disordered crystal structure. we believe the simple method to design energetic materials based on the size matched strategy could promote an ordered hydrogen bonded assembly between energetic molecules, thus displaying layer by layer packing in a solid state and forming a hydrogen bonded framework. And the above additional work also confirms this strategy can be applied more broadly.

VI Supplementary References

1. Liu, G., Li, H., Gou, R. & Zhang, C. Packing Structures of CL-20-Based Cocrystals. *Cryst. Growth Des.* **18**, 7065-7078 (2018).
2. Liu, G., Gou, R., Li, H. & Zhang, C. Polymorphism of Energetic Materials: A Comprehensive Study of Molecular Conformers, Crystal Packing, and the Dominance of Their Energetics in Governing the Most Stable Polymorph. *Cryst. Growth Des.* **18**, 4174-4186 (2018).
3. Turner, M. J. et al. Crystal Explorer (Version 17.5), University of Western Australia, (2017).
4. Zhao, Y. & Truhlar, D.G. A Density Functional That Accounts for Medium-Range Correlation Energies in Organic Chemistry. *Org. Lett.* **8**, 5753-5755 (2006).
5. Parr, R.G. & Yang, W. Density Functional Theory of Atoms and Molecules, Oxford University Press, New York, 1989.
6. Curtiss, L.A., Raghavachari, K., Trucks, G.W. & Pople, J.A. Gaussian-2 theory for molecular energies of first- and second-row compounds. *J. Chem. Phys.* **94**, 7221-7230 (1991).
7. Mehio, N., Dai, S. & Jiang, D. Quantum Mechanical Basis for Kinetic Diameters of Small Gaseous Molecules. *J. Phys. Chem. A*, **118**, 1150-1154 (2014).
8. a) Trouton, F. *Philos. Mag.* **18**, 54-57 (1884); b) Westwell, M. S., Searle, M. S., Wales, D. J. & Williams, D. H. Empirical Correlations between Thermodynamic Properties and Intermolecular Forces, *J. Am.Chem. Soc.* **117**, 5013-5015 (1995).
9. a) Jenkins, H.D.B., Tudela, D. & Glasser, L. Lattice Potential Energy Estimation for Complex Ionic Salts from Density Measurements. *Inorg Chem.* **41**, 2364-2367 (2002); b) Curtiss, L.A., Raghavachari, K., Trucks, G.W. & Pople, J. A. Gaussian-2 theory for molecular energies of first- and second-row compounds. *J. Chem. Phys.* **94**, 7221-7230 (1991).
10. a) Feng, Y., Chen, S., Deng, M., Zhang, T. & Zhang, Q. Energetic Metal-Organic Frameworks Incorporating NH_3OH^+ for New High-Energy-Density Materials. *Inorg. Chem.*, **58**: 12228-33 (2019); b) Lin, J., Chen, F., Xu, J. Zheng, F. & Wen, N. Framework-Interpenetrated Nitrogen-Rich Zn (II) Metal-Organic Frameworks for Energetic Materials. *ACS Appl. Nano Mater.* **2**, 5116-24 (2019).
11. Zhang, C. Investigation of the Slide of the Single Layer of the 1,3,5-Triamino-2,4,6-trinitrobenzene Crystal: Sliding Potential and Orientation, *J. Phys. Chem. B* **111**, 14295-14298 (2007).
12. Zhang, C., Wang, X. & Huang, H. π-Stacked Interactions in Explosive Crystals: Buffers against External Mechanical Stimuli. *J. Am. Chem. Soc.* **130**, 8359-8365 (2008).
13. Zhang, J., Mitchell, L.A., Parrish, D.A. & Shreeve, J. M. Energetic Salts with π-Stacking and Hydrogen-Bonding Interactions Lead the Way to Future Energetic Materials. *J. Am. Chem. Soc.* **137**, 1697-1704 (2015).
14. Grimme, S., Ehrlich, S. & Goerigk, L. Effect of the damping function in dispersion corrected density functional theory, *J. Comp. Chem.* **32**, 1456-1465 (2011).
15. Neese, F. & WIREs, The ORCA program system, *Comp. Mol. Sci.* **2**, 73-78 (2012).
16. Fischer, D., Klapötke, T.M. & Stierstorfer, J. Synthesis of 5-Aminotetrazole-1 N-oxide and Its Azo Derivative: A Key Step in the Development of New Energetic Materials. *Propellants Explos. Pyrotech.* **37**, 156-166 (2012).

17. Fischer, D., Klapötke, T.M., Piercey, D.G. & Stierstorfer, J. Synthesis of 5-Aminotetrazole-1 N-oxide and Its Azo Derivative: A Key Step in the Development of New Energetic Materials. *Chem. - Eur. J.* **19**, 4602-4613 (2013).
18. Klapötke, T.M., Krumm, B. & Riedelsheimer, C. Urazine-a Long Established Heterocycle and Energetic Chameleon. *Eur. J. Org. Chem.* **31**, 4916-4924 (2020).
19. Fischer, N., Klapötke, T.M., S Rappenglück, & Stierstorfer J. The Reactivity of 5-Cyanotetrazole towards Water and Hydroxylamine. *ChemPlusChem*. **77**, 877-888 (2012).
20. Klapötke, T.M., Piercey, D. G. & Stierstorfer J. The taming of CN7(-): the azidotetrazolate 2-oxide anion. *Chem.- Eur. J.* **17**, 13068-13077 (2011).
21. Sasidharan, N. , Kumar, A.S. & Hng, H.H., Combustion Studies of 4-Nitramino-1,2,4-Triazole (4-NRTZ) and Its Salts: High Impulse Nitrogenous Fuels for Propellant Composite Materials. *Chem.Select* **3**,12544-12551 (2018).
22. Gobel, M., Karaghiosoff, K., Klapötke, T.M. & Stierstorfer, J. Nitrotetrazolate-2N-oxides and the strategy of N-oxide introduction, *J. Am. Chem. Soc.*, **132**,17216-26 (2010).
23. Fischer, N., Klapötke, T.M., Piercey, D.G. & Stierstorfer, J. Hydroxylammonium 5-Nitriminotetrazolates. *Z. Anorg. Allg. Chem.* **638**, 302-310 (2012).
24. Li H., Huang M., Li J., Xu R. & Sun J. Synthesis and Crystal Structure of Hydroxylammonium Salt of 3-Nitro-1,2,4-triazol-5-one. *Hecheng Huaxue*, **15**, 714 (2017).
25. Klapötke, T.M., Kurz, M.Q., Schmid, P.C. & Stierstorfer, J. Energetic Improvements by N-Oxidation: Insensitive Amino-Hydroximoyl-Tetrazole-2N-Oxides, *J. Energ. Mater.* **33**, 191-201 (2015).
26. Fischer, D., Klapötke, T.M. & Stierstorfer, J. 1,5-Di(nitramino)tetrazole: High Sensitivity and Superior Explosive Performance. *Angew. Chem. Int. Ed.* **54**, 10299-10302 (2015).
27. Klapötke, T.M., Schmid, P.C. & Stierstorfer, J. Crystal Structures of Furazanes, *Cryst.* **5**, 418-432 (2015).
28. Bölter, M. F., Harter, A., Klapötke, T.M. & Stierstorfer, J. Isomers of Dinitropyrazoles: Synthesis, Comparison and Tuning of their Physicochemical Properties. *ChemPlusChem*, **83**, 804 (2018).
29. Fischer, N., Klapötke, T. M. & Stierstorfer, J. Energetic Nitrogen-Rich Salts of 1-(2-Hydroxyethyl)-5-nitriminotetrazole, *Eur. J. Inorg. Chem.* **28**, 4471- 4480 (2011).
30. Sun, Q., et al. Positional Isomerism for Strengthening Intermolecular Interactions: Toward Monocyclic Nitramino Oxadiazoles with Enhanced Densities and Energies, *Chem. Eng. J.* **427**, 130912 (2022).
31. Klapötke, T. M., Petermayer, C., Piercey, D G. & Stierstorfer, J. 1,3-Bis(nitroimido)-1,2,3-triazolate anion, the N-nitroimide moiety, and the strategy of alternating positive and negative charges in the design of energetic materials, *J. Am. Chem. Soc.* **134**, 20827-20836 (2012).
32. Tang, Y., Mitchell, L. A., Imler, G.H., Parrish, D.A. & Shreeve, J.M. Ammonia Oxide as a Building Block for High-Performance and Insensitive Energetic Materials, *Angew. Chem. Int. Ed.,* **56**, 5894-5898 (2017).
